# Oritatami: A Computational Model for Molecular Co-Transcriptional Folding

**DOI:** 10.3390/ijms20092259

**Published:** 2019-05-07

**Authors:** Cody Geary, Pierre-Étienne Meunier, Nicolas Schabanel, Shinnosuke Seki

**Affiliations:** 1 Computer Science Computation and Neural Systems Bioengineering Caltech, MS 136-93, Moore Building, Pasadena, CA 91125, USA; codyge@gmail.com; 2 Computer Science Dept, Hamilton Institute, Maynooth University, Co. Kildare, Ireland; pierre-etienne.meunier@mu.ie; 3 CNRS, École normale supérieure de Lyon (LIP), CEDEX 07, 69364 Lyon, France; 4 Computer and Network Engineering Dept, University of Electro-Communications, 1-5-1, Chofugaoka, Chofu, Tokyo 1828585, Japan; s.seki@uec.ac.jp

**Keywords:** natural computing, self-assembly, molecular folding

## Abstract

We introduce and study the computational power of Oritatami, a theoretical model that explores greedy molecular folding, whereby a molecular strand begins to fold before its production is complete. This model is inspired by our recent experimental work demonstrating the construction of shapes at the nanoscale from RNA, where strands of RNA fold into programmable shapes during their transcription from an engineered sequence of synthetic DNA. In the model of Oritatami, we explore the process of folding a single-strand bit by bit in such a way that the final fold emerges as a space-time diagram of computation. One major requirement in order to compute within this model is the ability to program a single sequence to fold into different shapes dependent on the state of the surrounding inputs. Another challenge is to embed all of the computing components within a contiguous strand, and in such a way that different fold patterns of the same strand perform different functions of computation. Here, we introduce general design techniques to solve these challenges in the Oritatami model. Our main result in this direction is the demonstration of a periodic Oritatami system that folds upon itself algorithmically into a prescribed set of shapes, depending on its current local environment, and whose final folding displays the sequence of binary integers from 0 to $N=2^{k}-1$ with a seed of size O(k). We prove that designing Oritatami is NP-hard in the number of possible local environments for the folding. Nevertheless, we provide an efficient algorithm, linear in the length of the sequence, that solves the Oritatami design problem when the number of local environments is a small fixed constant. This shows that this problem is in fact fixed parameter tractable (FPT) and can thus be solved in practice efficiently. We hope that the numerous structural strategies employed in Oritatami enabling computation will inspire new architectures for computing in RNA that take advantage of the rapid kinetic-folding of RNA.

## 1. Introduction

The process by which one-dimensional sequences of nucleotides or amino-acids acquire their complex three-dimensional geometries, which are key to their *function*, is a major puzzle of biology today. Understanding molecular folding will not only shed light on the origin and functions of the molecules existing in nature, it will also enable us to *control* the process more finely, and engineer artificial molecules with a wide range of uses, from performing missing functions inside living organisms, to producing precisely targeted drugs.

Biomolecular nano-engineering includes DNA self-assembly, which gave rise to an impressive number of successful experimental realizations, from arbitrary 2D shapes [1] to molecule cyclic machines [2], or counters [3]. First pioneered by Seeman [4], DNA nanotechnologies only really started to take off once a computer science model was devised by Winfree [5] to *program* molecular self assembly in a computer science way.

Since then, many models have been designed to refine different features of experiments: hierarchical self-assembly [6,7], modeling the absence of a *seed*, kinetic tile assembly [5,8], 3D and probabilistic tile assembly [9], among others.

However, the potential applications of this type of DNA technology inside cells are limited by the high thermal stability of the DNA duplex, where consequently DNA nanostructures are typically formed through the process of *annealing*: Namely, by heating the molecules up to high temperatures in a precisely controlled environment and then cooling them down at a precisely controlled rate. This  assembly paradigm allows researchers to design DNA structures with amazing precision by taking advantage of computational techniques to optimize the thermodynamics of strand-strand interactions, see for instance [10].

However at the same time the method requires precise control over temperature and other variables that are not always possible to control in every environment, such as for example within living cells. Other biopolymers such as RNA and proteins do not share this limitation, as nature uses both mediums to produce exquisite structures on a continuous basis. Researchers have successfully assembled functional nanoparticles and scaffolds out of these alternatives [11,12,13,14]. However, their assembly process is harder for us to program: while the shape that folds depends on both the sequence and the environment, the sequence is read *linearly* and expressed regardless of the environment. This  contrasts with more classical programming models such as Turing machines, or even tile assembly, which are able to *jump* to different parts of the program depending on the input. Another important difficulty is that even predicting the final folded shape of a biopolymer given the sequence is still the center of active research, especially for proteins [15,16,17,18,19].

In particular, a large body of computer science literature is focused on energy optimization, one of the main drivers of folding. For example, in different variants of the *hydrophobic-hydrophilic* (HP) model [20], it has been shown that the problem of predicting the most likely geometry (or *configuration*) of a sequence is NP-complete [21,22,23,24,25,26], both in two and three dimensions, and in different variants of the model.

The *kinetics* of folding, which is the step-by-step dynamics of the reaction, has been demonstrated by biochemists to play a major role in the final shape of molecules [27], and even play a *prevalent role* at the heart of the mechanism of RNA switches in biology [28]. In recent experimental results, researchers have even been able to *control* this mechanism to engineer various things out of RNA represented by the RNA origami architecture for single-stranded rectangular tiles [29], and followed by more complex structural motifs [30] and RNA-origami-based FRET system [31].

**Figure 1:**
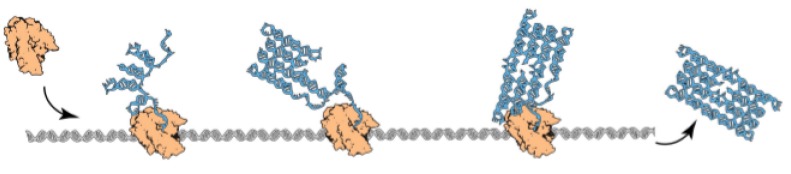
An RNA molecule folding over itself while being transcribed, as the experiments in [29].

This paper introduces a new model of computation and molecular folding inspired by RNA folding, intended to capture the kinetics of folding and model the experiments in [29]. In particular, it focuses on the *co-transcriptional* nature of RNA folding, whereby molecules fold concurrently while being transcribed (see Figure 1): in computer science terms, the folding process is a *local energy optimization*, or otherwise put, a *greedy algorithm*. A key limitation of this model is that the strand folds irreversibly once beads are locked into place, a simplification that makes working with the model tractable, but that also eliminates an important aspect of natural RNA folding—the possibility for structural rearrangement.

**Figure 2:**
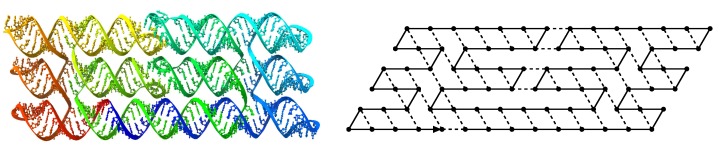
The design of a square, co-transcriptionally folded with RNA, and the corresponding path on the triangular lattice.

The first experimental results have used a standard benchmark: making simple shapes, such as squares (as shown for instance on Figure 2). With the new model introduced in this paper, our goal is twofold: first, explore the engineering possibilities of this mechanism, in order to make arbitrary shapes and structures. Then, the other aim of our study is to understand the complexity of sequence operations, to understand the computational processes which led to the creation of complex  molecular networks.

### 1.1. Main Contributions

In our model, called Oritatami, we consider a sequence of “beads”, which are abstract basic components, standing for nucleotides or even sequences of nucleotides (also called *domains*). In oritatami, only the latest produced beads of the molecules are allowed to move in order to adopt a more favorable configuration. The folding is driven by the respective attraction between the beads.

Our main construction is a *binary counter*. Counters are an essential component of many sophisticated constructions in biological computing, in particular in tile assembly [32,33]. Counters are also an important benchmark in experiments [3].

**Figure 3:**
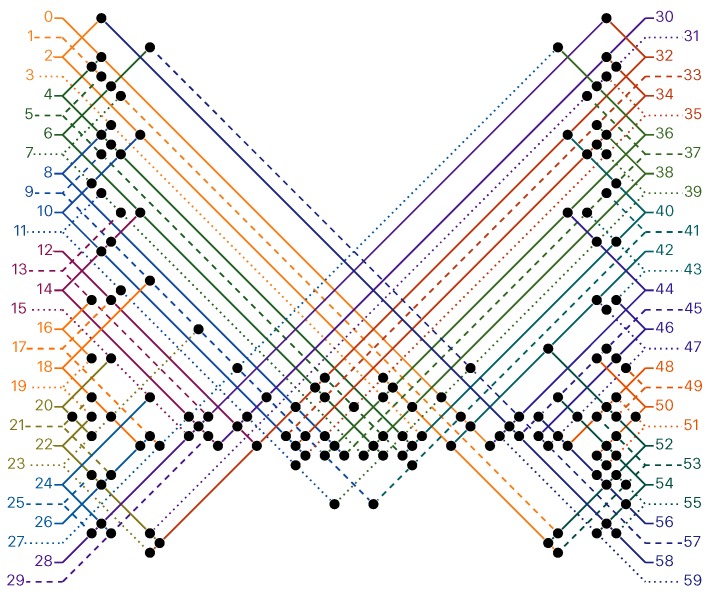
*The rule 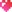 of the Counter oritatami system*: in this diagram, we have *b*
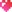
$b^{\prime }$ iff there is a bullet • at the intersection of one the two lines coming from *b* and from $b^{\prime }$; for instance, we have 4 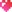 8 but not 4 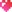 7.

**Theorem** **1.** *There is a fixed periodic sequence 0,1,…,59,0,1,… of period 60 whose rule is given in Figure 3, which, when started from a seed encoding an integer x in binary with at most 2k+1 bits for some k, folds into a structure encoding x+1, x+2, …, $2^{2k+1}-1$, on the successive rows of the triangular grid.*

We *prove the correctness* of this construction by designing an abstract module system to handle the complexity of the base mechanism of the model, which is about as low-level as assembly code in more standard computing models.

We then show a generic construction method in this model, which we applied to automate parts of the design of the counter. Moreover, this result helps understanding the computational complexity of sequence programming. Precisely, we prove two results in this direction:

**Theorem** **2.** *Designing a single sequence that folds into different target shapes in a set of surrounding environments, is NP-complete in the number of environments.*

More surprisingly, it turns out that there is an algorithm to solve this problem in time *linear in the length of the sequence*. This algorithm is also practical, as we were able to use it to find sequences for our main construction:

**Theorem** **3.** *The sequence design problem is FPT with respect to the length ℓ of the sequence: there is an algorithm linear in ℓ (but exponential in the number of environments) to design a single sequence that folds into the target shapes in the given environments.*

As Winfree’s thesis [5] on the very abstract tile assembly model did inspire many successful experimental works (see for instance [3,10,34,35,36]), we hope that the numerous structural strategies employed in Oritatami enabling computation will inspire new architectures for computing in RNA that take advantage of the rapid kinetic-folding of RNA.

### 1.2. Related Work

The oritatami system has received considerable attention since its proposal in the conference abstract of this paper [37]. As mentioned above, counters serve as a key component in tile self-assembly, especially for assembling shapes. Masuda et al. implemented a small oritatami system that serves as a 4-state automaton with output and integrated it with our binary counter (Theorem 1) into a larger-scale oritatami system towards the self-assembly of an arbitrary finite portion of Heighway dragon fractal [38]. Note that our binary counter works under inertial dynamics and they proposed a modified implementation working under oblivious dynamics with a different ratio of transcription speed to folding (technically speaking, ours works with delay 4 while theirs does with delay 3; delay is formally defined in Section 2). This illustrates the flexibility of oritatami designs. The transcript of their system is periodic as our binary counter; the period is linearly proportional to the size of the target portion. Demaine et al. [39] and Han and Kim [40] independently conducted more comprehensive studies of the shape self-assembly by oritatami systems recently. In particular, Demaine et al. proved that there is a universal set of 114 bead types with a rule set with which an arbitrary finite shape can be folded by an oritatami system, as long as the shape is scaled-up by a small factor.

The FPT algorithm (Theorem 3) has brought about useful modules not only for the binary counter but also for subsequent oritatami systems including the above-mentioned Heighway dragon fractal assembler, Satisfiability (SAT) tautology checker [41], and the time-efficient universal Turing machine simulation in [42]. The construction in [42] is the most intricate oritatami system implemented so far, developing much further the paradigms for co-transcriptional molecular programming introduced here.

Other variants of the rule design problem have been investigated by [43,44]. An alternative heuristic to the FPT algorithm to optimize the rule set has been proposed by Han and Kim [45].

## 2. Model and Main Results

Given two words $a,b\in B^{*}$, we denote by ab their concatenation.

### 2.1. Model

#### 2.1.1. Oritatami System

Oritatami is about the folding of finite sequences of beads, each from a finite set *B* of *bead types*, using an attraction rule 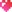, on the triangular lattice graph $T=(Z^{2},∼)$ where (x,y)∼(u,v) if and only if (u,v)∈{(x−1,y),(x+1,y),(x,y+1),(x+1,y+1),(x−1,y−1),(x,y−1)}.

A *configuration* *c* of a sequence $w\in B^{*}$ is a self-avoiding path of length n=|w| labelled by *w* in T, i.e., a path whose vertices $c_{1},\ldots ,c_{n}$ are pairwise distinct and labelled by the letters of *w*. We denote by $\mathrm{bt}\left(c_{i}\right)=w_{i}$ the bead-type of the *i*-th bead of *c*. A *partial configuration* of a sequence *w* is a configuration of a prefix of *w*. For any partial configuration *c* of some sequence *w*, an *elongation* of *c* by *k* beads is a partial configuration of *w* of length |c|+k. We denote by $C_{w}$ the set of all partial configurations of *w* (the index *w* will be omitted when the context is clear). We denote by $c^{\;▹k}$ the set of all elongations by *k* beads of a partial configuration *c* of a sequence *w* and by $c^{◃k}$ the singleton containing the prefix of length |c|−k of *c*.

An *oritatami system* O=(p, 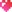 ,δ) is composed of (1) a (possibly infinite) *transcriptp*, which is a sequence of *beads*, of a type chosen from a finite set *B*, (2) an *attraction rule*, which is a symmetric relation 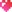 $\subseteq B^{2}$ and (3) a parameter δ called the *delay time*.

Given an attraction rule 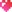 and a configuration *c* of a sequence *w*, we say that there is a *bond* between two adjacent positions $c_{i}$ and $c_{j}$ of *c* in T if $w_{i}$ 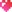 $w_{j}$. The *number of bonds* in a configuration *c* of *w*, written E(c), is the negation of the number of bonds within *c*: formally, E(*c*) = −|{(*i*,*j*) : *c_i_* ∼ *c_j_*, *j* > *i* + 1, and *w_i_*
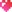
*w_j_*}|.

#### 2.1.2. Oritatami Dynamics

A *dynamics* for a sequence *w* is a function $D_{w}:2^{C_{w}}\to 2^{C_{w}}$ such that for all subset *S* of partial configurations of length *ℓ* of *w*, D(S) is a subset of the elongations by one bead of the partial configurations in *S* (thus, partial configurations of length ℓ+1).

Given an oritatami system O=(p, 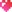, δ) and a *seed configuration* σ of a seed sequence *s* of length *ℓ*, a dynamics $D_{sp}$ gives at each time step the set of *favored nascent configurations* of transcript *p*, as a function of the set of favored nascent configurations at the previous time step. Initially, the set of favored nascent configurations is $\sigma^{\;▹\left(\delta -1\right)}$, that is all the possible elongations of the seed configuration by δ−1 beads. Then, the set of favored nascent configurations at step *t* is $D_{sp}^{t}\left(\sigma^{\;▹\left(\delta -1\right)}\right)$, that is the set of all elongations by (t+δ−1) beads of the seed configuration prolongated by the transcript according to dynamics D.

We explore greedy folding dynamics where only the most recently transcribed beads can move, all other beads remain in place. These still unsettled beads are said *nascent* and their number is controlled by the integer parameter δ (in most of this article, δ⩽4). We consider two different dynamics to model the “greedy” nature of the process:

**The** **inertial dynamics** was also called *hasty dynamics* in a preliminary version of this work [37]. It does not question previous choices but chooses the energy-minimal positions for the δ nascent beads among all elongations of the previously adopted partial configurations. It lets the δ−1 already placed nascent beads where they are and abandons the extension of a configuration if no extension with the newly transcribed bead allows to reach a lowest energy configuration available for the δ nascent beads.
  Formally, given a set of currently favored nascent configurations, I elongates each of them by one bead, and keeps the elongated configurations that have minimum energy among those who share the same prefix of length |σ|+t:
  $$I\left(S\right)=\underset{\gamma \in S^{◃\left(\delta -1\right)}}{⋃}\;\;\;\underset{c\in \gamma^{▹\gamma }⋂S^{▹1}}{\arg\;\min}\;E\left(c\right)$$
**The** **oblivious dynamics** is *oblivious* in the sense that it consists of always choosing the best available positions for the nascent δ beads regardless of the previously preferred choices, as opposed to I. Formally, O takes a set of currently favored nascent configurations, removes the last δ−1 positions from all of them, and selects the minimal energy configurations among all of their elongations by δ beads. Precisely:
  $$O\left(S\right)=\underset{\gamma \in S^{◃\left(\delta -1\right)}}{⋃}\;\;\underset{c\in \gamma^{▹\gamma }}{\arg\;\min}\;E\left(c\right)$$

Oritatami systems seem less governable under the oblivious dynamics than under the inertial dynamics, as a larger number of configurations are to be considered for energy minimization under the oblivious dynamics and also as previously discarded configurations may be reconsidered later on.

With the exception of Section 5, in this article, we choose the *inertial dynamics*. Our binary counter is described for the inertial dynamics in Section 3. Many subsequent articles [38,39,40,41,42,43,44,45,46] chose the oblivious dynamics. Masuda et al. [38] adapted our binary counter to the oblivious dynamics and used it as a component of their oritatami system folding arbitrary finite portion of Heighway dragon fractal.

**Determinism, Halt and Resulting Folding.** An oritatami system O=(p, 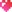 ,δ) is *deterministic* for dynamics D and seed σ of sequence *s* if for all i≥1, the position of the *i*-th bead of *p* is uniquely determined at time *i*, i.e., if for all i≥1, $|\{c_{|\sigma |+i}:c\in D_{sp}^{i}\left(\sigma^{\;▹\left(\delta -1\right)}\right)\}|=1$.

We say that O *stops* at time *t* with seed σ and dynamics D, if $D_{sp}^{t}\left(\sigma^{\;▹\left(\delta -1\right)}\right)=\emptyset$ and $D_{sp}^{z}\left(\left\{\sigma \right\}\right)\neq \emptyset$ for z<t. If O stops at time *t*, the set $D_{sp}^{t}\left(\sigma^{\;▹\left(\delta -1\right)}\right)$ is the *result* of the folding process; if it consists of a single configuration *c*, we say that *c* is the *resulting folding* and that the oritatami system O*folds deterministically* into configuration *c*. If the transcript *p* is finite, the folding process will stop at time t=|p|−δ+1 or before in case of a geometric obstruction (no more elongation is possible because the configuration gets trapped in a closed area). If the transcript *p* is infinite, the folding process may only stop because of a geometric obstruction.

**Figure 4:**
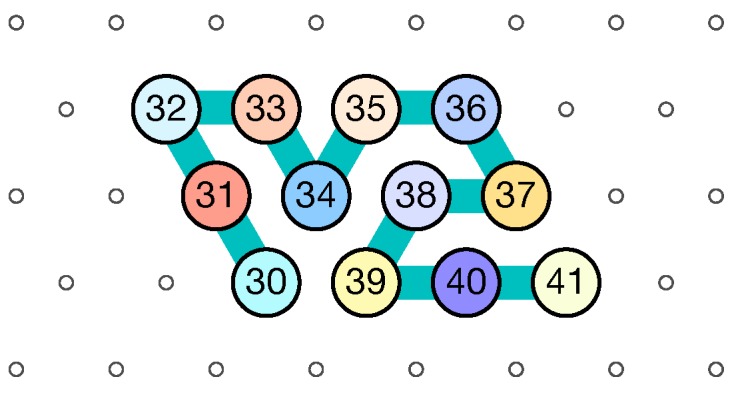
A configuration encoded by $30\overset{\mathrm{NW}}{{}_{↖}}31\overset{\mathrm{NW}}{{}_{↖}}32\vec{{}_{E}}33\overset{↘}{{}_{\mathrm{SE}}}34\overset{\mathrm{NE}}{{}_{↗}}35\vec{{}_{E}}36\overset{↘}{{}_{\mathrm{SE}}}37\overset{\leftarrow }{{}_{W}}38\overset{↙}{{}_{\mathrm{SW}}}39\vec{{}_{E}}40\vec{{}_{E}}41$.

**Notation for configurations.** We will use $a\overset{\mathrm{NW}}{{}_{↖}}b$, $a\overset{\mathrm{NE}}{{}_{↗}}b$, $a\vec{{}_{E}}b$, $a\overset{↘}{{}_{\mathrm{SE}}}b$, $a\overset{↙}{{}_{\mathrm{SW}}}b$, $a\overset{\leftarrow }{{}_{W}}b$ to denote a configuration consisting of two beads with types *a* and *b* where *b* is placed respectively at the NW, NE, E, SE, SW or W of *a*. As an example, the configuration in Figure 4 is described as $30\overset{\mathrm{NW}}{{}_{↖}}31\overset{\mathrm{NW}}{{}_{↖}}32\vec{{}_{E}}33\overset{↘}{{}_{\mathrm{SE}}}34\overset{\mathrm{NE}}{{}_{↗}}35\vec{{}_{E}}36\overset{↘}{{}_{\mathrm{SE}}}37\overset{\leftarrow }{{}_{W}}38\overset{↙}{{}_{\mathrm{SW}}}39\vec{{}_{E}}40\vec{{}_{E}}41$.

## 3. Folding a Binary Counter

### 3.1. General Idea of The Construction

*Our construction works with δ=4.* The counter is implemented by folding the periodic sequence of bead types 0,1,…,58,59,0,1,… with period 60. Our construction proceeds in zig-zags as the classic implementation of a counter with a sweeping Turing machine whose head goes back and forth between the two ends of the coding part of the tape. Each pass is 3-rows thick and folds each part of the molecule into a parallelogram of size 4×3 or 6×3 except for the last and the first parts of each pass which are folded into parallelograms of size 3×6 to accomplish the U-turn downwards and start the next pass. The *zig pass*, folding three rows at a time from right to left, computes the carry propagation in the current value of the counter. The *zag pass*, folding three rows at a time from left to right, writes down the bits of the newly incremented value, and gets the folding to resume at the right-hand side of the configuration.

The molecule is semantically divided into 4 parts, called *modules*:

- Module A (beads 0–11, in blue in all figures): the First Half-Adder
- Module B (beads 12–29, in red in all figures): the Left-Turn module
- Module C (beads 30–41, in blue in all figures): the Second Half-Adder
- Module D (beads 42–59, in red in all figures): the Right-Turn module

**Encoding.** The current value of the counter is encoded in standard binary with the most significant bit to the left. Each bit is encoded into a specific folding of the modules A and C of the molecule in the rows corresponding to a zag pass: namely folding A0 and C0 for 0, and A1 and C1 for 1. During the zig pass, the *value of the carry* is encoded by the position of the molecule when it starts to fold Module A or C: carry=0 if Module A or C starts to fold in the top row; carry=1 if Module A or C starts to fold from the bottom row.

**In the zig pass (←),** modules A and C “read” from the row above the value encoded into the folding in the row above during the previous zag-phase (or in the seed configuration for the first zig pass), and fold into a shape (called a *brick*, see Section 4.1) A00, A10, A01, A11 or C00, C10, C01, C11 accordingly where Axc is the brick corresponding to the case where *x* is the bit read in the row above and *c* is the carry. In the zig pass, modules B and D just propagate the carry value (0 or 1, i.e., start from top or bottom row) output by the preceding module A or C to the next.

When the zig pass reaches the leftmost part of the row on top, the beads there forces the module B to adopt the Left-turn shape which reverses the folding direction and starts the next zag pass.

**In the zag pass (→),** modules A and C “read” the bricks above Axc or Cxc and folds into the bricks that encodes the corresponding bits, namely A*y* or C*y* where y=(x+c)mod2. There are no carry propagation and all the modules B and D fold into the same brick B2 or D2 in this pass.

When the molecule reaches the rightmost part of the row on top of it, the special beads there force the module D to fold into the Right-turn brick which reverses the folding direction and starts the next zig pass.

### 3.2. The First Two Passes of The Folding

Let’s run the first passes of the 3 bits counter to get acquainted with the process.

**Figure 5:**
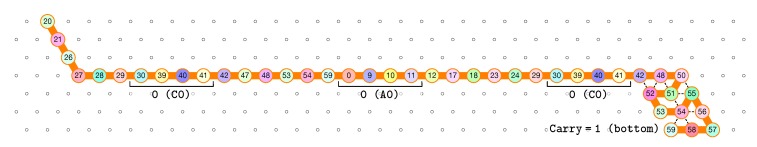
The seed configuration for the 3-bits counter encoding the three bits 000 as the initial value of the counter.

**The seed configuration** is shown in Figure 5. The seed configuration for the (2k+1)-bit counter is composed of 4k+3 parts:

- The first part $20\overset{↘}{{}_{\mathrm{SE}}}21\overset{↘}{{}_{\mathrm{SE}}}26\overset{↘}{{}_{\mathrm{SE}}}27\vec{{}_{E}}28\vec{{}_{E}}29$, made of beads from Module B, encodes a sequence that will trigger the carriage return at the end of the next zig pass.
- The central part consists in *k* repetitions of the same sequence of 4 patterns, plus an extra repetition of the first pattern at the end (the central part consists thus in 4k+1 parts in total):
  - $30\vec{{}_{E}}39\vec{{}_{E}}40\vec{{}_{E}}41$ encoding a bit 0 using beads from Module C,
  - followed by $42\vec{{}_{E}}47\vec{{}_{E}}48\vec{{}_{E}}53\vec{{}_{E}}54\vec{{}_{E}}59$ encoding nothing but padding using beads from Module B,
  - followed by $0\vec{{}_{E}}9\vec{{}_{E}}10\vec{{}_{E}}11$ encoding a bit 0 using beads from Module A,
  - followed by $12\vec{{}_{E}}17\vec{{}_{E}}18\vec{{}_{E}}23\vec{{}_{E}}24\vec{{}_{E}}29$ encoding nothing but padding using beads from Module D.
  Note the symmetry by a shift of 30 of the beads values in the patterns involving Modules A and C, and Modules B and D.
- The last part $42\vec{{}_{E}}48\vec{{}_{E}}50\overset{↙}{{}_{\mathrm{SW}}}51\overset{\leftarrow }{{}_{W}}52\overset{↘}{{}_{\mathrm{SE}}}53\vec{{}_{E}}54\overset{\mathrm{NE}}{{}_{↗}}55\overset{↘}{{}_{\mathrm{SE}}}56\overset{↘}{{}_{\mathrm{SE}}}57\overset{\leftarrow }{{}_{W}}58\overset{\leftarrow }{{}_{W}}59$, made of beads from Module D, encodes a sequence that will first initiate the next zig pass and later trigger the carriage return at the end of the next zag pass.

Note that the seed configuration ends at the bottom row of the upcoming zig pass, which encodes thus that initially the carry is 1.

**Figure 6:**
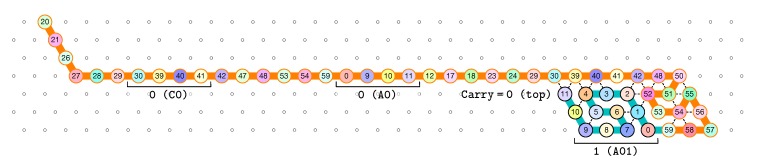
The folding of the first module, A: starting with a carry 1, encoded by the position of the first bead (on the bottom row), this module “reads” a 0 from the seed by binding to the seed, and folds into A01, encoding a 1 with no carry propagation, as encoded by the position of the last bead (on the top row of the module).

**The first zig pass (←).** Each zig pass starts with a carry equal to 1, i.e., starts folding from the bottom row. In the first zig pass, the first module A (see Figure 6) folds into the brick A01, encoding the bit 1=0+1 with no carry propagation, as a consequence of the carry being 1 and of reading the first bit, 0, from the seed above. Note that the folding A01 ends at the top row, encoding that the carry is now 0. The reading of the bit from the seed is accompl details in Section 3.3.

**Figure 7:**
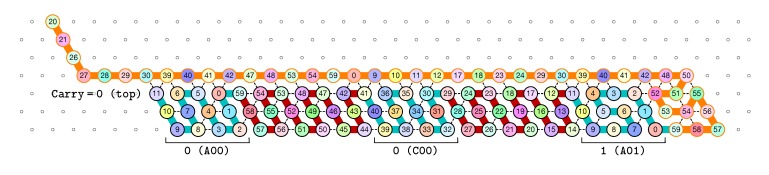
The folding of the central part of the first zig pass in the 3-bits counter.

Then, as illustrated in Figure 7, the next modules B, C, D, and A fold into shapes B0, C00, D0 and A00 respectively: B0 and D0 meaning that no carry is propagated (they start and end on the top row); and C00 and A00 meaning that the (input) carry is 0 and the bit read from the seed is 0.

**Figure 8:**
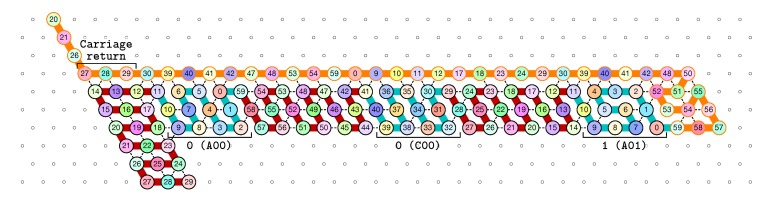
In our construction, the leftmost three beads of any configuration are different from the other beads the left U-turn module binds to inside the zig or zag pass: when the left U-turn module folds next to these bead types, it “triggers” the production of an actual U-turn.

Finally, as illustrated in Figure 8, the last module, B, of the zig pass binds to the 3-beads long carriage-return pattern at the leftmost part of the seed and folds into the shape BT conducing the molecule to go down by 6 rows, reverse direction and start the following zag pass. Note that the bottom of the shape BT contains the exact same carriage-return pattern.

**Figure 9:**
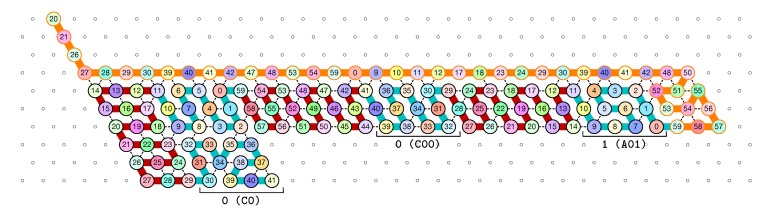
During the zag pass, all modules start from the bottom row, computing the value of each new bit by rewriting shapes A00 and A11 as C0, C00 and C11 as A0, A10 and A01 as C1, and C10 and C10 as A1.

**Figure 10:**
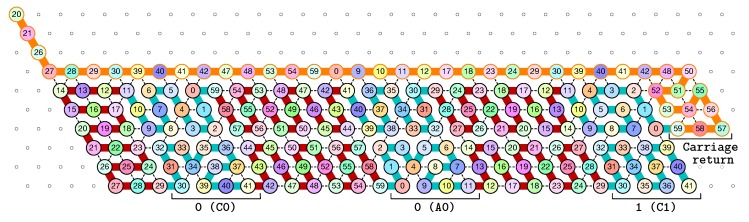
At the end of the first zag pass, the new value of each bit have been encoded into shapes: A0 or C0 for bits equal to 0, A1 or C1 for bit equal to 1.

**The first zag pass (→).** The zag pass is mostly a normalization pass as illustrated in Figure 9 and Figure 10. It progresses from left to right and computes the new value of each bit by rewriting each shape A00 and A11 as C0, C00 and C11 as A0, A10 and A01 as C1, and C10 and C10 as A0. Shapes A0 and C0 encode 0, and Shapes A1 and C1 encode 1, both to be read during the upcoming zig pass. Modules B and D just fold into the shapes B2 and D2 respectively, encoding nothing but padding.

**Figure 11:**
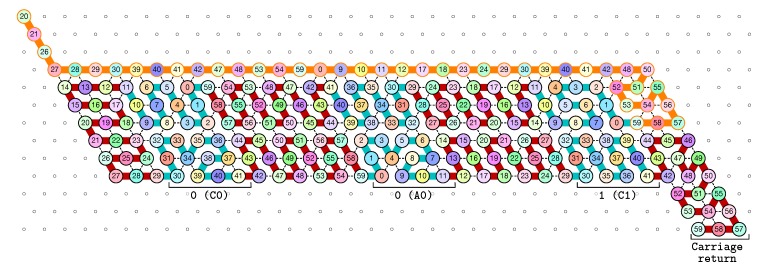
Finally, at the end of the first zag pass, the last module D binds to the carriage-return pattern in the seed and fold into the shape DT to accomplish the right U-turn from which the next zig pass can start.

Finally, as illustrated in Figure 11, the last module, D, of the zag pass binds to the 3-beads long carriage-return pattern in the rightmost part of the seed and folds into the shape DT conducing the molecule to go down by 6 rows, reverse direction and start the next zig pass. Note that, as for the shape BT, the bottom of the shape DT contains the exact same carriage-return pattern.

**Figure 12:**
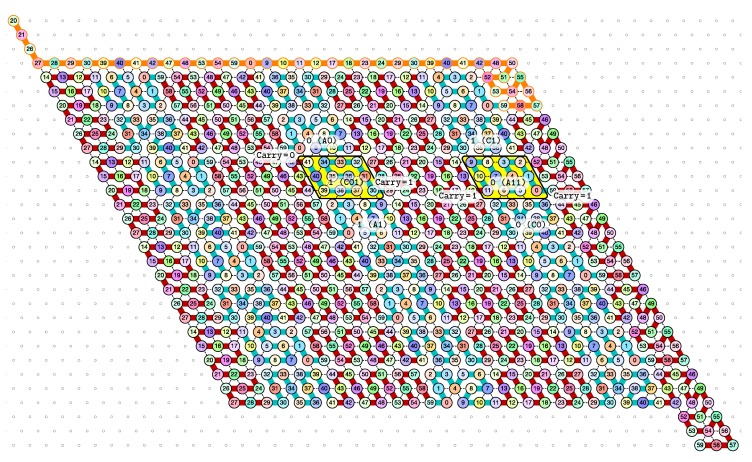
The folding of the 3-bits counter upto value 3=011 in binary. Observe the carry propagation in the second zig pass.

Figure 12 shows the 3-bits counter folded upto the value 3=011 in binary. One can observe the shape A11 in the second zig pass. A11 corresponds to reading a 1 with a carry 1 which propagates the carry: indeed, the folding ends at the bottom row which propagates the carry to the next module C which folds into C01 as it reads a 0 from above with carry 1. Note that shape A11 is then rewritten as C0 in the following zag pass below.

### 3.3. How Does Computation Take Place: Modules, Functions, States and Environment

Each module A, B, C or D implements various “functions” that are “called” when the molecule is folded. Which function is called depends on two things:

**the** **current “state” of the molecule:** here, the *state* is whether the carry is 0 or 1. As mentionned earlier this is encoded in the position of the molecule when the module starts to fold: it starts in the top row if the carry is 0; in the bottom row if the carry is 1.
**the** **local environment of the molecule:** the *environment*, i.e., the beads already placed around the current area where the folding takes place, acts as the memory in the computation.

The position where the folding of a module starts, determines which beads of a given module will be exposed to and interact with the environment. Then, by creating bonds (or not) with the environment, each module will adopt a specific shape. Therefore, the possible binding schemes will be different depending on this initial position. Similarly, depending on the beads already placed in the environment, the part of the module exposed to it will adopt one form or another depending on how many bonds it can create with the environment. Adopting the language of computer science: the position at which a module starts to fold, determines which *function* of the module is called; the function then *reads the input* encoded by the beads already placed in the environment.

**Figure 13:**
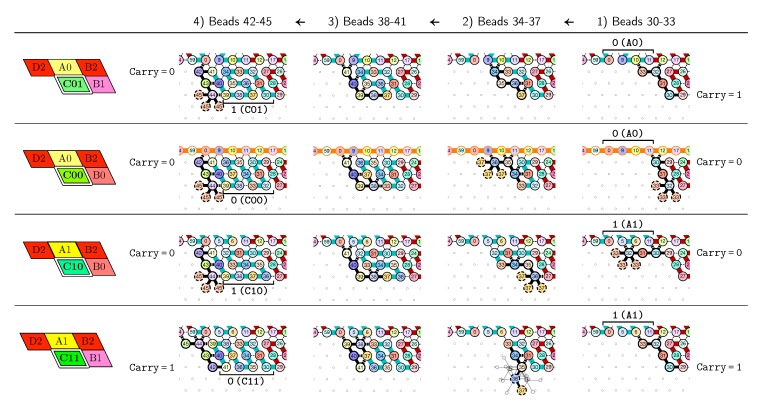
An illustration of how the module C applies a different function which results in different foldings according to the initial state of the molecule (carry = 0 or 1) at the beginning of the folding of the module, and to the environment (the bit 0 or 1 encoded) read above by the function. This figure is meant to be read from right to left (zig pass ←).

Figure 13 provides a precise description on how the function of the Half-Adder Module C are implemented in the zig pass. As the zig pass goes from right to left, the figure is meant to be read from right to left. In the zig pass, Module C implements two functions: (1) Add 1 to the bit above and propagate the carry if needed, or (2) Copy the bit above unchanged. Add is called if the carry is 1 at the beginning of the folding and Copy is called if the carry is 0. The following step-by-step description of the folding explains how:

**Beads** **30–33 (rightmost column in Figure 13):** *if the carry is 0 at start,* then bead 30 is able to bind with beads 11 and 12 from the environment and depending on whether the input encodes a bit 0 or 1, bead 32 will be able to bind either to 28 or to 5 and 6 respectively. *Whereas if the carry is 1,* then bead 30 cannot reach the beads 11 and 12. Thus, these are beads 31 and 32 that will bind with beads 10 and 12 from the environment, giving to the molecule a completely different shape.
**Beads** **34–37 with carry = 0 at start:** as bead 34 is attracted by both beads 30 and 31, the molecule folds upon itself similarly but with a different rotation depending on whether it has read a 0 or a 1 in the environment above: vertically if it has read a 0, horizontally if it has read a 1. Bead 36 attracted by beads 9 and 27 fixes the end of the tip in place leaving bead 37 free to move for now.
**Beads** **34–37 with carry = 1 at start:** Bead 34 is attracted by beads 9 and 10 encoding a bit 0 above which allows beads 36 and 37 to bind with 31 as well, but prefers to bind with 31 together with 35 otherwise. This induces two different shapes: the beginning of a “wave” pattern (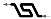) if the bit read above is 0; or the beginning of a “switchback” pattern (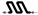) if the bit read is 1.
**Beads** **38–41, without carry propagation (carry = 0, or carry = 1 and bit read = 0):** in these three cases the folding of the beads 38–41 starts from the same position. As the environment is different for each of them, we could design the rule so that this part of the module prefers to adopt the same shape, climing along the part already folded to the top row to start the next module with a carry =0.
**Beads** **38-41, with carry propagation (carry = 1 and bit read = 1):** because the switchback pattern is upside down in this case, bead 37 stays low and bead 38 can firmly attach to the top with beads 5, 6 and 33, and the tip of the module folds downwards as 40 and 41 are attracted by 37. This ensures that the folding of the module ends at the bottom row, propagating the carry =1 to the next module.

Note that the bottom rows of the four resulting foldings differ significantly: 39–38–37–30 for C01, 39–38–33–32 for C00, 39–38–37–36 for C10, and 41–36–35–30 for C11. This will allow to distinguish between them in the following zag pass to write the correct bit for the new value of the counter.

## 4. Proof of Correctness (Theorem 1)

### 4.1. Overview

As mentioned earlier, each 60-period of the molecule is semantically divided into four modules:

- Module A (beads 0–11): the First Half-Adder
- Module B (beads 12–29): the Left-Turn module
- Module C (beads 30–41): the Second Half-Adder
- Module D (beads 42–59): the Right-Turn module

Depending on the environment in which they fold upon themselves, each of these modules may adopt the following different configurations. We call *bricks* the various configurations each module will adopt in the final folding, as they are the bricks upon which the whole folding is built.

There are six bricks for Modules A and C:

- Axc and Cxc in the zig pass where x,c∈{0,1} are the bit read from the brick above (A*x* or C*x*) and the (input) carry from the preceding module (B*c* or D*c*);
- A*y* and C*y* in the zag pass where *y* is the bit written: namely y=x+cmod2 if the brick above is Axc or Cxc.

There are four bricks for Modules B and D:

- B$c^{\prime }$ and D$c^{\prime }$ in the zig pass where $c^{\prime }\in \left\{0,1\right\}$ is the carry output by the preceding brick Cxc or *A*xc, namely $c^{\prime }=x\wedge c$;
- and B2 and D2 in the zag pass.

**Figure 14:**
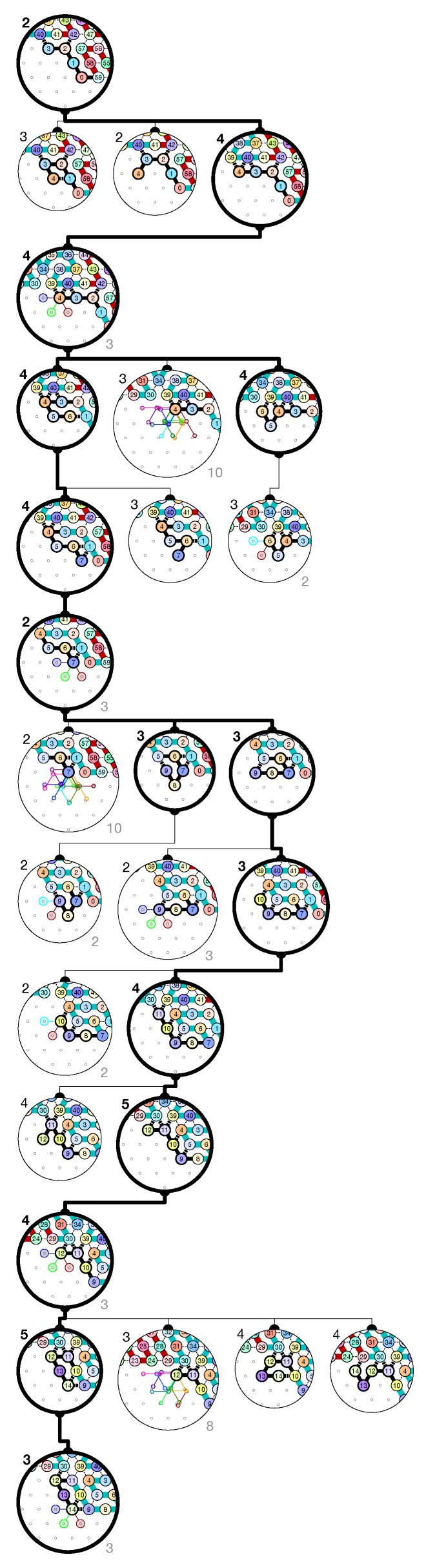
The folding certificate for the brick A01 in the environment: 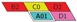.

**Figure 15:**
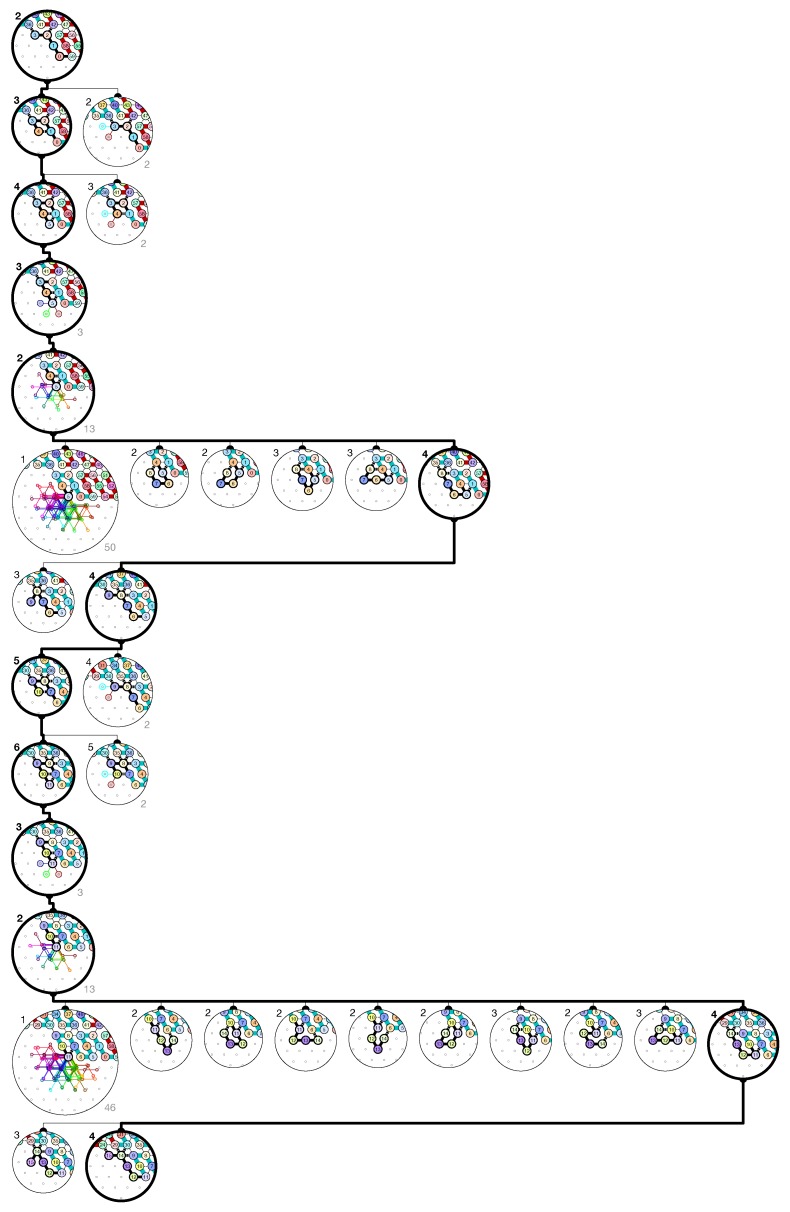
The folding certificate for the brick A11 in the environment: 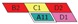.

The proof works in three stages: (1) we describe the targeted final folding and show that this target folding implements correctly a binary counter: i.e., that the bricks implement correctly the relationships between the *y*, *x*, *c* and $c^{\prime }$ described above. Then, we show that the molecule folds indeed as prescribed by the target folding. We proceed in two more stages: (2) we enumerate all the possible surroundings for each module in the target folding; and (3) we show by recurrence that each module folds into the desired brick in each of the possible surroudings. For  stage 3, our program produces automatically a human-readable *folding certificate*, which shows the correctness of each step of the folding in each possible environment. It consists in displaying in a compact but readable way, all the possible extensions of the current configuration, and displaying for each of them the number of bonds created (the number in the north-east corner); the maximum bonding configurations are displayed in bold. For readability, we group together extensions when they share a common path up to their last bond (the number of paths grouped is then displayed in the south-east corner for cross-checking). The resulting enumeration is displayed as a tree where only the maximum bond configuration (in bold) gives birth to configurations at the next level. Following the bold path in the tree certificate shows the folding adopted by the molecule. See Figure 14 and Figure 15 for two examples of folding certificates for Brick A01 in the surrounding consisting of the bricks B2, C0, D2 and D1, and for Brick A11 in the surrounding consisting of the bricks B2, C1, D2 and D1. Figure 14 corresponds to the half-adder *A* reading a 0 from above (Brick C0) and a carry from the right (Brick D1); whereas Figure 15 corresponds to the half-adder *A* reading a 1 from above (Brick C1) and a carry from the right (Brick D1). Note that the output configuration is exiting the region from the top for Brick A01 (no carry is propagated) and from the bottom for Brick A11 (a carry is propagated).

In the next subsections, we use our new tools to prove the correctness of the Oritatami System presented in Section 3. Precisely, we show that, starting from a proper seed of length 21+20k, the 60-periodic molecule ${\left(0,\ldots ,59\right)}^{\omega }$ folds upon itself into $2\cdot 2^{2k+1}-1$ rows of height 3 implementing a (2k+1)-bits counter counting from 0 to $2^{2k+1}$.

### 4.2. Description of the Final Configuration (I.e., the Resulting Folding)

Let us first describe each of the possible bricks for each module:

- Module *A*, First Half-Adder (beads 0–11): 
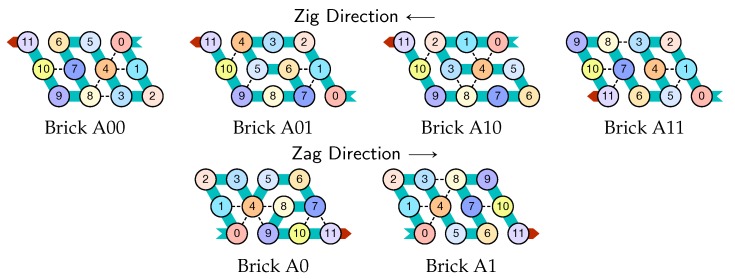
- Module *B*, Left-Turn module (beads 12–29) 
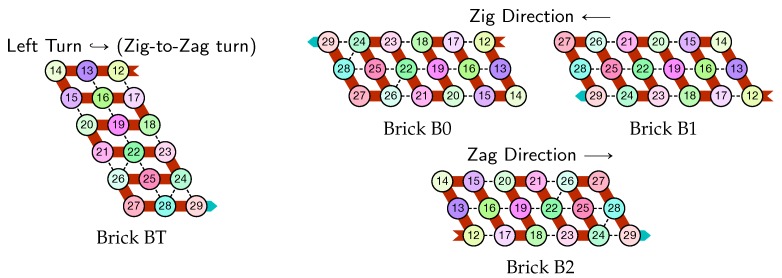
- Module *C*, Second Half-Adder (beads 30–41) 
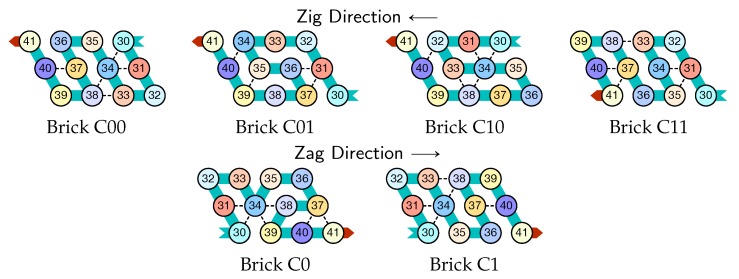
- Module *D*, Right-Turn module (beads 42–59) 
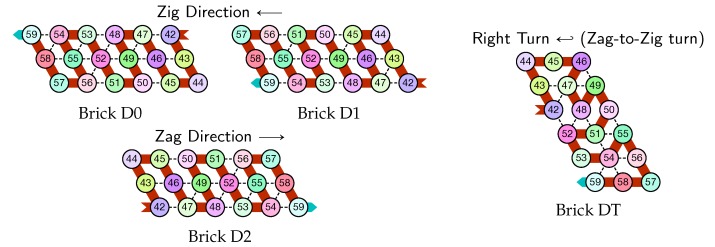

Note the similarities between the two half-adders *A* and *C* in their folding. Using two similar but different modules *A* and *C* allow to avoid interference and simplify the design.

**Figure 16:**
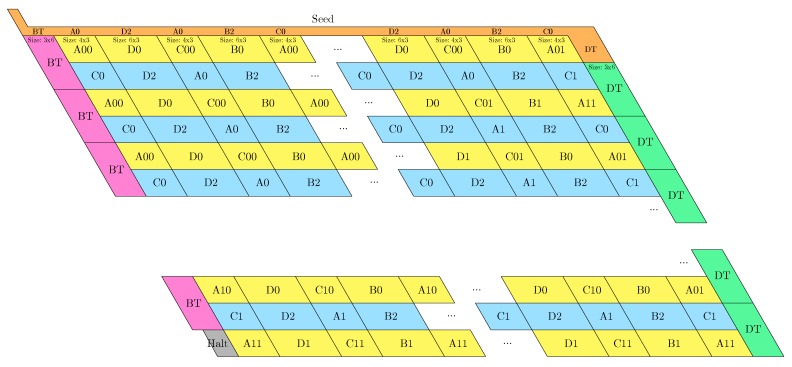
Triangular grid partition into regions populated with the proper bricks. Color coding: Seed-row in orange; Zig-rows (←) in yellow; Zag-rows (→) in blue; Left Turns (↪) in pink; and Right Turns (↩) in green.

**The final configuration.** In order to program the molecule, we have partitioned the triangular grid into *regions* that will be populated each by a module adopting one of the brick configurations above. The regions and the bricks populating them are displayed in Figure 16. With the exception of the seed region on top, the regions consist in parallelograms of size 4×3, 6×3 or 3×6 organized into $2\times 2^{2k+1}$ rows of height 3 and 4k+3 columns of widths 3 or 6. The regions are to be populated as follows to implement a (2k+1)-bits counter, as illustrated on Figure 16:

- The rightmost and leftmost columns have width 3:
  - The leftmost column consists in $2^{2k+1}$ (3×6)-regions all populated with the brick BT (Left Turn).
  - The rightmost column consists in $2^{2k+1}$ (3×6)-regions all populated with the brick DT (Right Turn).
- The 4k+1 inner columns consist in 4×3-parallelogram regions if odd and 6×3-parallelogram regions if even. The rows consist of an alternation of *Zig-* and *Zag-rows* to be read *from right to left* and *from left to right*, respectively. The rows 2i+1 and 2i+2 take care of reading *i* in binary from the row 2i above, incrementing it in row 2i+1, and writing i+1 in row 2i+2. In order to describe precisely the folding, let us denote by $i_{j}$ the *j*th lowest weight bits of i∈N when written in binary and by $\rho_{i}$ the position of the lowest-weight 0-bit of *i*: $\rho_{i}=\min\left\{j:i_{j}=0\right\}$. $\rho_{i}$ is the position up to which the carry propagates when one increments *i*: ρ=(1,2,1,4,1,2,1,4,1,2,1,8,1,2,…). Precisely, the region in the *p*th inner row, $1⩽p⩽2\cdot 2^{2k+1}-1$ and the *q*th inner column, 1⩽q⩽4k+1 is:
  - if p=2i+1 is odd and q=2r is even: a 3×6-parallelogram populated with Brick Kc, where:
    * K=B if *r* is even, and K=D if *r* is odd;
    * c=1 if $r/2<\rho_{i}$ (there still is carry to propagate), and c=0 if $r/2\geq \rho_{i}$ (there is no more carry to propagate).
  - if p=2i and q=2r are even: a 3×6-parallelogram populated with Brick B2 if *r* is even, or D2 if *r* is odd.
  - if p=2i is even and q=2r+1 is odd: a 4×3-parallelogram populated with Brick A$i_{r}$ if *r* is odd and C$i_{r}$ if *r* is even.
  - if p=2i+1 and q=2r+1 are odd: a 4×3-parallelogram populated with Brick $Ki_{r}c$ where:
    * K=A if *r* is even, and K=C if *r* is odd;
    * c=1 if $r/2⩽\rho_{i}$ (there still is carry to propagate), and c=0 if $r/2>\rho_{i}$ (there is no more carry to propagate).
- The seed row on top consists in the bottom row of the brick sequence BT,C0,(D2,A0,B2,C0${)}^{k}$,DT.
- The leftmost region of the last row is where the folding stops (counter capacity exceeded).

### 4.3. Input and Output Nascent Configurations

Recall that the inertial dynamics extends only the favored nascent configurations from the previous time step (i.e., the one that had the maximum number of bonds). We call this set of configurations, the *output nascent configurations* of the previous time step, or the *input nascent configurations* of the present time step. In this section, we list all the possible input/output nascent configurations according to our design.

**Figure 17:**
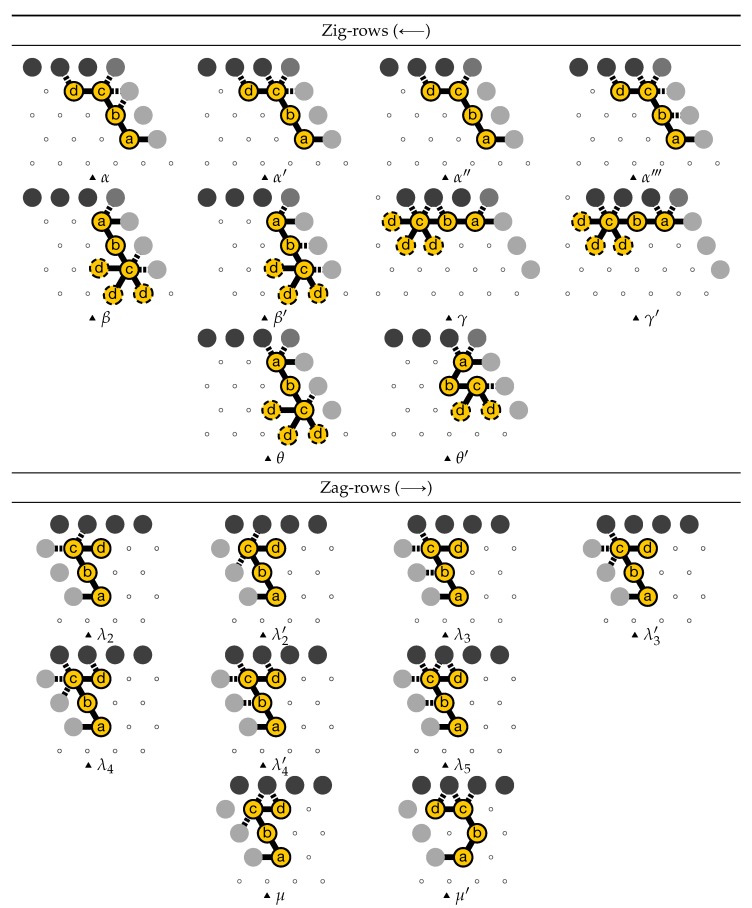
Notation for the various possible sets of output nascent configurations.

The lemmas in Supplementary Materials Section S.1.2 sum up the results and yield by a simple induction that the construction is correct. Let us denote $\alpha ,\alpha^{\prime },\alpha^{\prime \prime },\alpha^{\prime \prime \prime },\beta ,\beta^{\prime },\gamma ,\gamma^{\prime },\theta ,\theta^{\prime },\lambda_{2},\lambda_{2}^{\prime },\lambda_{3},\lambda_{3}^{\prime },\lambda_{4},\lambda_{4}^{\prime },\lambda_{5},\mu ,\mu^{\prime }$ all the possible sets of *output nascent configurations* for all bricks as illustrated in Figure 17. Note  that output nascent configurations $\beta ,\beta^{\prime },\gamma ,\gamma^{\prime },\theta ,\theta^{\prime }$ do not represent a single configuration but a set of configurations as the position of the last bead *d* is not fixed.

**Figure 18:**
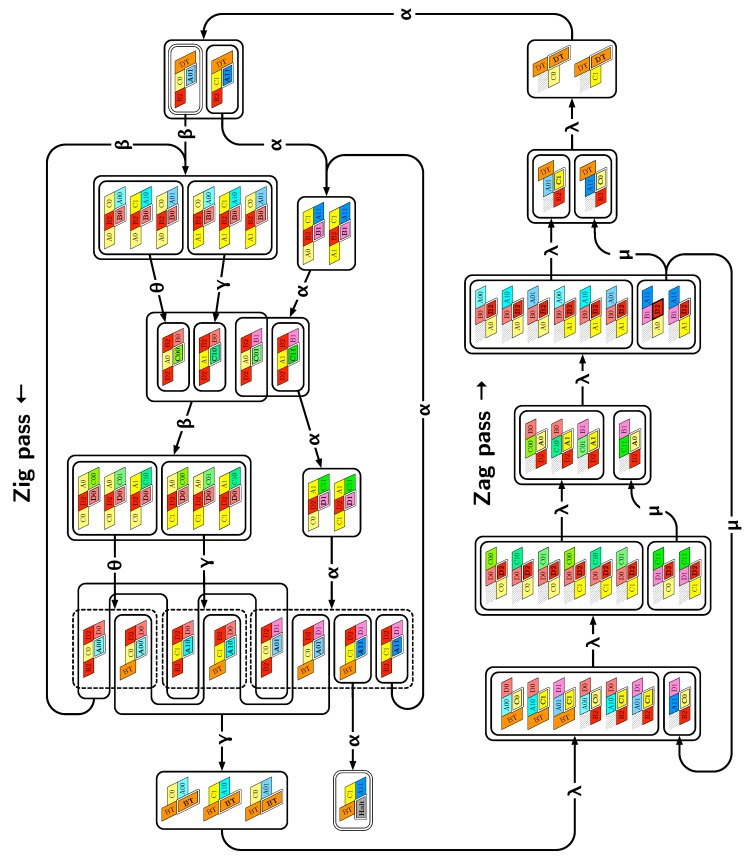
The brick automaton illustrating how Lemmas 1–10 (Supplementary Materials) work together to prove Theorem 1 by induction.

Figure 18 illustrates the results proven in the following lemmas and demonstrates that the induction is correct and proves that the bricks fold one after the other so that the molecule folds indeed into the claimed final configuration presented in Figure 16.

**Proof of Correctness.** The proof of Theorem 1 proceeds by induction: it is enough to show that each module folds into the expected brick in each region. The folding of each module depends on its environment, i.e., on the bricks already folded nearby and on the current minimum energy configurations output by the previous step. First, Supplementary Materials Section S.1.1 enumerates all the possible environments for each module. Then, Supplementary Materials Section S.1.2 shows that if each module folds as expected, then Theorem 1 is correct. Finally, Supplementary Materials Section S.2 provides all the folding certificates proving that each module folds as expected in every possible environment which concludes the proof of correctness of our 60-beads long periodic oritatami system implementing a binary counter using inertial dynamics.

## 5. Rule Design Is NP-Hard and FPT

Our second main result concerns the design of a rule for achieving a set of given foldings depending on the environment.

**The rule design problem (RDP)** consists in the following:

**Input:**  Two *disjoint* sets of bead types *B* and {1,…,n}, a delay δ, *k* seed configurations $\sigma^{1},\ldots ,\sigma^{k}$ of sequences $s^{1},\ldots ,s^{k}\in B^{*}$ (with possibly different lengths) and *k* target configurations $c^{1},\ldots ,c^{k}$ of sequence p=〈1,…,n〉 of length *n*, and a dynamics D∈{O,I}.
**Output:**  A rule 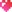 $\subseteq {\left(B⊔\left\{1,\ldots ,n\right\}\right)}^{2}$ such that for all i=1..k, the Oritatami system O=(p, 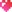, δ) folds deterministically into the configuration $\sigma^{i}c^{i}$ from the seed configuration $\sigma^{i}$ according to dynamics D, i.e., such that: $D_{s^{i}p}^{n-\delta +1}\left({\left(\sigma^{i}\right)}^{\;▹\left(\delta -1\right)}\right)=\left\{\sigma^{i}c^{i}\right\}$ for all i=1..k.

### 5.1. NP-Completeness

We begin by showing that the rule design problem is NP-complete:

**Theorem** **4.** *For any positive delay and transcript length, the rule design problem is NP-complete.*

**Proof.**  We reduce from 3-SAT with *q* variables $x_{1},\ldots ,x_{q}$ and *m* 3-clauses $C_{1},\ldots ,C_{m}$ to the rule design problem by designing 3+2q bead types $B=\left\{1,\ldots ,n\right\}⊔\left\{r,z,x_{1},\bar{x_{1}},\ldots ,x_{q},\bar{x_{q}}\right\}$, the (fixed length) transcript p=〈1,…,n〉, and q+m pairs of seed-target configurations $\left(\sigma^{1},c^{1}\right),\ldots ,\left(\sigma^{q},c^{q}\right),\left(\sigma^{\prime 1},c^{\prime 1}\right),\ldots ,\left(\sigma^{\prime m},c^{\prime m}\right)$, such that $\phi =C_{1}\wedge \cdots \wedge C_{m}$ is satisfiable if and only if there is a rule such that *p* folds into $c^{i}$ starting from $\sigma^{i}$, and into $c^{\prime i}$ from $\sigma^{\prime i}$. It will follow that finding a rule is NP-hard (in the number of pairs of seed-target configuration pairs).

**Figure 19:**
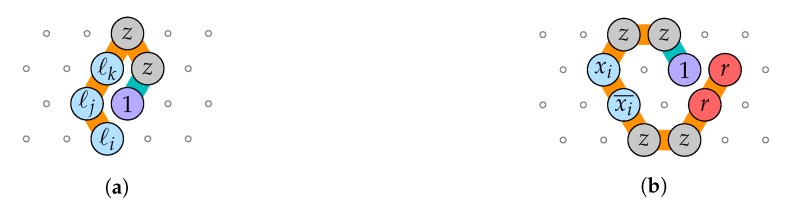
The polynomial-time reduction for 3-SAT to the rule design problem for delay δ=1. The seed configurations are linked in orange, and the target configurations are linked in turquoise. (**a**) The seed-target configuration pair for clause $\ell_{i}\vee \ell_{j}\vee \ell_{k}$: it is deterministically foldable iff 1 is attracted by at least one of $\ell_{i},\ell_{j},\ell_{k}$; (**b**) The seed-target configuration pair for variable $x_{i}$: it is deterministically foldable iff 1 is attracted by *r* and 1 is attracted by at most one of *z*, $x_{i}$ and $\bar{x_{i}}$.

Figure 19 shows the seed-target configuration pairs for delay δ=1. Here, p=〈1〉 of length n=1. This reduction verifies the following property: there is a rule that folds every one of them deterministically iff there is a rule such that 1 is attracted by at least one literal of each clause, and at most one of each variable or its negation. It follows that such a rule exists if and only if ϕ is satisfiable (by setting to true every literal to which 1 is attracted).

**Figure 20:**
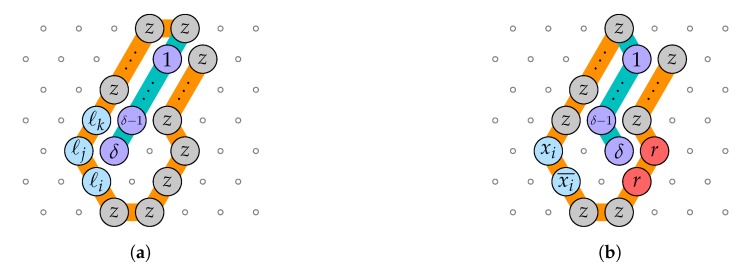
The reduction for 3-SAT to the rule design problem for delay δ≥2. (**a**) The seed-target configuration pair for clause $\ell_{i}\vee \ell_{j}\vee \ell_{k}$: it is deterministically foldable iff δ is attracted by at least one of $\ell_{i},\ell_{j},\ell_{k}$. (**b**) The seed-target configuration pair for variable $x_{i}$: it is deterministically foldable iff δ is attracted by *r* and δ is attracted by at most one of $x_{i}$ and $\bar{x_{i}}$.

Finally, extending this proof to a larger delay time δ≥2 can be done as shown in Figure 20 by setting p=〈1,…,δ〉 (of length n=δ) and augmenting in the seed configurations with a tunnel of length δ−1 that funnels the δ−1 first beads of the transcript *p*. This reduces to the delay 1 case. □

### 5.2. An Efficient Algorithm in Practice

As can be observed in the proof of Theorem 4, the NP-hardness of the Rule Design Problem depends on the number of desired configurations (*k*) and *not on the length of the transcript (n)*. As Theorem 3 will show next, the problem is indeed linear-time in the length *n* of the transcript when the number of desired configuration is a constant, and exponential-time only in *k* and δ. Such a problem is said *fixed parameter tractable (FPT)* as it can be solved in polynomial time when some parameters (here *k* and δ) are small constants. As one usually onlyfixed parameter tractable (FPT desires a small number of different configurations for every given part of the transcript, the algorithm described below is very efficient in practice and allowed us to solve various key parts of our design in spite of its NP-hardness in general!

**Theorem** **5.** *thm:fpt The rule design problem (RDP) with k seed-target configurations, each of length n, and delay time δ is fixed-parameter tractable, as it can be solved in time and space complexity $O_{k,\delta }\left(n\right)$. More precisely. Algorithm 1 solves RDP for the oblivious dynamics O in time $O(n\cdot 5^{\delta }2^{3k(\delta^{3}+\delta^{2}+4\delta +1)})$ time and uses $O(n\cdot \delta^{2}2^{3k(\delta^{3}+\delta^{2}+4\delta +1)})$ space; and Algorithm 2 solves RDP for the inertial dynamics I in $O(n\cdot 5^{\delta }2^{k5^{\delta -1}+3k\left(\delta^{3}+\delta^{2}+4\delta +1\right)})$ time and uses $O(n\cdot \delta^{2}5^{\delta }2^{k5^{\delta -1}+3k\left(\delta^{3}+\delta^{2}+4\delta +1\right)})$ space.*

The bounds given on the memory use seem to indicate that the algorithm might be impractical even if its time complexity is linear in the length of the transcript. However, we implemented lazily (i.e., allocating memory only when needed) and then, its memory usage remains modest because the number of output nascent configurations remains small. We have used this procedure successfully to solve key parts of our design. Indeed, note that in our design, there are only very few input configurations (most of the time just one) which makes the potentially heavy extension step very fast in practice, at least for this design.

**Algorithm 1** Rule Design Problem FPT Algorithm—Oblivious dynamics
1:**function**ExtendOblivious(*i*, a partial rule *R*)2:    ▹*This procedure computes all the possible extensions of rule R s.t. bead $p_{i}$ is placed at its desired location in all k target configurations*3:    Let Σ=∅4:    Let N be the bead types reachable by the (i+δ−1)-th beads of the transcript *p* in any of the δ-elongations of the (i−1)-prefixes of the target configurations $\sigma^{1}c_{1..i-1}^{1},\ldots ,\sigma^{k}c_{1..i-1}^{k}$5:    **for all** partial rule $\rho :\left\{p_{i+\delta -1}\right\}\times N\to \left\{\mathrm{true},\mathrm{false}\right\}$ **do**6:        Let $R^{\prime }=R\cup \rho$7:        **try**8:           **for** j=1..k **do**9:               let $S_{j}={\left(\sigma^{j}c_{1..i-1}^{j}\right)}^{\;▹\left(\delta -1\right)}$10:               let $S_{j}^{\prime }=O_{R^{\prime }}\left(S_{j}\right)={⋃}_{\gamma \in \sigma_{j}c_{1..i-1}^{j}}\;\;\;\underset{c\in \gamma^{▹\gamma }}{\arg\;\min}E_{R^{\prime }}\left(c\right)$11:               **if** $S_{j}^{\prime }=\emptyset$ or $\exists c^{\prime }\in S_{j}^{\prime }$ s.t. $c_{i}^{\prime }\neq c_{i}^{j}$ **then**12:                   **throw** *rejectRule*     ▹ The rule $R^{\prime }$ fails to place the bead $p_{i}$ at its desired position13:           $\Sigma :=\Sigma \cup \{R^{\prime }\}$                              ▹ Add $R^{\prime }$ to Σ14:        **catch** *rejectRule*: **continue**                         ▹ Do not add $R^{\prime }$ to Σ15:    **return** Σ16:**function**ProjectOblivious(*i*, Set R of partial rules *R*))17:    ▹*This procedure retains only one representative partial rule for every possible subrule of the last δ−1 nascent beads*18:    Let Π:=∅19:    Let N be the bead types reachable by the δ−1 nascent beads $p_{i+1},\ldots ,p_{i+\delta -1}$ in any of the (δ−1)-elongations of the *i*-prefixes of the target configurations $\sigma^{1}c_{1..i}^{1},\ldots ,\sigma^{k}c_{1..i}^{k}$20:    **for all** partial rule $\rho :\left\{p_{i+1},\ldots ,p_{i+\delta -1}\right\}\times N\to \left\{\mathrm{true},\mathrm{false}\right\}$ **do**21:        Let $R_{\rho }$ be the subset of all partial rules *R* in R matching with ρ.22:        **if** $R_{\rho }\neq \emptyset$ **then**23:           Pick one rule $R\in R_{\rho }$ to be the representative of set $R_{\rho }$ and add it to Π: Π:=Π∪{R}24:    **return** Π25:**function**FindRuleOblivious( )26:    Let N be the bead types reachable by the δ−1 nascent beads $p_{1},\ldots ,p_{\delta -1}$ in any of the (δ−1)-elongations of the *k* seed configurations $\sigma^{1},\ldots ,\sigma^{k}$27:    Initialize R as the set of all partial rules $R:\left\{p_{1},\ldots ,p_{\delta -1}\right\}\times N\to \left\{\mathrm{true},\mathrm{false}\right\}$28:    **for** i=1..n−δ+1**do**               ▹ Extend the rule to place correctly the bead $p_{i}$29:        Let Σ=∅30:        **for all** partial rule R∈R **do**31:           Σ:=Σ∪ ExtendOblivious(*i, R*)32:        Update R := ProjectOblivious(*i*, Σ)33:    **for all** R∈R and j=1..k**do**          ▹ Check the positions of the last δ−1 nascent beads34:        **if** $O_{R}\left(\sigma^{j}{\left(c^{j}\right)}^{◃1}\right)\neq \left\{\sigma^{j}c^{j}\right\}$ **then**35:           Remove *R* from R: R:=R∖{R}36:    **if** R≠∅ **then**37:        Pick a rule R∈R and **return***R*38:    **else**39:        **return** “There is no rule building the *k* target configurations together obliviously”


**Algorithm 2** Rule Design Problem FPT Algorithm—Inertial dynamics
1:**function**ExtendInertial(*i*, a partial rule *R*, the *k* sets of nascent configurations $S^{1},\ldots ,S^{k}$ up to bead $p_{i+\delta -2}$ favored by *R* in each of the *k* target environments)2:    ▹*This procedure computes all the possible extensions of rule R s.t. bead $p_{i}$ is placed at its desired location in all k target configurations*3:    Let Σ=∅4:    Let N be the bead types reachable by the (i+δ−1)-th bead of the transcript *p* in any of the 1-elongations of the favored nascent configurations in $S^{1},\ldots ,S^{k}$5:    **for all** partial rule $\rho :\left\{p_{i+\delta -1}\right\}\times N\to \left\{\mathrm{true},\mathrm{false}\right\}$ **do**6:        Let $R^{\prime }=R\cup \rho$7:        **try**8:           **for** j=1..k **do**9:               let ${S^{\prime }}^{j}=I_{R^{\prime }}\left(S^{j}\right)={⋃}_{\gamma \in {S^{j}}^{◃\left(\delta -1\right)}}\;\;\;\underset{c\in \gamma^{▹\gamma }⋂{S^{j}}^{▹1}}{\arg\;\min}E_{R^{\prime }}\left(c\right)$10:               **if** ${S^{\prime }}^{j}=\emptyset$ or $\exists c^{\prime }\in {S^{\prime }}^{j}$ s.t. $c_{i}^{\prime }\neq c_{i}^{j}$ **then**11:                   **throw** *rejectRule*     ▹ The rule $R^{\prime }$ fails to place the bead $p_{i}$ at its desired position12:           $\Sigma :=\Sigma \cup \{\langle R^{\prime },{S^{\prime }}^{1},\ldots ,{S^{\prime }}^{k}\rangle \}$       ▹ Add $R^{\prime }$ and its favored nascent configurations to Σ13:        **catch** *rejectRule*: **continue**        ▹ Do not add $R^{\prime }$ to Σ14:    **return** Σ15:**function**ProjectInertial(*i*, Set S of tuples 〈partial rule *R*, the *k* sets of nascent configurations $S^{1},\ldots ,S^{k}$ up to bead $p_{i+\delta -1}$ favored by *R* in each of the *k* target environments〉)16:    ▹*This procedure retains only one representative partial rule for every possible subrule and resulting favored nascent configurations of the last δ−1 nascent beads in every environments*17:    Let Π:=∅18:    Let N be the bead types reachable by the δ−1 nascent beads $p_{i+1},\ldots ,p_{i+\delta -1}$ in any of the δ−1 elongations of the partial target configurations $\sigma^{1}c_{1..i}^{1},\ldots ,\sigma^{k}c_{1..i}^{k}$19:    **for all** partial rule $\rho :\left\{p_{i+1},\ldots ,p_{i+\delta -1}\right\}\times N\to \left\{\mathrm{true},\mathrm{false}\right\}$**and all** subsets $\Gamma^{1},\ldots ,\Gamma^{k}$ of (δ−1)-elongations of the partial target configurations $\sigma^{1}c_{1..i}^{1},\ldots ,\sigma^{k}c_{1..i}^{k}$ **do**20:        Let $R_{\rho ,\Gamma }$ be the subset of all partial rules *R* in S which match with ρ and whose favored nascent configurations are the sets $\Gamma^{1},\ldots ,\Gamma^{k}$, i.e.:
$$\mathrm{let}\;R_{\rho ,\Gamma }=\left\{R:R\;\mathrm{matches}\;\mathrm{with}\;\rho \;\mathrm{and}\;\langle R,\Gamma^{1},\ldots ,\Gamma^{k}\rangle \in S\right\}$$21:        **if** $R_{\rho ,\Gamma }\neq \emptyset$ **then**22:           Pick one rule $R\in R_{\rho ,\Gamma }$ to be the representative of set $R_{\rho ,\Gamma }$ and add it to Π:
$$\Pi :=\Pi \cup \{\langle R,\Gamma^{1},\ldots ,\Gamma^{k}\rangle \}$$23:    **return** Π24:**function**FindRuleInertial( )25:    Let N be the bead types reachable by the δ−1 nascent beads $p_{1},\ldots ,p_{\delta -1}$ in any of the (δ−1)-elongations of the *k* seed configurations $\sigma^{1},\ldots ,\sigma^{k}$26:    Initialize S as the set of all tuples $\langle R,{\sigma^{1}}^{\;▹\delta -1},\ldots ,{\sigma^{k}}^{\;▹\delta -1}\rangle$ for all partial rules $R:\left\{p_{1},\ldots ,p_{\delta -1}\right\}\times N\to \left\{\mathrm{true},\mathrm{false}\right\}$27:    **for** i=1..n−δ+1 **do**    ▹ Extend the rule to place correctly the bead $p_{i}$28:        Let Σ=∅29:        **for all** tuple $\langle R,S^{1},\ldots ,S^{k}\rangle \in S$ **do**30:           Σ:=Σ∪ ExtendInertial(*i, R, S*^1^, …, *S^k^*)31:        Update S := ProjectInertial(*i*, Σ)32:    **if** $\exists \;\langle R,\left\{\sigma^{1}c^{1}\right\},\ldots ,\left\{\sigma^{k}c^{k}\right\}\rangle \in S$ **then**  ▹ Check the positions of the last δ−1 nascent beads33:        **return** *R*34:    **else**35:        **return** “There is no rule building the *k* target configurations together inertially”

**Proof** **(Proof sketch (complete proof in Supplementary Materials Section S.3))**  The key is that every step of the folding is computed locally in a fixed and known environment: at each step *i*, the δ beads to be folded look for their best positions by interacting with beads with fixed and known positions within a radius δ+1. It follows that one can compute the set of all suitable subrules, considering these $O(\delta^{2})$ bead types only (i.e., that place the i−δ+1-th bead of the molecule at the correct position). Oblivious O and inertial I dynamics differ as I only consider input nascent configurations which are output by the previous step, whereas O does not use any information from the previous step. This implies that one need to remember some information to connect one step to the next in I, whereas O needs no memory at all.

Formally, a *subrule* $R:B^{2}\to \left\{\mathrm{true},\mathrm{false},⊥\right\}$ is a symmetric function that states for each pair of beads if they attract each other (true) or not (false), or if this is undefined (⊥). We denote by $\mathrm{dom}R=\{\left(a,b\right)\in B^{2}:R\left(a,b\right)\neq ⊥\}$ the *domain* of *R*. Two subrules $R_{1}$ and $R_{2}$ are *compatible*, denoted by $R_{1}∼R_{2}$ if they agree for every pair where they are both defined, i.e., if for all $a,b\in \mathrm{dom}R_{1}\cap \mathrm{dom}R_{2}$, $R_{1}\left(a,b\right)=R_{2}\left(a,b\right)$. We say that $R_{1}$ *matches with* $R_{2}$ if for all $\left(a,b\right)\in \mathrm{dom}R_{2}$, $R_{1}\left(a,b\right)=R_{2}\left(a,b\right)$. If $R_{1}∼R_{2}$, we denote by $R_{1}\cup R_{2}$ the subrule obtained by *merging* $R_{1}$ and $R_{2}$, i.e. defined by $R_{1}\cup R_{2}\;\left(a,b\right)=R_{1}\left(a,b\right)$ if $\left(a,b\right)\in \mathrm{dom}R_{1}$, and $=R_{2}\left(a,b\right)$ otherwise.

For all 0⩽1⩽|c|, we denote by $c_{1..i}$ the prefix of length *i* of a configuration *c*. Algorithm 1 solves RDP for the oblivious dynamics in time linear in the length of the sequence as follows. It incrementally constructs a set of rules that place each bead at its desired position in each of the *k* environments. It proceeds by maintaining a set R of candidate subrules that place correctly the first *i* beads in every of the *k* environments. At the *i*-th step, the main procedure FindRuleOblivious() first extends each candidate subrule *R* in R by calling procedure ExtendOblivious() which scans all the possible attraction rule extensions of *R* for the new nascent bead (the (i+δ−1)-th) with the bead types it can reach in each of the *k* configurations, and retains only the ones that place the *i*-th bead at its correct position for all *k* configurations. Note that this extension of the rule does not change the positioning of the (i−1)th first beads since the (i+δ−1)-th bead is not yet produced when they are placed. Now, in order to keep the processing time constant for each bead, main procedure FindRuleOblivious() calls procedure ProjectOblivious() which retains only one representative of each subset of rules that define the same attractions for the δ−1 nascent beads (indexed from i+1 to i+δ−1), i.e., for the only bead types for which the rule matters in order to determine the positions of the upcoming beads. Once the n−δ-th bead is placed, the main procedure concludes by checking that the surviving rules place the last δ−1 beads at their desired positions.

Algorithm 2 works similarly for the inertial dynamics I. In fact, we just extend the subrule technics by testing subrules for each possible input and corresponding output nascent configurations set, for each seed-target configurations pair and each time step. □

### 5.3. Comparison with Related Works

A variant of the rule design problem was studied by Ota and Seki [43] with one single seed/target configurations pair and where the transcript can be arbitrary (any bead type can be used, including repetition and one from the seed). In the oblivious dynamics, they fully characterize for all possible combinations of delay δ and arity α for which this variant either can be solved in a polynomial time or is NP-hard. This problem remains as hard even if the transcript is required to be periodic.

The uniqueness of the bead types used in the transcript is thus crucial for the FPT algorithm to exist. Thus, it is not clear whether their variant admits an FPT algorithm or not. Note that other algorithms were proposed, such as the heuristic rule set optimization algorithm by Han and Kim [45].

## 6. Perspectives

The purpose of our new model is not to be entirely accurate with respect to phenomena observed in nature, but instead to start developing an intuition about the *kind of problem* that need to be solved in order to engineer RNA shapes, and later, even proteins.

In the future, a number of extensions of this model seem natural. In particular, extending it with a more realistic notion of *thermodynamics and molecular agitation*. Using existing works in molecular dynamics [47], would allow to explore stochastic optimization processes. It would be of highest interest if one could come up with a stochastic extension of this model that is still able of Turing-complete computation as was shown in [42] for the present model.

## Supplementary Materials

The following are available online at https://www.mdpi.com/1422-0067/20/9/2259/s1.
