# Supplementary material for: Oritatami: A Computational Model for Molecular Co-Transcriptional Folding
Source: Int J Mol Sci. 2019 May 7;20(9):2259. doi: 10.3390/ijms20092259 (PMC6539498; doi:10.3390/ijms20092259)
Supplement: Supplementary file 1 [file ijms-20-02259-s001.pdf]

International Journal of
Molecular Sciences

MDPI

Article

Oritatami: A Computational Model for Molecular Co-Transcriptional Folding

Cody Geary [1], Pierre-Étienne Meunier [2], Nicolas Schabanel [3,*] and Shinnosuke Seki [4]

1 Computer Science Computation and Neural Systems Bioengineering Caltech, MS 136-93, Moore Building, Pasadena, CA 91125, USA; codyge@gmail.com
2 Computer Science Dept, Hamilton Institute, Maynooth University, Co. Kildare, Ireland; pierre-etienne.meunier@mu.ie
3 CNRS, École normale supérieure de Lyon (LIP), CEDEX 07, 69364 Lyon, France
4 Computer and Network Engineering Dept, University of Electro-Communications, 1-5-1, Chofugaoka, Chofu, Tokyo 1828585, Japan; s.seki@uec.ac.jp
* Correspondence: nicolas.schabanel@ens-lyon.fr

Received: 15 April 2019; Accepted: 30 April 2019; Published: 7 May 2019

check for updates

Supplementary Materials:

S.1. Correctness of the Folding

This section contains the missing part of the proof of correctness of the counter Oritatami system in Section 4 proving our main Theorem 1.

S.1.1. Enumerating All the Possible Environments

The proof of Theorem 1 proceeds by induction: it is enough to show that each module folds into the expected brick in each region. The folding of each module depends on its environment, i.e., on the bricks already folded nearby and on the current minimum energy configurations output by the previous step. Recall that the Zig-rows (the one that begin and end with an A-brick before the turns) fold from right to left and that the Zag-rows (the one that begin and end with a C-brick before the turns) fold from left to right. Let us now review the possible environments for each module and the bricks they are expected to fold into in each possible environment (for each of them we refer to the figure providing with the corresponding folding tree certificate):

- Module A, First Half-Adder:
  - In Zig-rows (←):
    - *when Module A folds in the rightmost column of a Zig-row,* its environment is *to the right:* always DT; and *above:* either B2,C0 or B2,C1. For each of these two environments, Module A is expected to fold into the following brick:

		Bricks above: B2,C0	Bricks above: B2,C1
Brick to the right:	DT	A01 (Figure S.3)	A11 (Figure S.4)

    - *when Module A folds in an inner column of a Zig-row,* its environment is *to the right:* either D0 or D1; *above:* either B2,C0 or B2,C1. For each of these four environments, Module A is expected to fold into the following brick:

		Bricks above: B2,C0	Bricks above: B2,C1
Brick to the right:	D0	A00 (Figure S.5)	A10 (Figure S.7)
	D1	A01 (Figure S.6)	A11 (Figure S.8)

    * *when Module A folds in the leftmost column of a Zig-row,* its environment is *to the right:* either D0 or D1; and *above:* either BT,C0 or BT,C1. For each of these four environments, Module A is expected to fold into the following brick:

		Bricks above: BT,C0	BT,C1
Brick to the right:	D0	A00 (Figure S.9)	A10 (Figure S.11)
	D1	A01 (Figure S.10)	A11 (Figure S.12)

  - In Zag-rows (→): Module A folds in a 4 × 3-region surrounded *to the left:* always by D2; and *above:* by either C00,B0 or C01,B1 or C10,B0 or C11,B1. For each of these four environments, Module A is expected to fold into the following brick:

		Bricks above: C00,B0	C01,B1	C10,B0	C11,B1
Brick to the left:	D2	A0 (Figure S.13)	A1 (Figure S.14)	A1 (Figure S.15)	A0 (Figure S.16)

- Module B, Left Turn:
  - In the inner columns of a Zig-row (←): Module B folds in a 6 × 3-region surrounded *to the right:* by either A00, A01, A10, or A11 and *above:* by A0,B2 or A1,B2. For each of these height environments, Module B is expected to fold into the following brick:

		Bricks above: A0,B2	A1,B2
Brick to the right:	A00	B0 (Figure S.17)	B0 (Figure S.18)
	A01	B0 (Figure S.19)	B0 (Figure S.20)
	A10	B0 (Figure S.21)	B0 (Figure S.22)
	A11	B1 (Figure S.23)	B1 (Figure S.24)

  - In the leftmost column of a Zig-row (←): Module B folds in a 3 × 6-region surrounded *to the right:* by either A00, A01, A10, or A11 and *above:* by BT. For each of these four environments, Module B is expected to fold into the following brick:

		Bricks above: BT
Brick to the right:	A00	BT (Figure S.25)
	A01	BT (Figure S.26)
	A10	BT (Figure S.27)
	A11	Halting non-deterministic configuration (Figure S.28)

  - In the columns of a Zag-row (→): Module B folds in a 6 × 2-region surrounded *to the left:* by either A0 or A1 and *above:* by B0,A00, or B0,A01, or B0,A10 or B1,A11. For each of these height environments, Module B is expected to fold into the brick B2:

		Bricks above: B0,A00	B0,A01	B0,A10	B1,A11
Brick to the left:	A0	B2 (Figure S.29)	B2 (Figure S.30)	B2 (Figure S.31)	B2 (Figure S.32)
	A1	B2 (Figure S.33)	B2 (Figure S.34)	B2 (Figure S.35)	B2 (Figure S.36)

- Module C, Second Half-Adder:
  - In Zig-rows (←): Module C folds in a 4 × 3-region surrounded *to the right:* either by B0 or B1 ; and *above:* by either D2,A0 or D2,A1. For each of these four environments, Module C is expected to fold into the following brick:

		Bricks above: D2,A0	D2,A1
Brick to the right:	B0	C00 (Figure S.37)	C10 (Figure S.38)
	B1	C01 (Figure S.39)	C11 (Figure S.40)

  - In Zag-rows (→):

∗ *when Module C folds in the leftmost column of a Zag-row,* its environment is *to the left:* always BT; and *above:* either A00,D0 or A01,D1 or A10,D0 or A11,D1. For each of these four environments, Module C is expected to fold into the following brick:

		Bricks above: A00,D0	A01,D1	A10,D0	A11,D1
Brick to the left:	BT	C0 (Figure S.41)	C1 (Figure S.42)	C1 (Figure S.43)	Never occurs

∗ *when Module C folds in an inner column of a Zag-row,* its environment is *to the left:* always B2; and *above:* either A00,D0 or A01,D1 or A10,D0 or A11,D1. For each of these four environments, Module C is expected to fold into the following brick:

		Bricks above: A00,D0	A01,D1	A10,D0	A11,D1
Brick to the left:	B2	C0 (Figure S.44)	C1 (Figure S.45)	C1 (Figure S.46)	C0 (Figure S.47)

∗ *when Module C folds in the rightmost column of a Zag-row,* its environment is *to the right:* always B2; *above:* either A01,DT or A11,DT. For each of these two environments, Module C is expected to fold into the following brick:

		Bricks above: A01,DT	A11,DT
Brick to the right:	B2	C1 (Figure S.48)	C0 (Figure S.49)

- Module D, Right Turn:

  – In a column of a Zig-row (←): Module D folds in a 6×3-region surrounded *to the right:* by either C00, C01, C10, or C11 and *above:* by C0,D2 or C1,D2. For each of these height environments, Module D is expected to fold into the following brick:

		Bricks above: C0,D2	C1,D2
Brick to the right:	C00	D0 (Figure S.50)	D0 (Figure S.51)
	C01	D0 (Figure S.52)	D0 (Figure S.53)
	C10	D0 (Figure S.54)	D0 (Figure S.55)
	C11	D1 (Figure S.56)	D1 (Figure S.57)

  – In an inner column of a Zag-row (→): Module D folds in a 6×3-region surrounded *to the left:* by either C0 or C1 and *above:* by D0,C00 or D0,C01 or D0,C10 or D1,C11. For each of these height environments, Module D is expected to fold into the brick D2:

		Bricks above: D0,C00	D0,C01	D0,C10	D1,C11
Brick to the left:	C0	D2 (Figure S.58)	D2 (Figure S.59)	D2 (Figure S.60)	D2 (Figure S.61)
	C1	D2 (Figure S.62)	D2 (Figure S.63)	D2 (Figure S.64)	D2 (Figure S.65)

  – In the rightmost column of a Zag-row (→): Module D folds in a 3×6-region surrounded *to the left:* by either C0 or C1 and *above:* by DT. For each of these two environments, Module D is expected to fold into the brick DT:

		Bricks above: DT
Brick to the left:	C0	DT (Figure S.66)
	C1	DT (Figure S.67)

Figure S.1 presents the positions of all the possible bricks in all the possible environments (written in white) in the folding of a 5-bits counter that contains all of them.

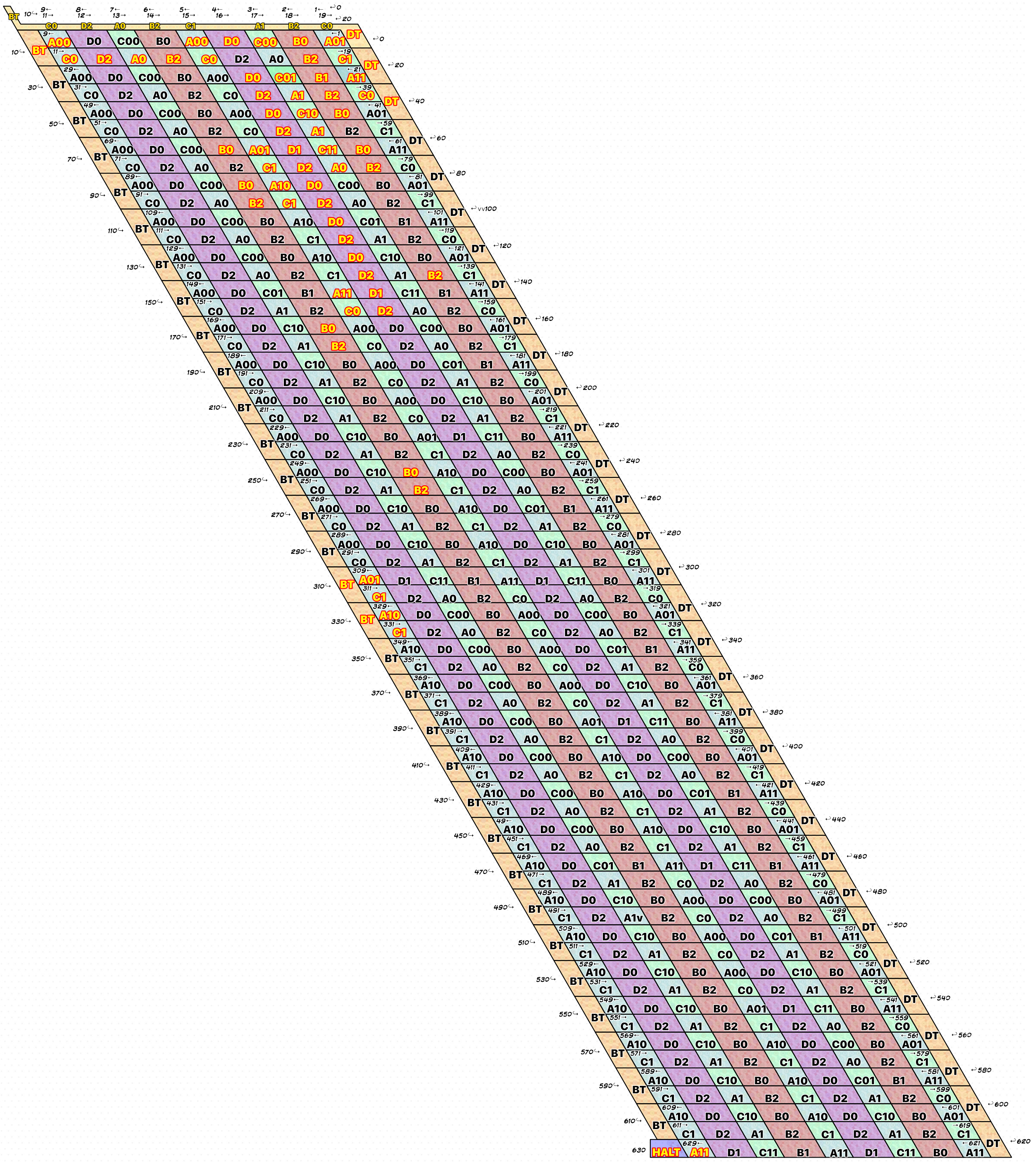

Figure S.1. The positions in the folding of the 5-bits counter of all the bricks analyzed in the following.

S.1.2. Proof of the Correctness of the Local Folding

S.1.2.1. Zig Rows

The following lemmas prove that the molecule folds as claimed in the zig-rows.

Lemma 1 (Modules A and C-inner column-Zig row-no carry propagation)**.** *When folded in an inner column of a Zig-row from the output configurations of the previous brick Dc (resp. Bc) and under a brick Cx (resp. Ax) s.t.* $x + c \leqslant 1$ *(i.e., no carry propagation), module A (resp. C) folds into the brick* $A(x + c)$ *(resp.* $C(x + c)$*) with output configurations* β*.*

Proof. This property is proved by the folding certificates in Figures:

- Module A: Figures S.3 and S.5– S.7.
- Module C: Figures S.37–S.39. □

Lemma 2 (Module A-leftmost column-no carry propagation)**.** *When folded in the leftmost column of a Zig-row from the output configurations of the previous brick Dc and under a brick Cx s.t.* $x + c \leqslant 1$ *(i.e., no carry propagation), module A folds into the brick* $A(x + c)$ *with output configuration* γ*.*

Proof. The folding certificates in Figures S.9–S.11 prove this property. □

Lemma 3 (Modules A and C-Zig row (all columns)-with carry propagation)**.** *When folded in a Zig-row from the output configurations of the previous brick D1 (resp. B1) and under a brick C1 (resp. A1), module A (resp. C) folds into the brick A11 (resp. C11) with output configuration* α' *(resp.* α'''*).*

Proof. This property is proved by the folding certificates in Figures:

- Module A: Figures S.4, S.8 and S.12.
- Module C: Figure S.40. □

Lemma 4 (Modules B and D-inner column-Zig row-no carry propagation)**.** *When folded in an inner column of a Zig-row from the output configurations of the previous brick Axc (resp. Cxc) and under bricks CzD2 (resp. AzB2) s.t.* $x + c \leqslant 1$*, module D (resp. B) folds into the brick D0 (resp. B0) with output configurations:* $\theta \cup \theta'$ *if* $z = 0$*; and* γ' *if* $z = 1$*.*

Proof. This property is proved by the folding certificates in Figures:

- Module D: Figures S.50, S.52 and S.54 for $z = 0$; and Figures S.51, S.53 and S.55 for $z = 1$.
- Module B: Figures S.17, S.19 and S.21 for $z = 0$; Figures S.18, S.20 and S.22 for $z = 1$. □

Lemma 5 (Modules B and D-inner column-Zig row-with carry propagation)**.** *When folded in an inner column of a Zig-row from the output configurations of the previous brick A11 (resp. C11) and under a brick D2 (resp. B2), module D (resp. B) folds into the brick D1 (resp. B1) with output configuration* α''*.*

Proof. This property is proved by the folding certificates in Figures:

- Module D: Figures S.56 and S.57.
- Module B: Figures S.23 and S.24. □

Lemma 6 (Modules B-leftmost column-Zig row-with no carry propagation)**.** *When folded in the leftmost column of a Zig-row from the output configurations of the previous brick Axc with* $x + c \leqslant 1$ *and under a brick BT, module B folds into the brick BT with output configuration* λ'_2*.*

Proof. This property is proved by the folding certificates in Figures S.25–S.27. □

Lemma 7 (Modules B-leftmost column-Zig row-with carry propagation)**.** *When folded in the leftmost column of a Zig-row from the output configurations of the previous brick A11 and under a brick BT, module B folds into the halting non-deterministic configuration with output configuration.*

Proof. This property is proved by the folding certificates in Figure S.28. □

S.1.2.2. Zag Rows

The following lemmas show that the molecule folds as expected in the Zag-rows. As Zag-row does not have to deal with carry propagation, its analysis is significantly simpler than the Zig-rows and fewer lemmas are needed to prove the correctness of the folding.

Lemma 8 (Modules A and C-Zag row)**.** *When folded in Zag-row from the output configurations of the preceding brick BT or B2 and under a brick Cxc (resp. Axc), module A (resp. C) folds into the brick Az (resp. Cz) where* $z = x + c \mod 2$ *with output configuration* λ_3 , λ'_3 , λ_4 , λ'_4 *or* λ_5 *which only differ by the positions of their bonds with the environment.*

Proof. This property is proved by the folding certificates in Figures:

- Module A: Figures S.13–S.16.
- Module C: Figures S.41–S.43 for the leftmost column; Figures S.44–S.47 for the inner columns; Figures S.48 and S.49 for the rightmost column. □

Lemma 9 (Modules A and C-Zag row)**.** *When folded in Zag-row from the output configurations of the preceding brick Ax (resp. Cx) and under the bricks BzAyc (resp. DzCyc in an inner column or DT in the rightmost column), module B (resp. D) folds into the brick B2 (resp. D2) with output configuration* λ'_2 *if* $y + c \leqslant 1$ *and output configurations* $\mu \cup \mu'$ *if* $y + c = 2$.

Proof. This property is proved by the folding certificates in Figures:

- Module B: Figures S.29–S.31 for $x = 0$ and $y + c \leqslant 1$; Figures S.33–S.35 for $x = 1$ and $y + c \leqslant 1$; Figures S.32 and S.36 for $y + c = 2$.
- Module D: Figures S.58–S.60 for $x = 0$ and $y + c \leqslant 1$; Figures S.62–S.64 for $x = 1$ and $y + c \leqslant 1$; Figures S.61 and S.65 for $y + c = 2$; Figures S.66 and S.67 for the rightmost column. □

Lemma 10 (Modules A and C-inner column-Zag row-no carry propagation)**.** *When folded in an inner column of a Zig-row from the output configurations of the preceding brick Dc (resp. Bc) and under a brick Cx (resp. Ax) s.t.* $x + c \leqslant 1$ *(i.e., no carry propagation), module A (resp. C) folds into the brick* $A(x + c)$ *(resp.* $C(x + c)$*) with output configurations* β.

Proof. This property is proved by the folding certificates in Figures:

- Module A: Figures S.3 and S.5–S.7.
- Module C: Figures S.37–S.39. □

All the folding tree certificates are given in the next supplementary section.

S.2. Folding Certificates

The following sections contain the certificates for the folding of each of modules in all possible environments in a human-readable and -checkable form (up to zooming in the PDF file for some of them). When reading these certificates, the top circles represent the input nascent configurations inherited from the end of folding of the previous module (there might be several of them). Then, the number of new bonds of each of the local configurations is written in the top left corner of that configuration. The path in bold shows the configurations with the maximum number of bonds, that is to say the only ones that are allowed to continue to grow by the inertial dynamics. To improve readability, we group in the same ball all paths that share a common prefix up to their last bond; the free end of each path in the group is drawn in random color; the size of the group is given in the lower right corner for cross-checking. The last level of the folding tree represents the output nascent configurations, that is the only configurations allowed to start the folding of the next module.

S.2.1. Folding Certificate for the Output Nascent Configuration of the Seed

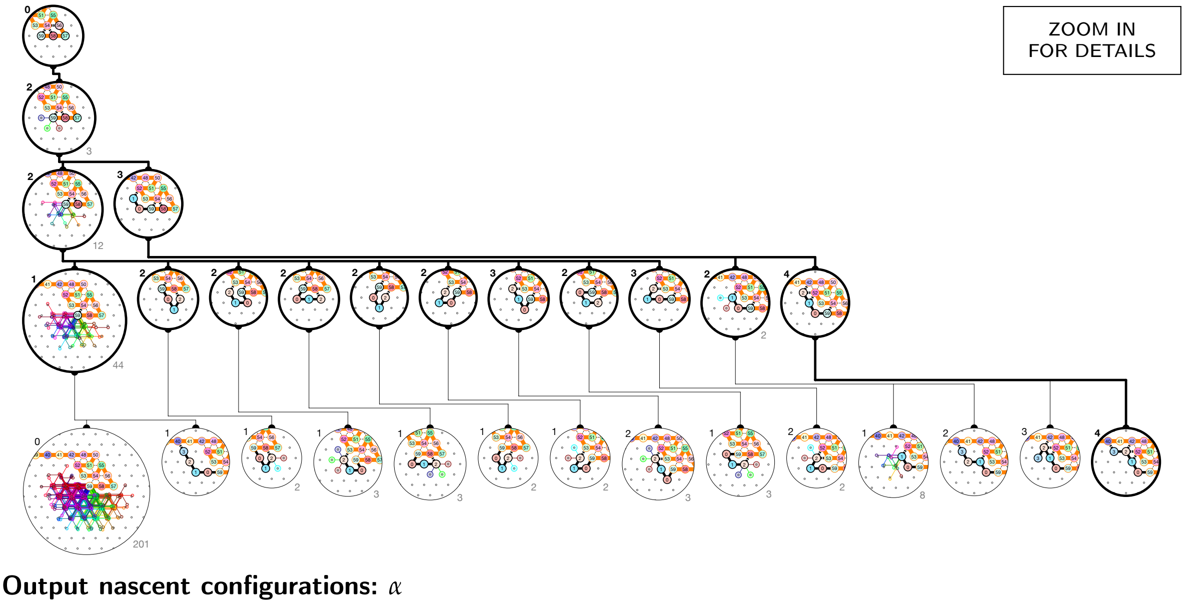

Figure S.2. The output nascent configurations of the seed brick–Region #0 in the 5-bits counter. The first level consists of the seed configuration σ. The next three levels consist of all the possible extensions of σ by $i = 1..3$ beads (recall $3 = \delta - 1$). The last level is the first of the inertial dynamics: it outputs only one nascent configuration, α , with maximum number of bonds.

S.2.2. Folding Certificates for Module A: First Half-Adder

Input nascent configurations: α

ZOOM IN
FOR DETAILS

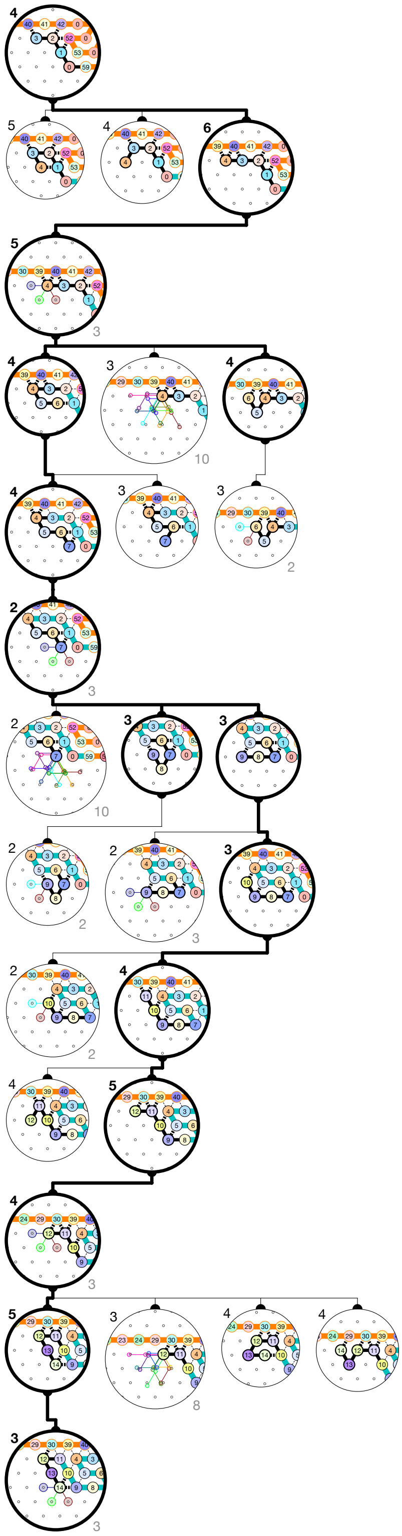

Output nascent configurations: β

Figure S.3. Folding of Brick A01 in 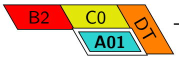

–First occurrence in 5-bits counter: Region #1.

Input nascent configurations: α

ZOOM IN
FOR DETAILS

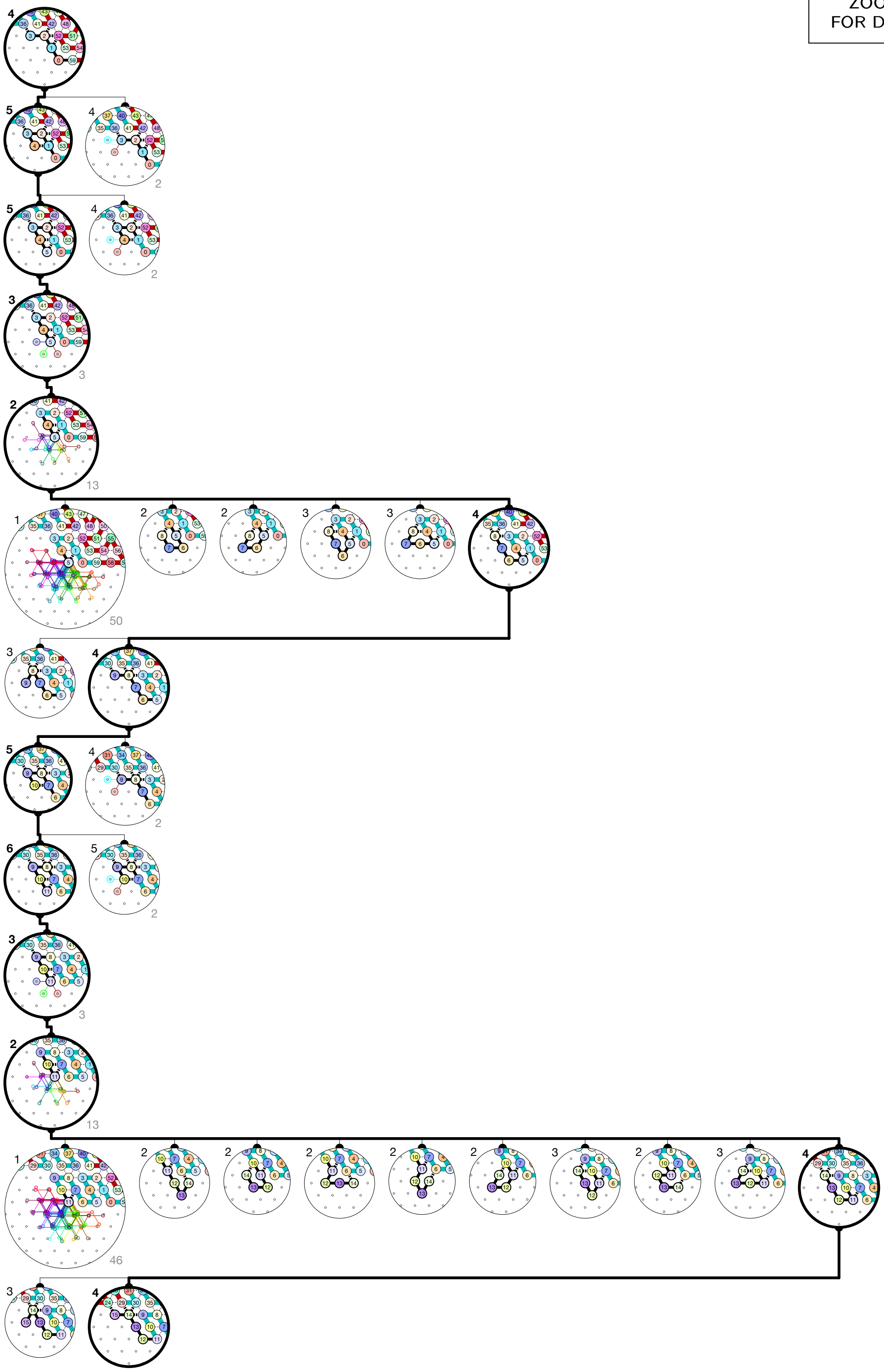

Output nascent configurations: α'

Figure S.4. Folding of Brick A11 in 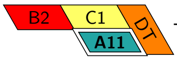

–First occurrence in 5-bits counter: Region #21.

Input nascent configurations: $\theta \cup \theta'$

ZOOM IN
FOR DETAILS

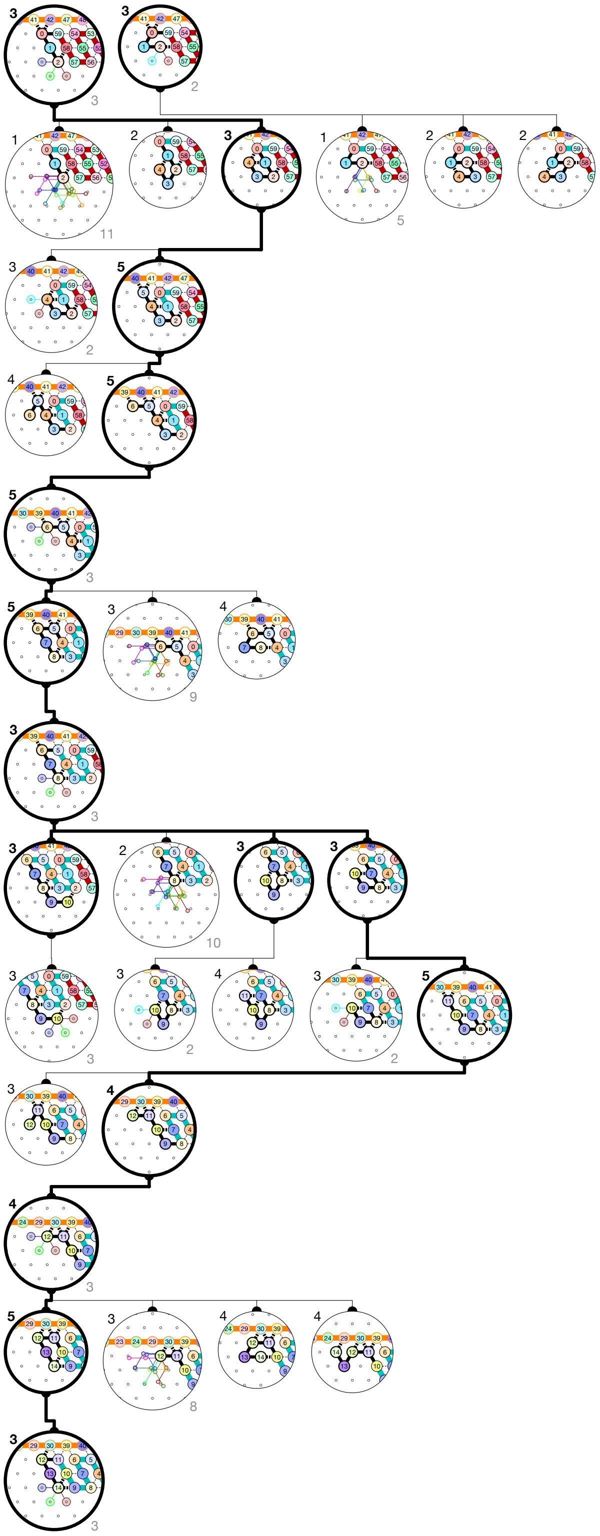

Output nascent configurations: β

Figure S.5. Folding of Brick A00 in 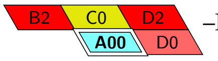

–First occurrence in 5-bits counter: Region #5.

Input nascent configurations: α''

ZOOM IN
FOR DETAILS

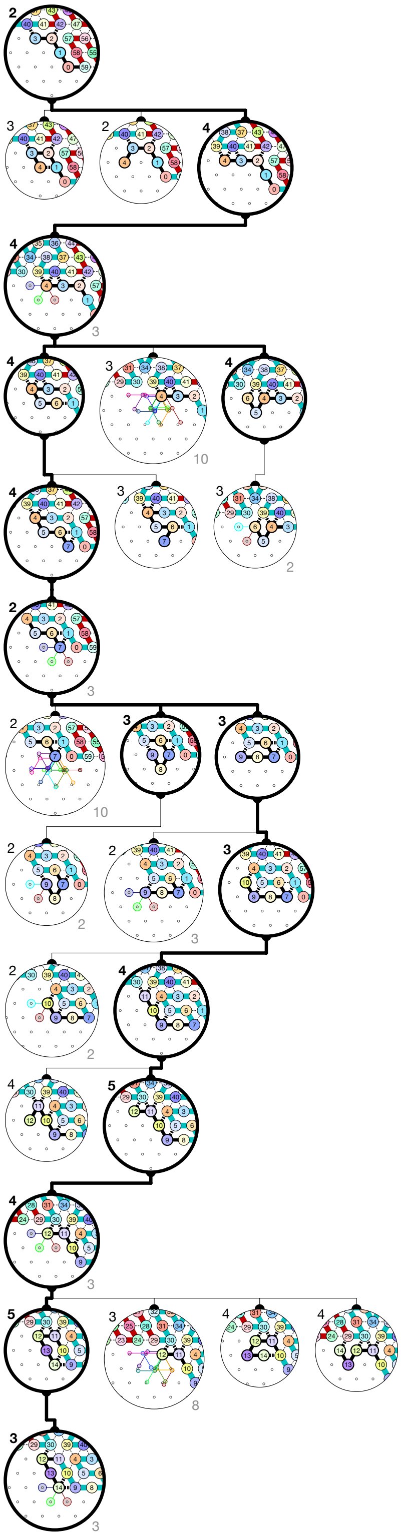

Output nascent configurations: β

Figure S.6. Folding of Brick A01 in

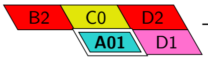

–First occurrence in 5-bits counter: Region #65.

Input nascent configurations: γ'

ZOOM IN
FOR DETAILS

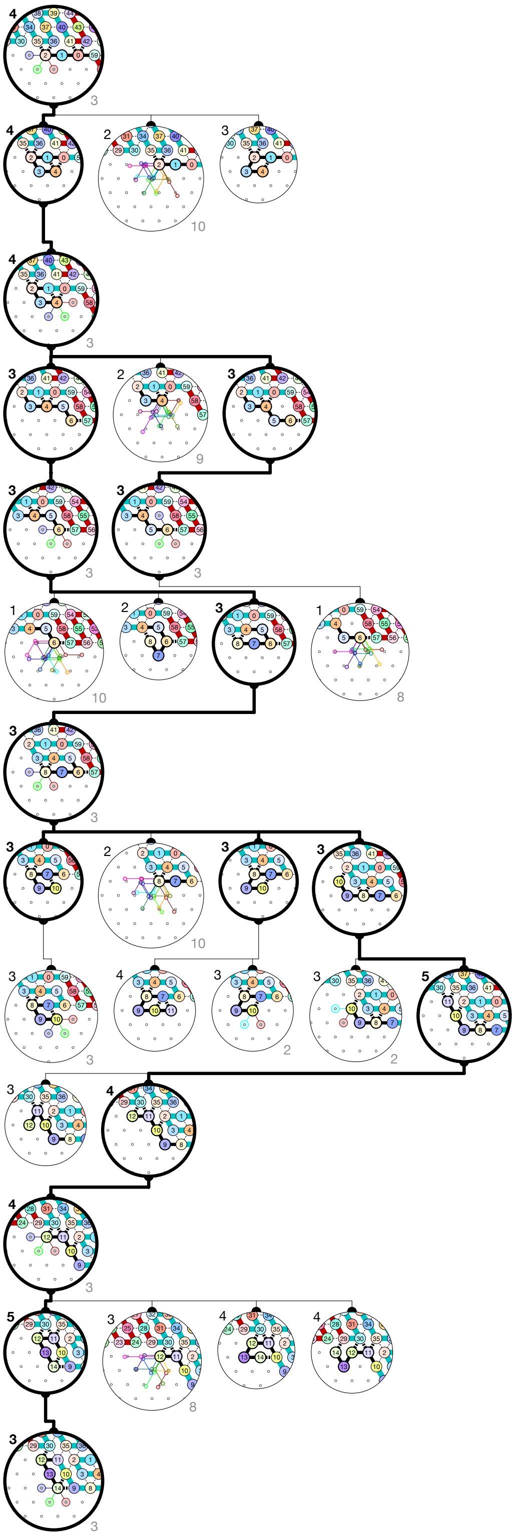

Output nascent configurations: β

Figure S.7. Folding of Brick A10 in

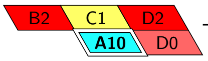

–First occurrence in 5-bits counter: Region #85.

Input nascent configurations: α''

ZOOM IN
FOR DETAILS

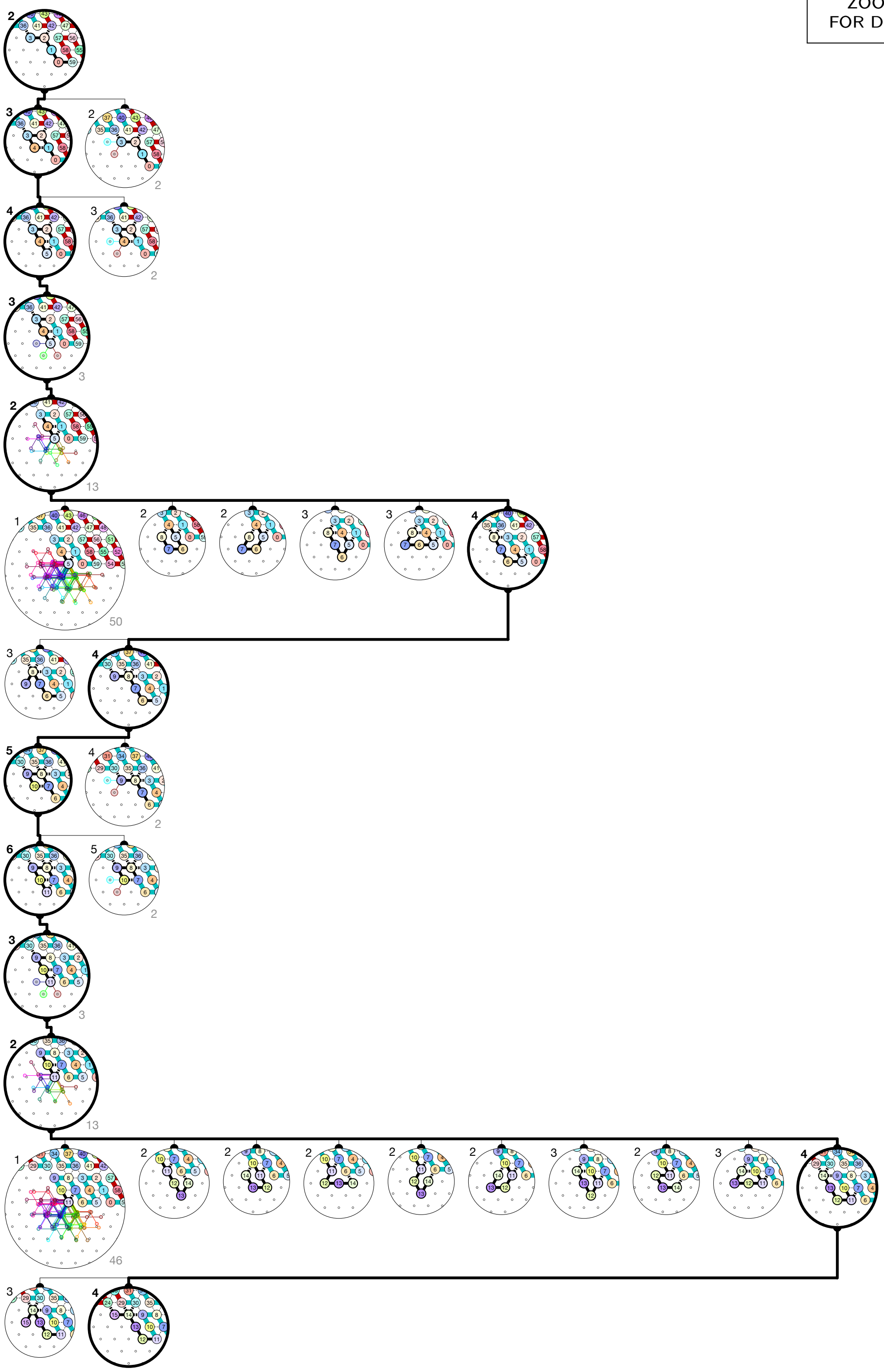

Output nascent configurations: α'

Figure S.8. Folding of Brick A11 in B2 C1 D2 A11 D1 –First occurrence in 5-bits counter: Region #145.

Input nascent configurations: $\theta \cup \theta'$

ZOOM IN
FOR DETAILS

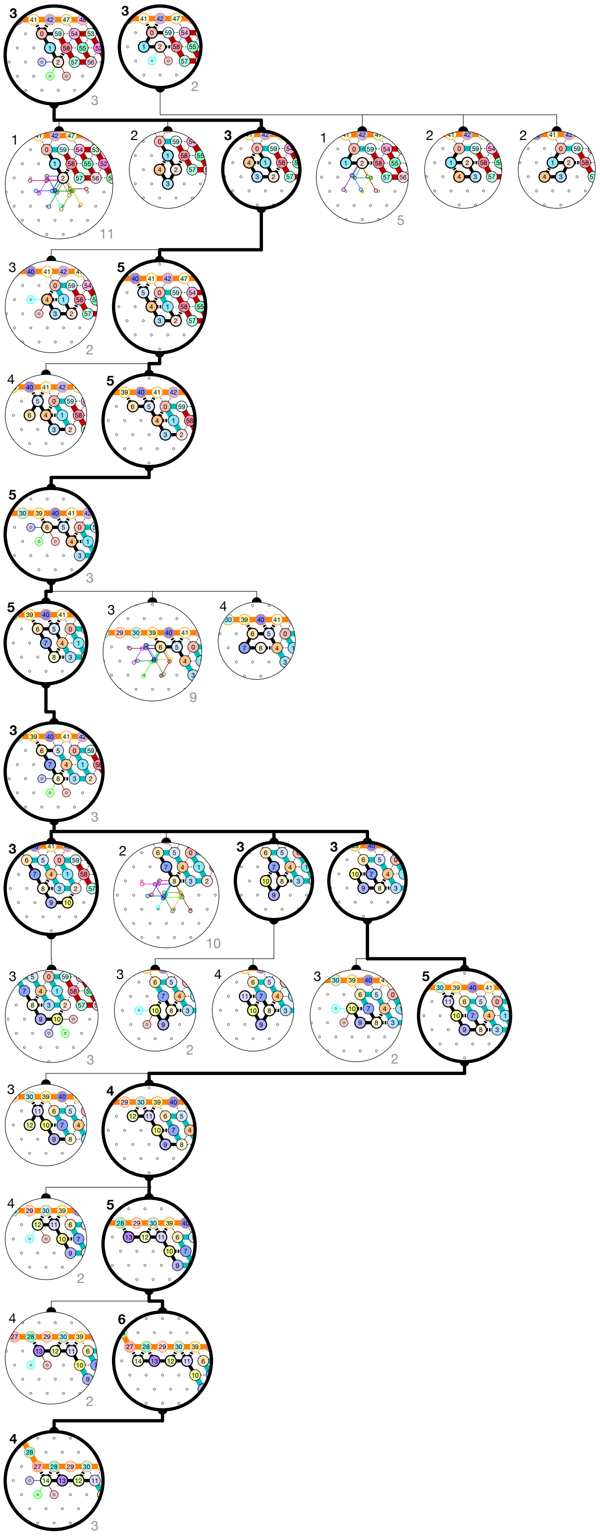

Output nascent configurations: γ

Figure S.9. Folding of Brick A00 in 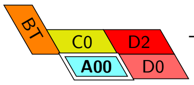 –First occurrence in 5-bits counter: Region #9.

Input nascent configurations: α''

ZOOM IN
FOR DETAILS

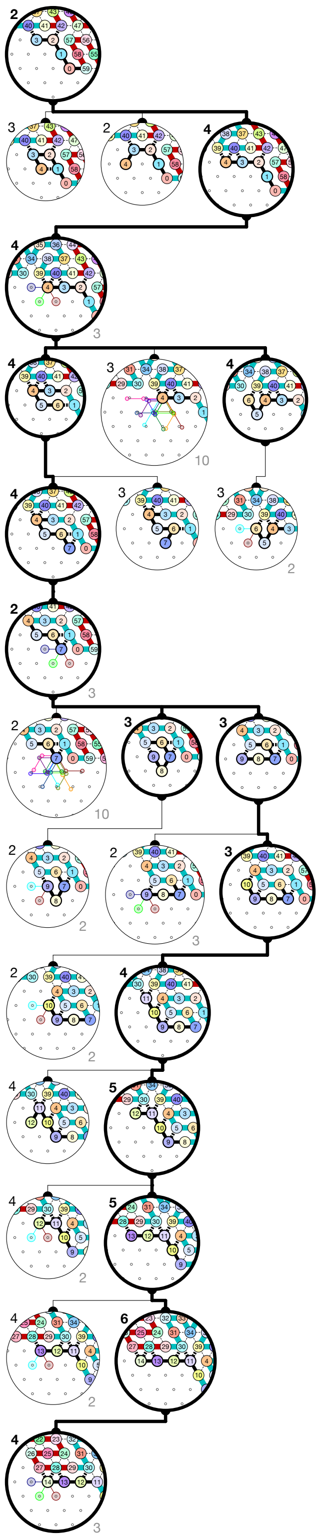

Output nascent configurations: γ

Figure S.10. Folding of Brick A01 in 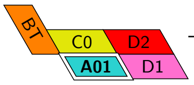

–First occurrence in 5-bits counter: Region #309.

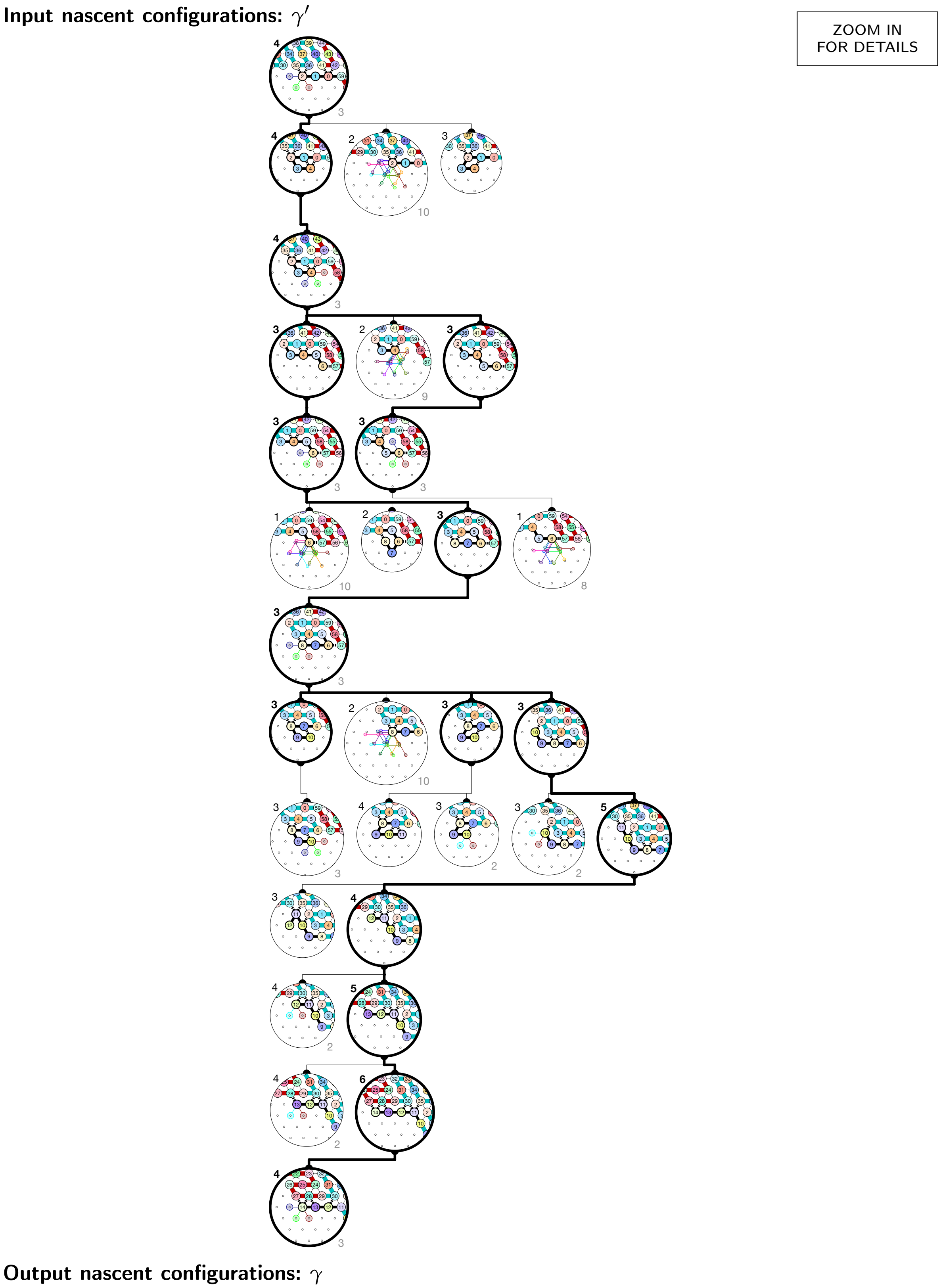

Figure S.11. Folding of Brick A10 in

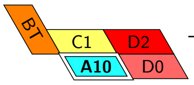

–First occurrence in 5-bits counter: Region #329.

Input nascent configurations: α''

ZOOM IN
FOR DETAILS

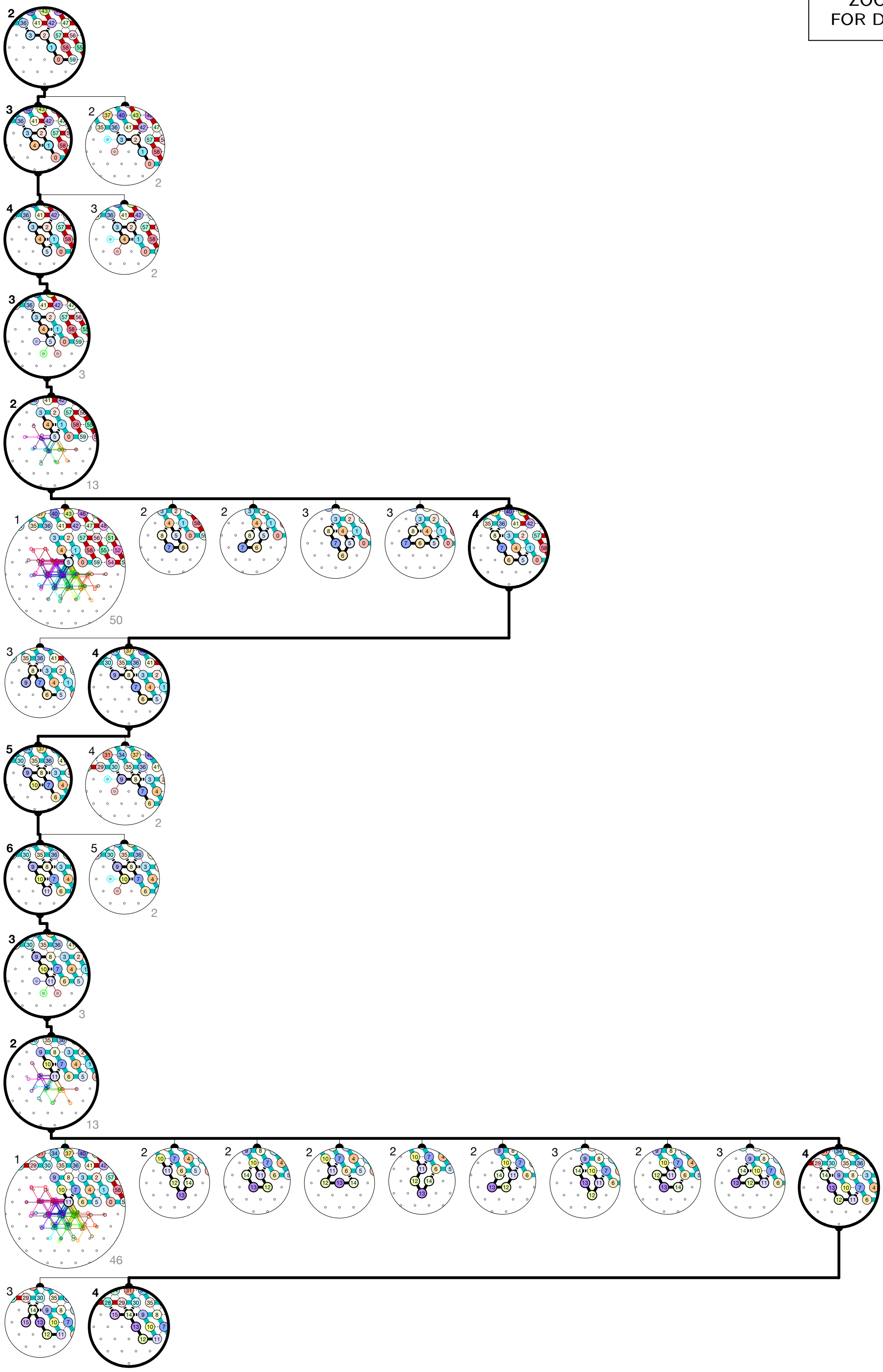

Output nascent configurations: α'

Figure S.12. Folding of Brick A11 in 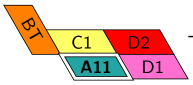

–First occurrence in 5-bits counter: Region #629.

Input nascent configurations: λ_2'

ZOOM IN
FOR DETAILS

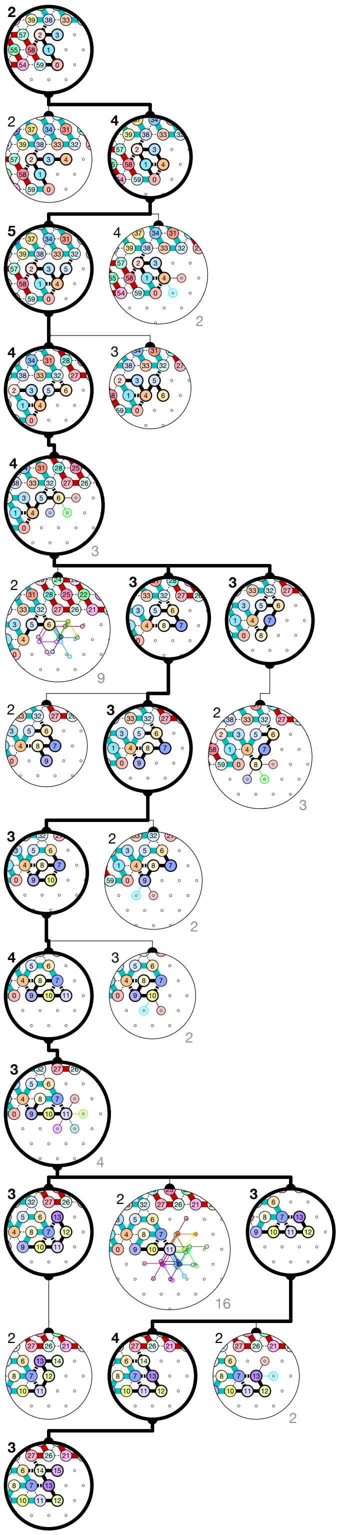

Output nascent configurations: λ_3

Figure S.13. Folding of Brick A0 in [C00 B0 D2 A0] –First occurrence in 5-bits counter: Region #13.

Input nascent configurations: λ_2'

ZOOM IN
FOR DETAILS

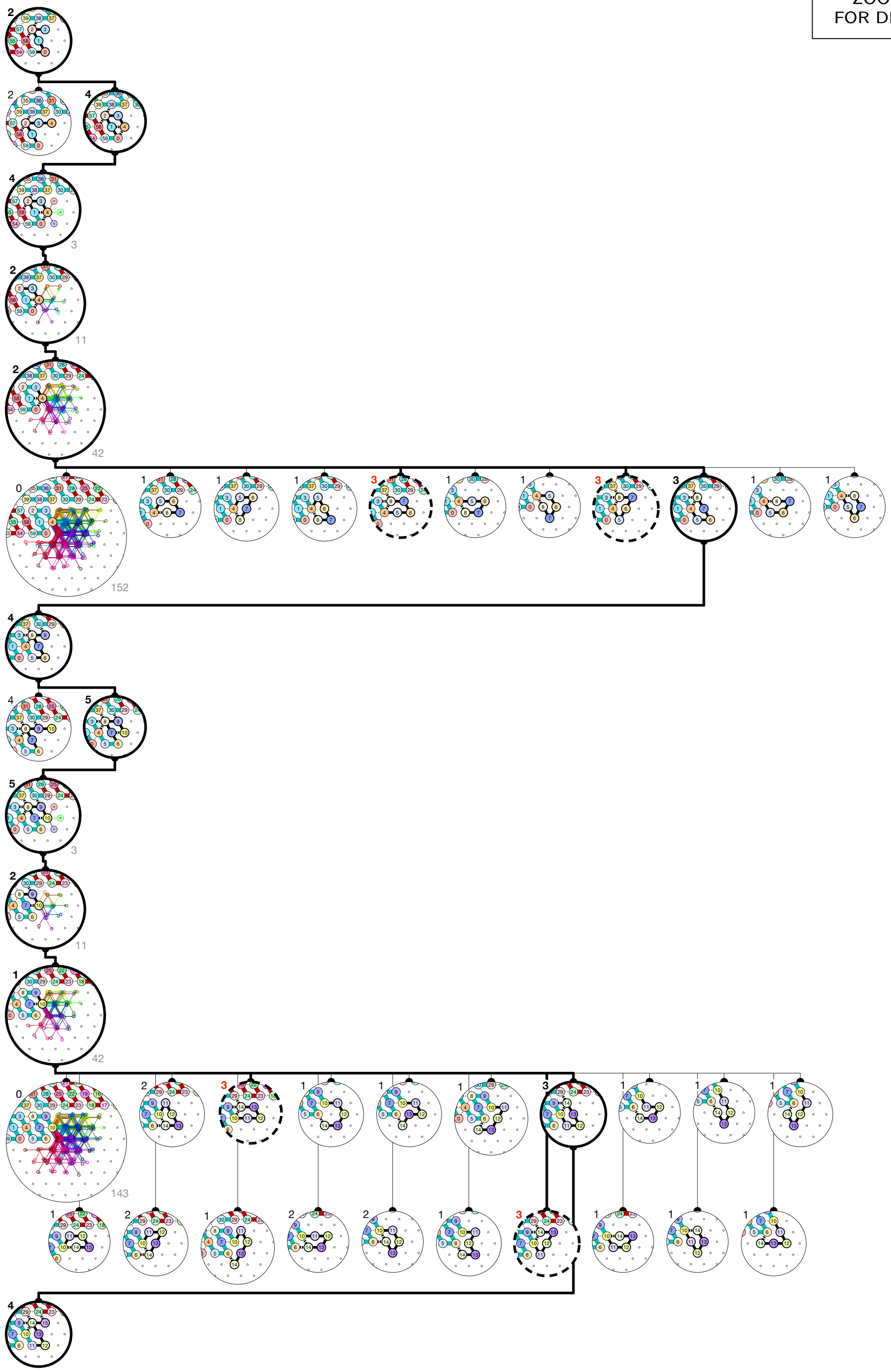

Output nascent configurations: λ_4

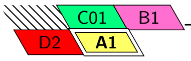

Figure S.14. Folding of Brick A1 in C01 B1 D2 A1 –First occurrence in 5-bits counter: Region #37.

ZOOM IN
FOR DETAILS

Input nascent configurations: λ'_2

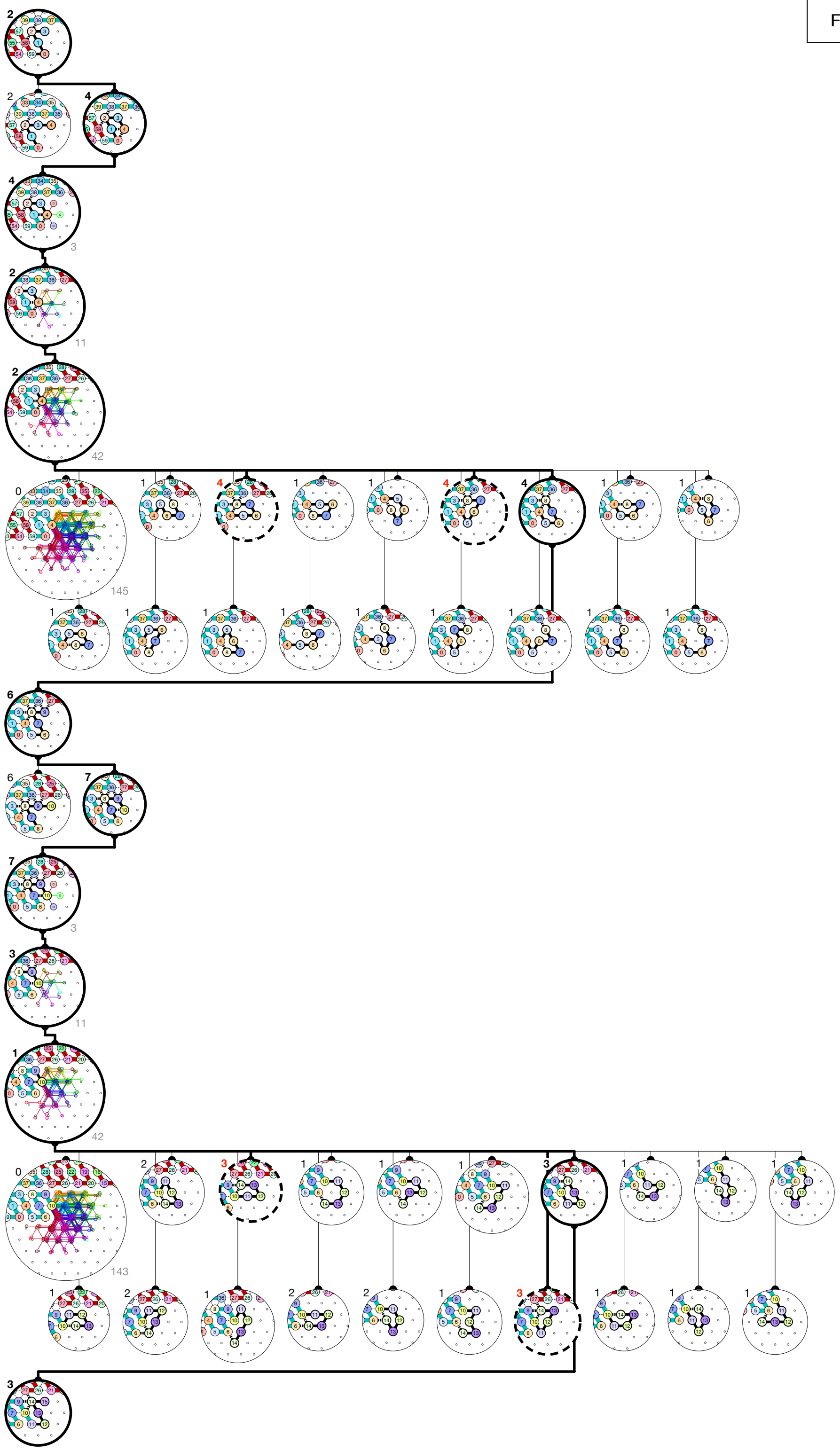

Output nascent configurations: λ'_3

Figure S.15. Folding of Brick A1 in C10 B0 D2 A1 –First occurrence in 5-bits counter: Region #57.

Input nascent configurations: $\mu \cup \mu'$

ZOOM IN
FOR DETAILS

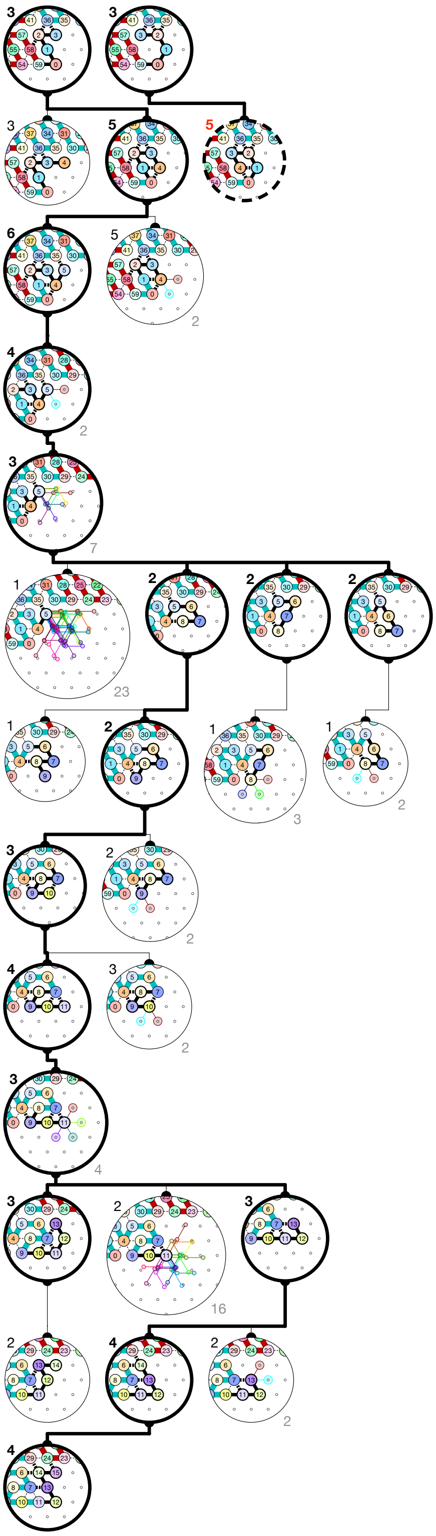

Output nascent configurations: λ'_4

Figure S.16. Folding of Brick A0 in 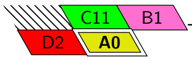

–First occurrence in 5-bits counter: Region #77.

S.2.3. Folding certificates for Module B: Left Turn

Input nascent configurations: β

ZOOM IN
FOR DETAILS

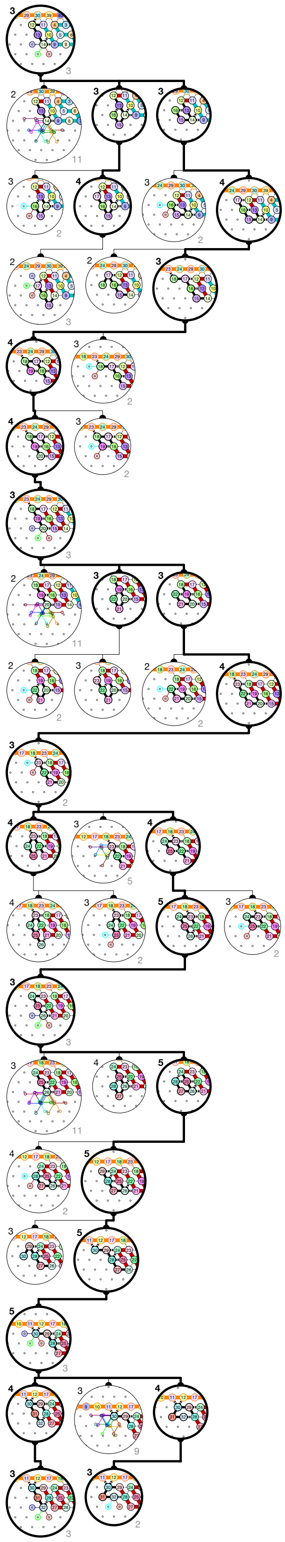

Output nascent configurations: $\theta \cup \theta'$

Figure S.17. Folding of Brick B0 in 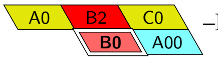

–First occurrence in 5-bits counter: Region #2.

Input nascent configurations: β

ZOOM IN
FOR DETAILS

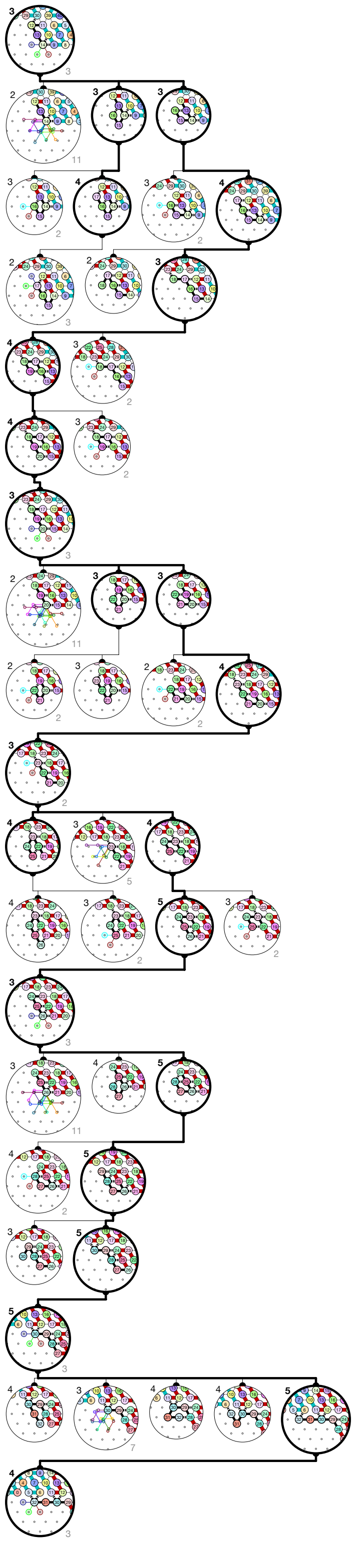

Output nascent configurations: γ'

Figure S.18. Folding of Brick B0 in A1 B2 C0 B0 A00 –First occurrence in 5-bits counter: Region #166.

Input nascent configurations: β

ZOOM IN
FOR DETAILS

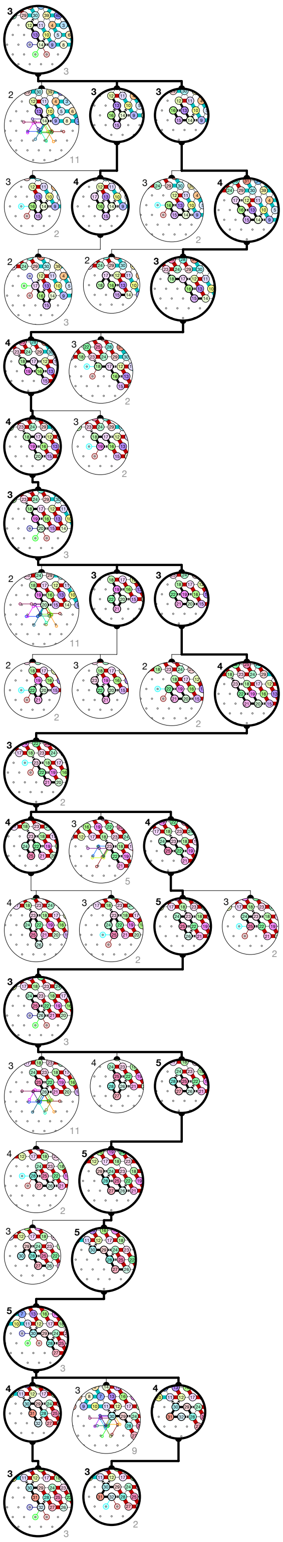

Output nascent configurations: $\theta \cup \theta'$

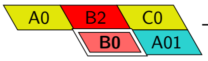

Figure S.19. Folding of Brick B0 in A0 B2 C0 B0 A01 –First occurrence in 5-bits counter: Region #66.

Input nascent configurations: β

ZOOM IN
FOR DETAILS

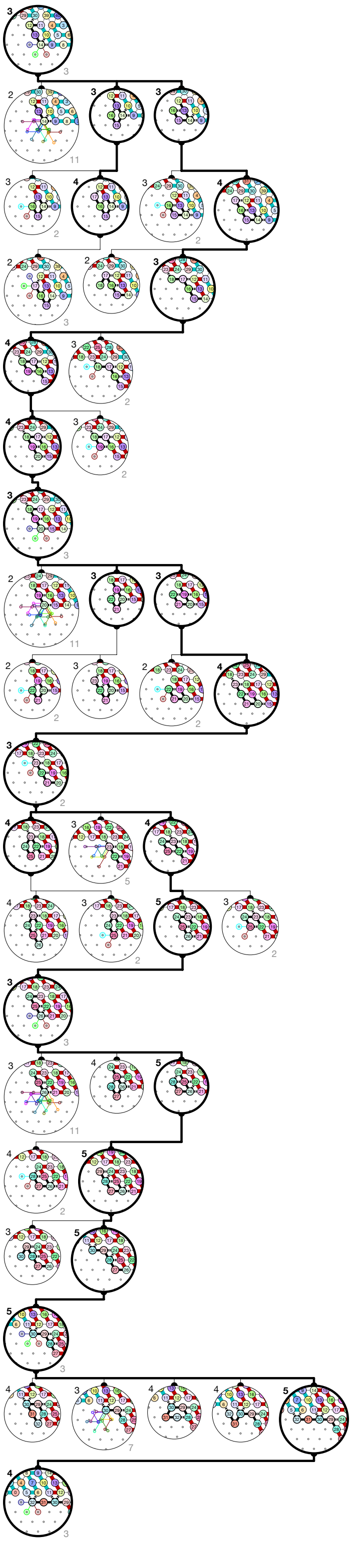

Output nascent configurations: γ'

Figure S.20. Folding of Brick B0 in A1 B2 C0 B0 A01 –First occurrence in 5-bits counter: Region #42.

Input nascent configurations: β

ZOOM IN
FOR DETAILS

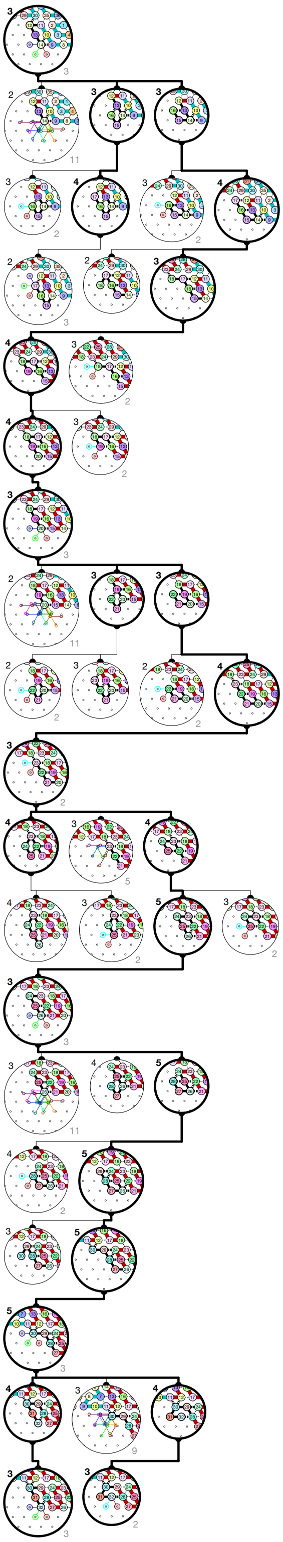

Output nascent configurations: $\theta \cup \theta'$

Figure S.21. Folding of Brick B0 in 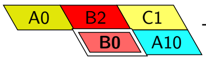

–First occurrence in 5-bits counter: Region #86.

Input nascent configurations: β

ZOOM IN
FOR DETAILS

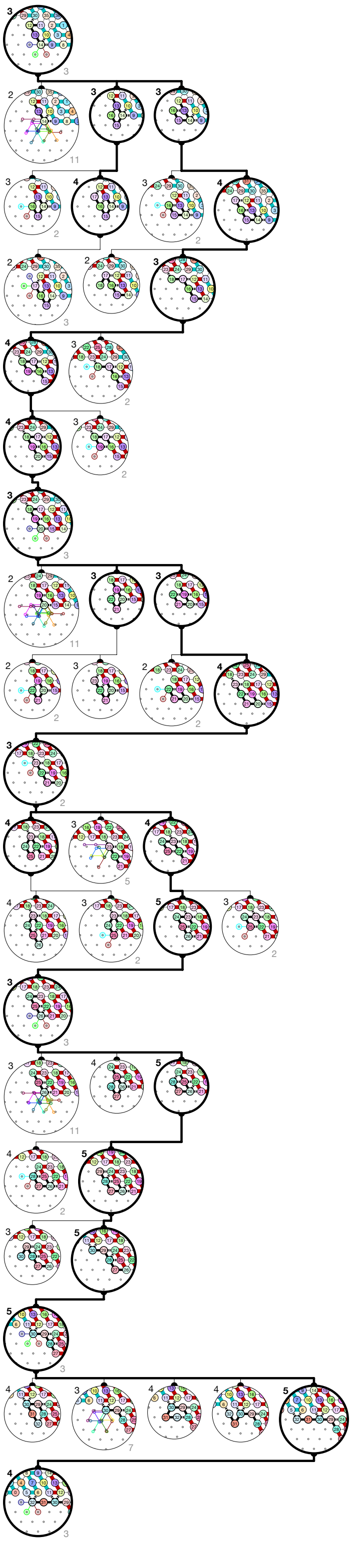

Output nascent configurations: γ'

Figure S.22. Folding of Brick B0 in A1 B2 C1 B0 A10 –First occurrence in 5-bits counter: Region #246.

Input nascent configurations: α'

ZOOM IN
FOR DETAILS

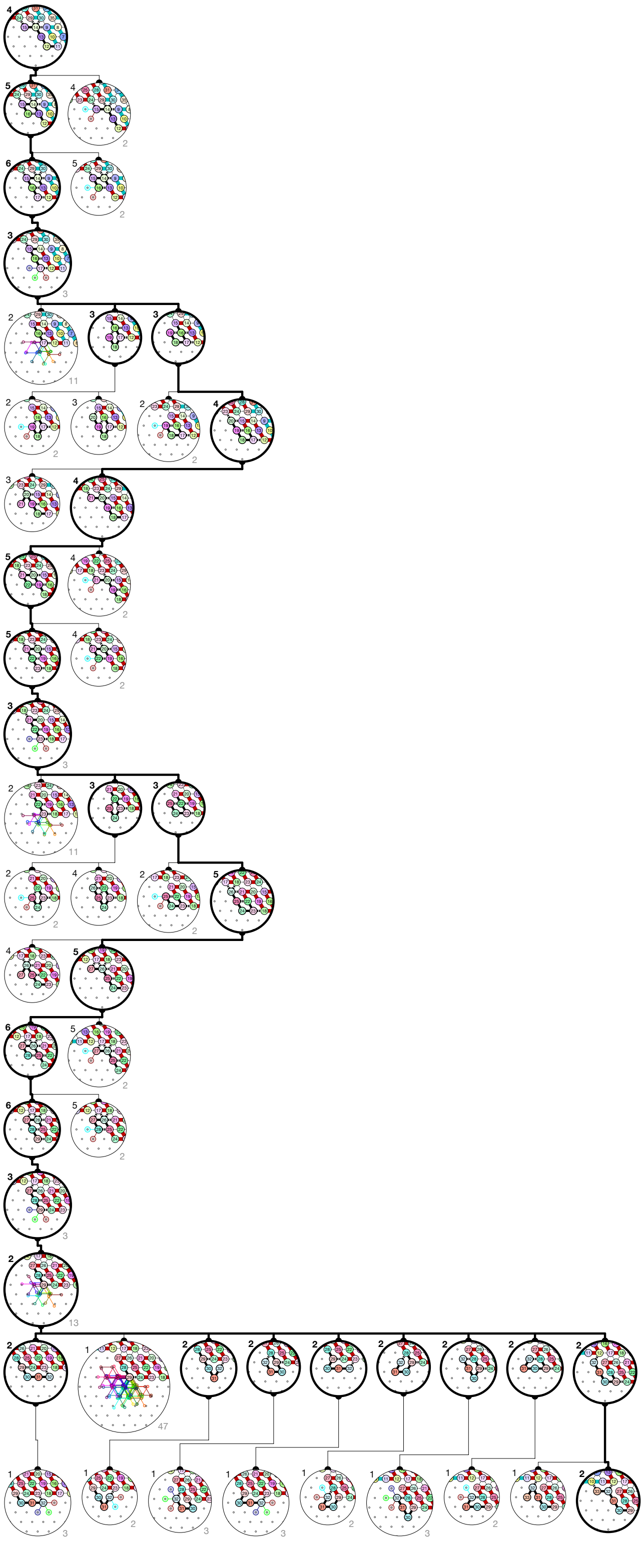

Output nascent configurations: α''

Figure S.23. Folding of Brick B1 in 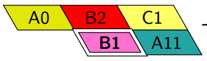 –First occurrence in 5-bits counter: Region #22.

Input nascent configurations: α'

ZOOM IN
FOR DETAILS

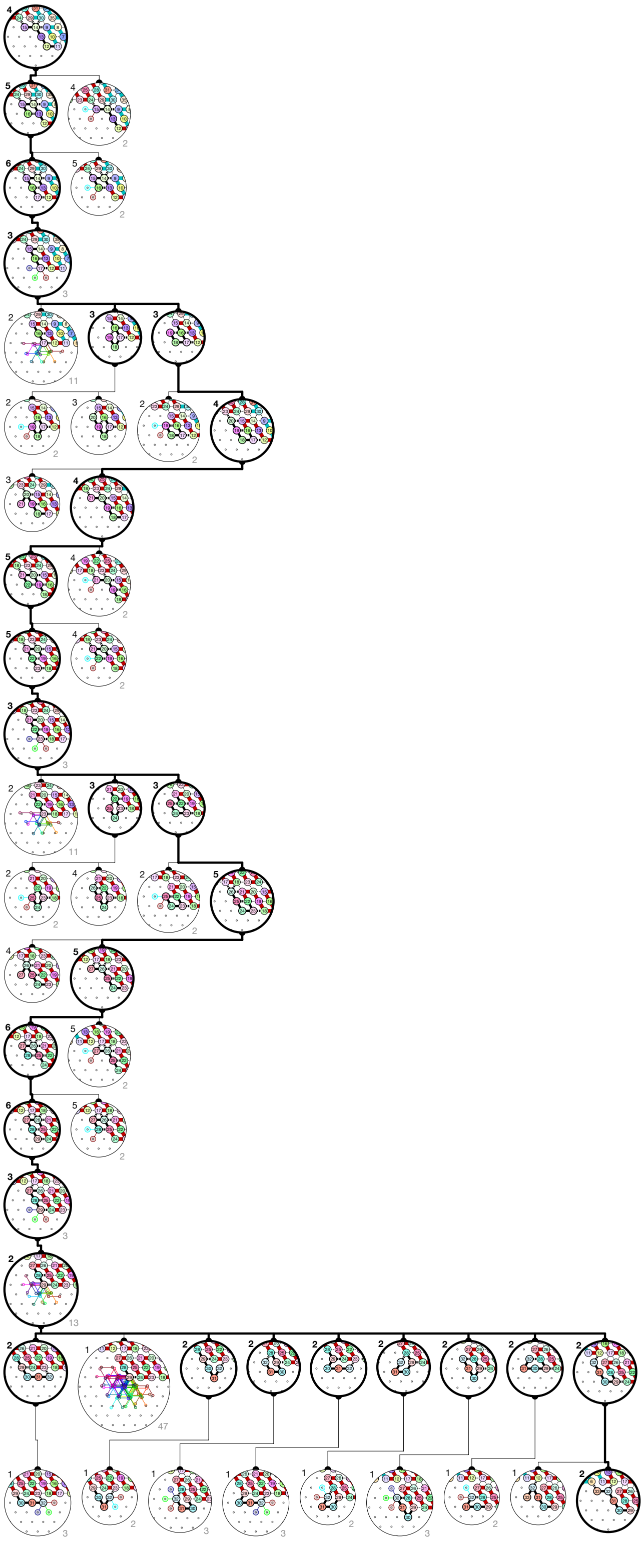

Output nascent configurations: α''

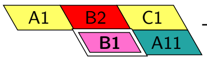

Figure S.24. Folding of Brick B1 in –First occurrence in 5-bits counter: Region #62.

Input nascent configurations: γ

ZOOM IN
FOR DETAILS

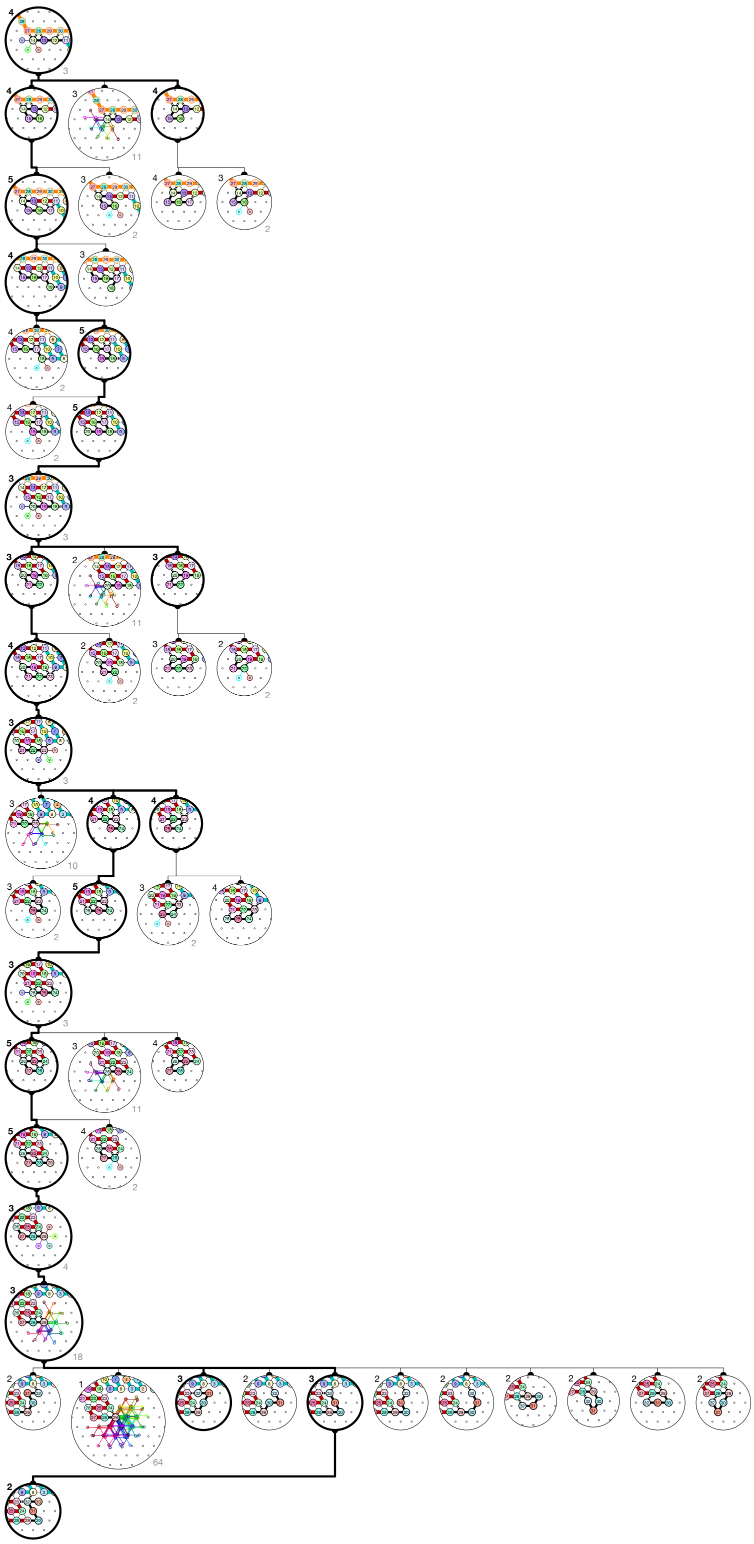

Output nascent configurations: λ_2

Figure S.25. Folding of Brick BT in 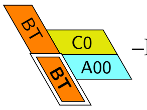

–First occurrence in 5-bits counter: Region #10.

Input nascent configurations: γ

ZOOM IN
FOR DETAILS

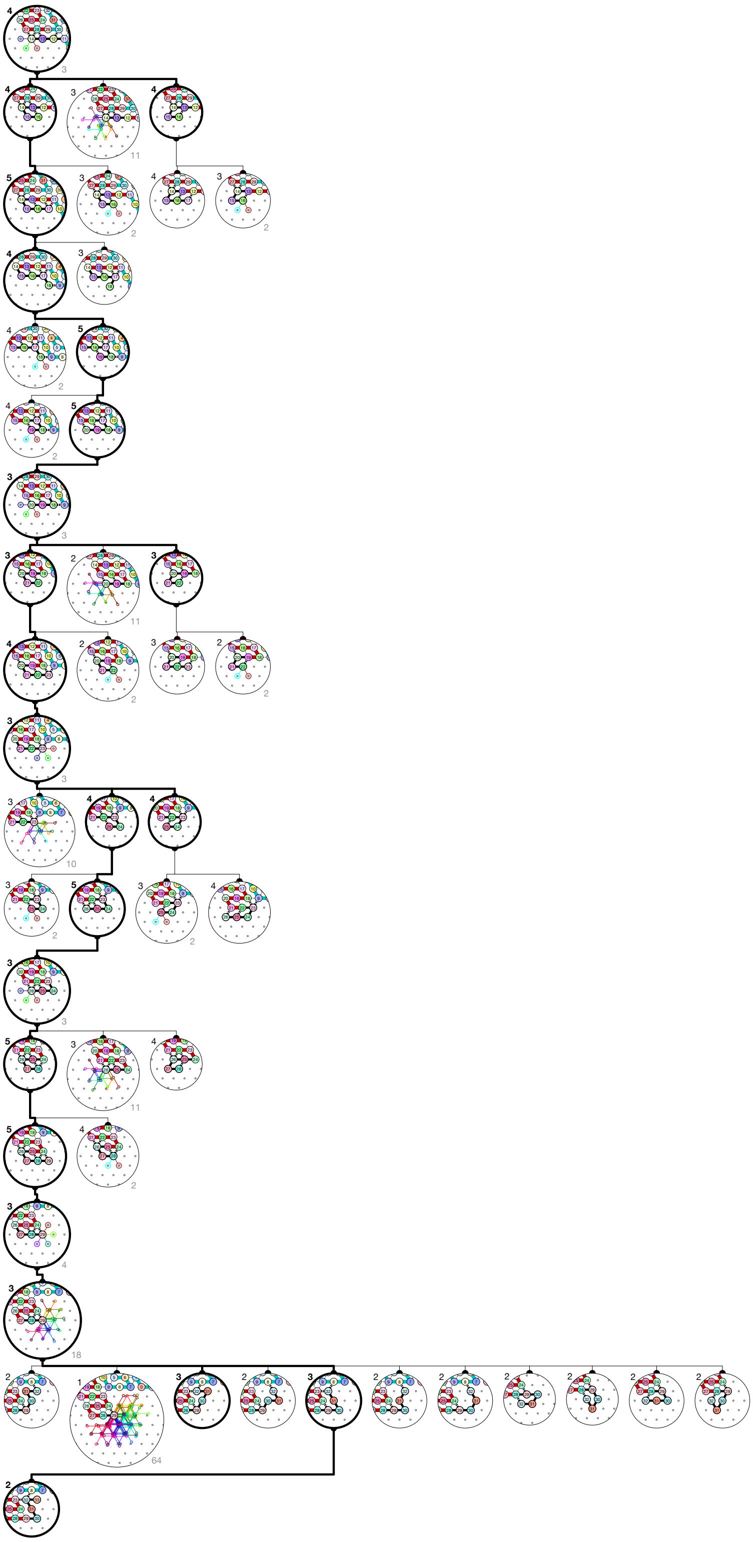

Output nascent configurations: λ_2

Figure S.26. Folding of Brick BT in 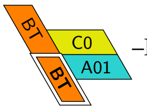

–First occurrence in 5-bits counter: Region #310.

Input nascent configurations: γ

ZOOM IN
FOR DETAILS

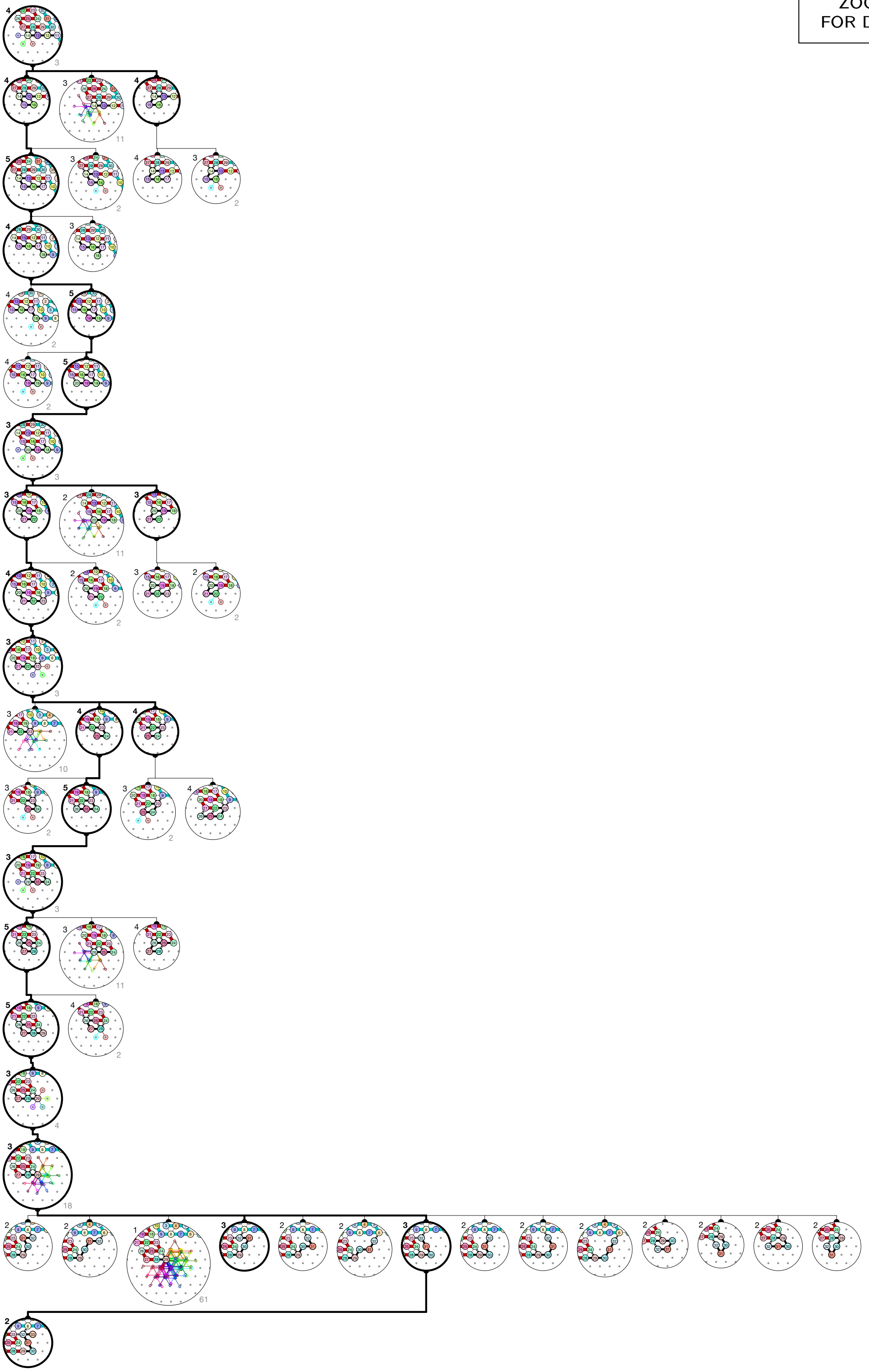

Output nascent configurations: λ_2

Figure S.27. Folding of Brick BT in 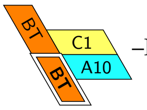

–First occurrence in 5-bits counter: Region #330.

Input nascent configurations: α'

ZOOM IN
FOR DETAILS

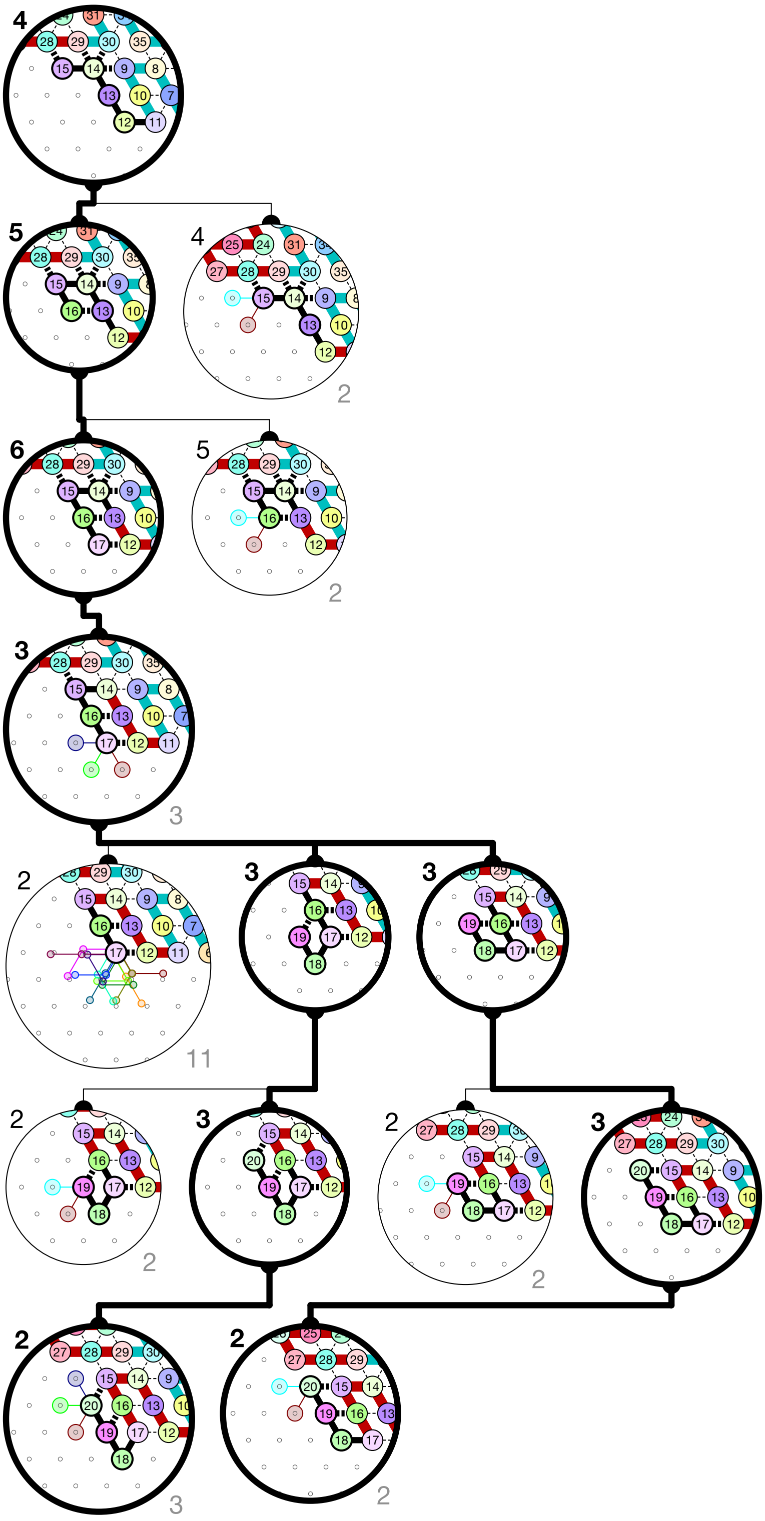

Output nascent configurations: Halt

Figure S.28. Folding of Brick Halt in 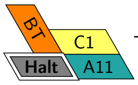

–First occurrence in 5-bits counter: Region #630.

ZOOM IN
FOR DETAILS

Input nascent configurations: λ_3

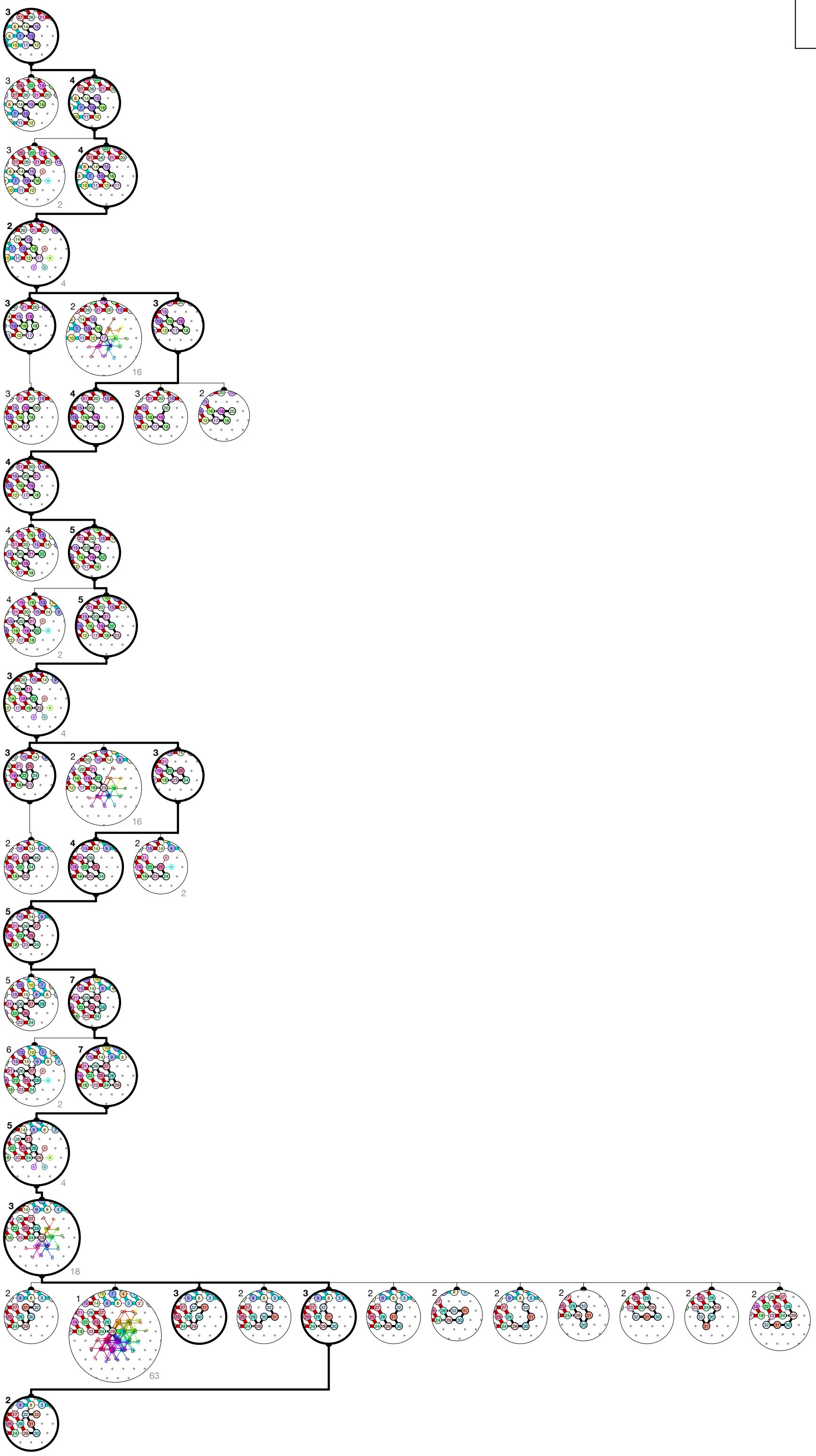

Output nascent configurations: λ'_2

Figure S.29. Folding of Brick B2 in 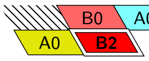–First occurrence in 5-bits counter: Region #14.

Input nascent configurations: λ_3

ZOOM IN
FOR DETAILS

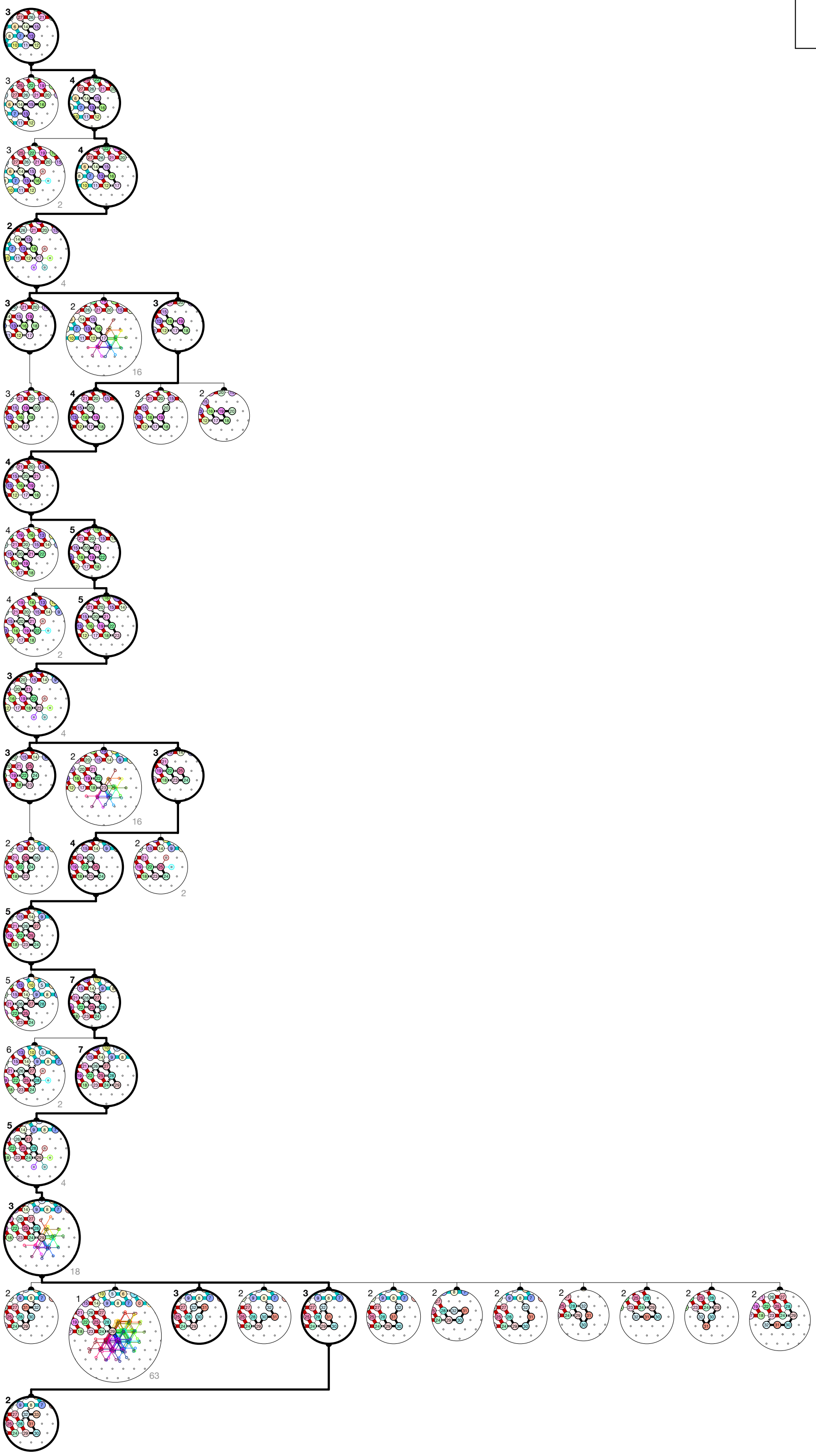

Output nascent configurations: λ_2'

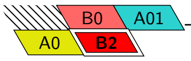

Figure S.30. Folding of Brick B2 in –First occurrence in 5-bits counter: Region #18.

Input nascent configurations: λ_3

ZOOM IN
FOR DETAILS

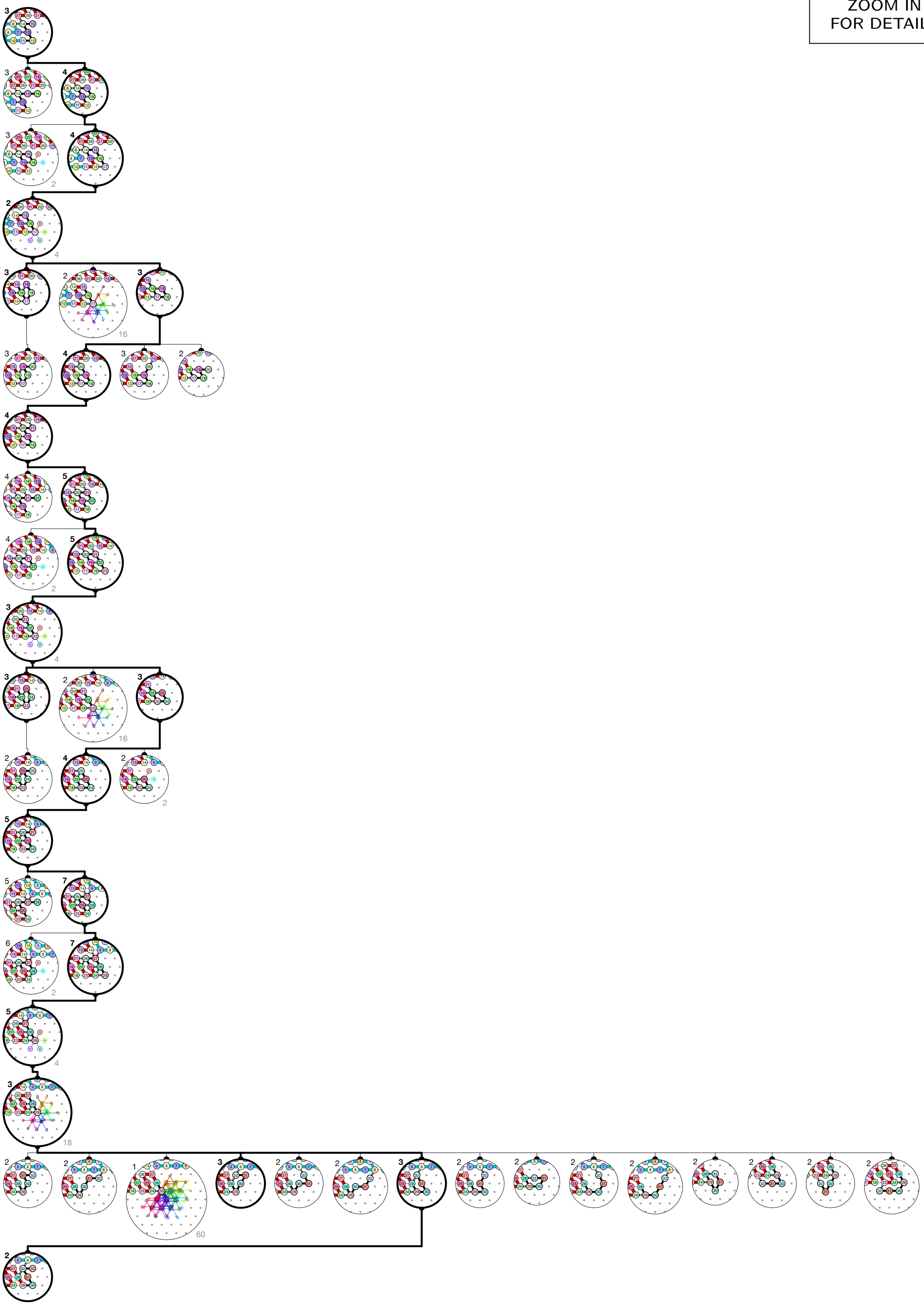

Output nascent configurations: λ'_2

Figure S.31. Folding of Brick B2 in

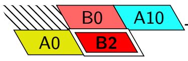

–First occurrence in 5-bits counter: Region #94.

Input nascent configurations: λ'_4

ZOOM IN
FOR DETAILS

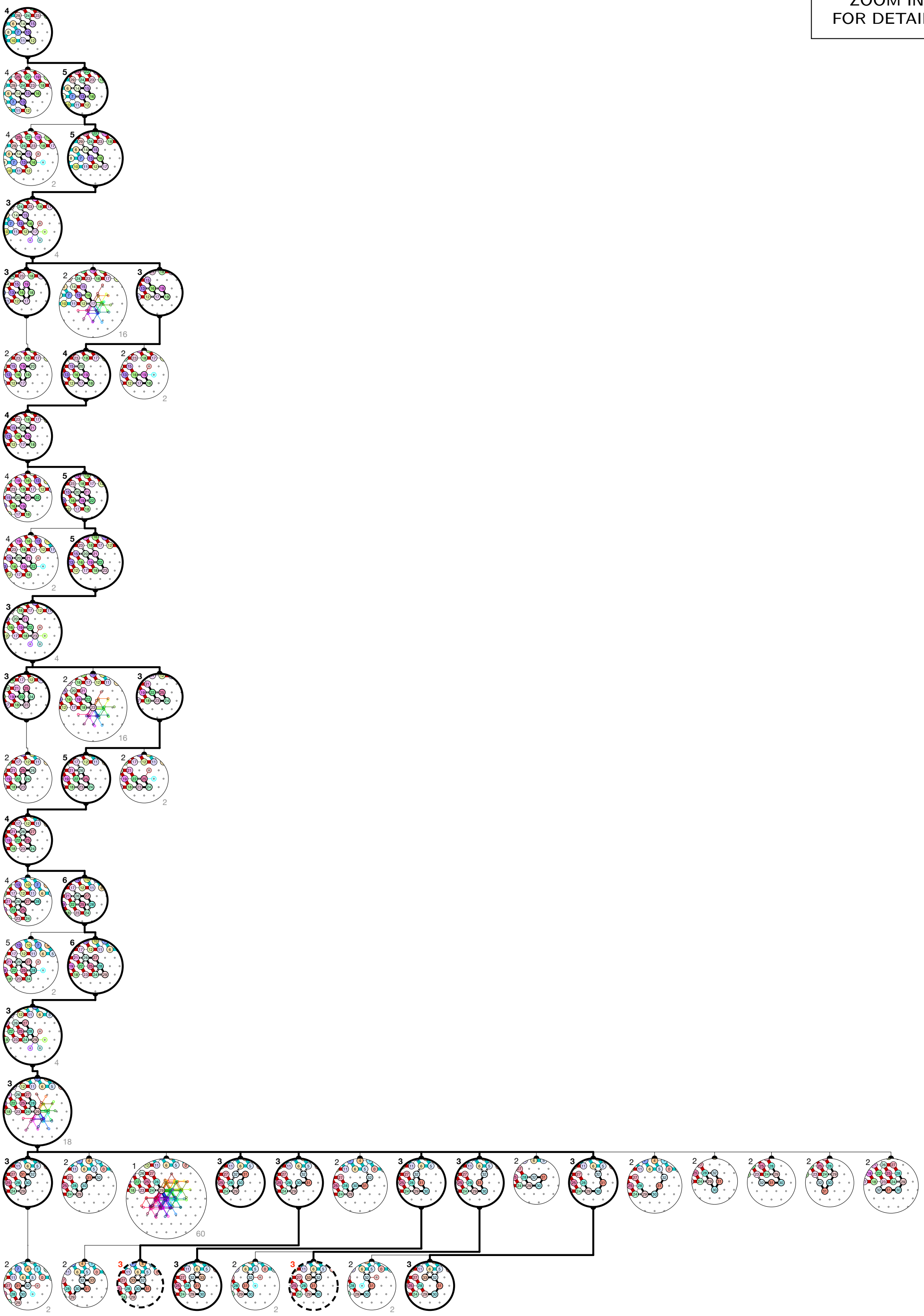

Output nascent configurations: $\mu \cup \mu'$

Figure S.32. Folding of Brick B2 in B1 A11 A0 B2 –First occurrence in 5-bits counter: Region #78.

Input nascent configurations: λ_3'

ZOOM IN
FOR DETAILS

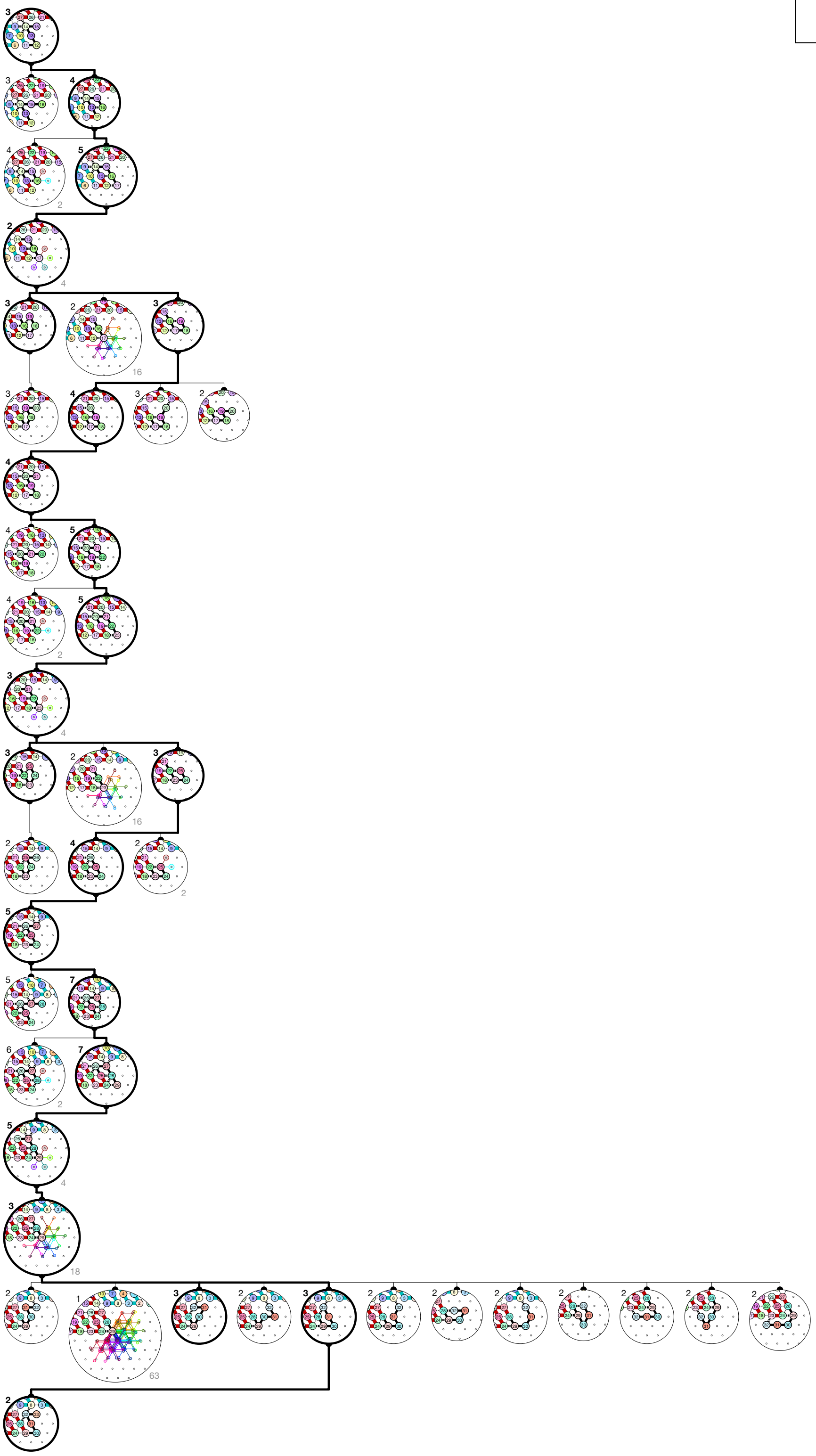

Output nascent configurations: λ_2'

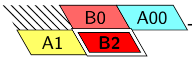

Figure S.33. Folding of Brick B2 in [brick diagram: B0, A00, A1, B2] –First occurrence in 5-bits counter: Region #174.

Input nascent configurations: λ_3'

ZOOM IN
FOR DETAILS

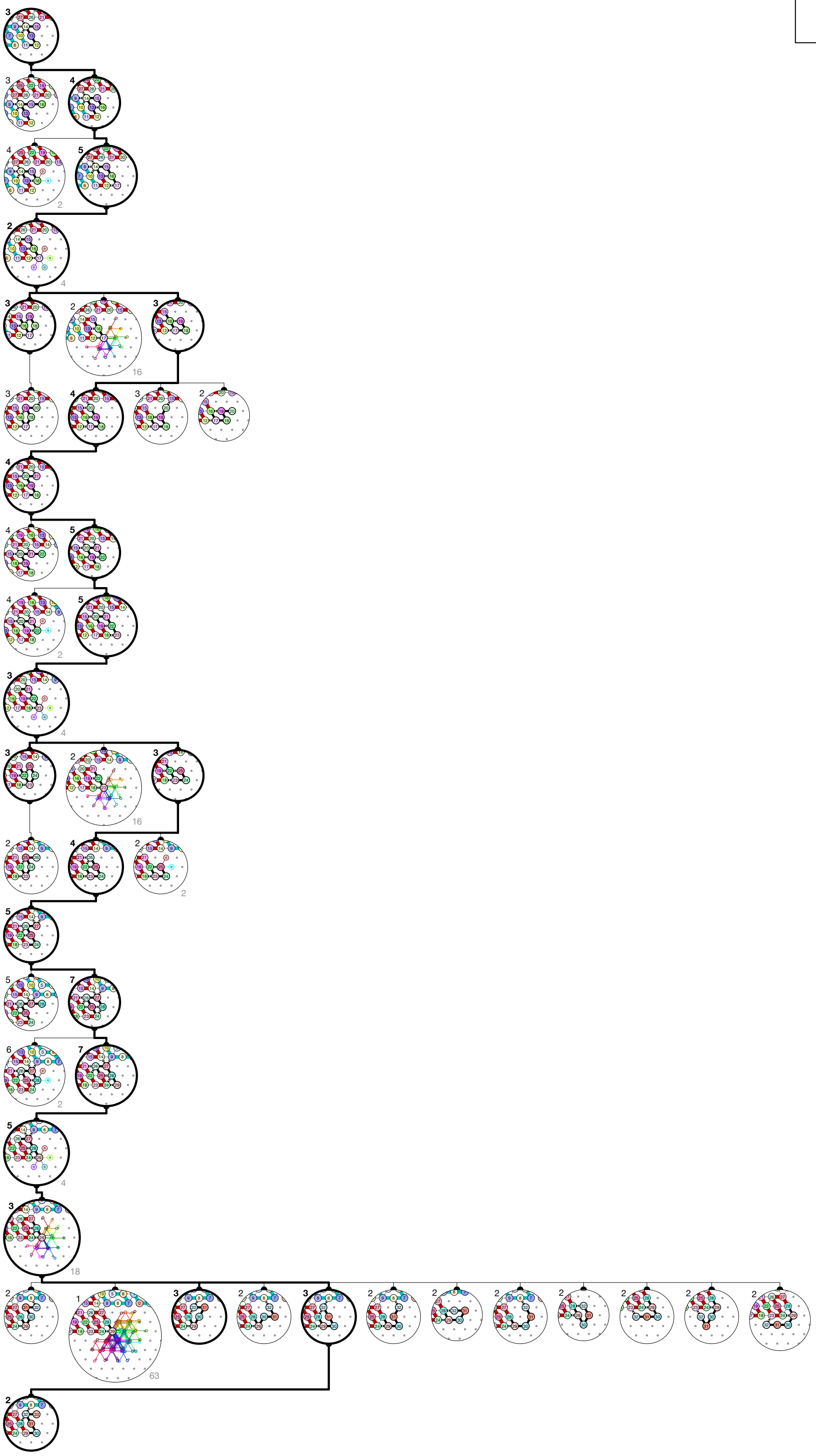

Output nascent configurations: λ_2'

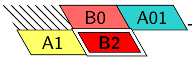

Figure S.34. Folding of Brick B2 in [B0 A01 A1 B2]–First occurrence in 5-bits counter: Region #138.

Input nascent configurations: λ'_3

ZOOM IN
FOR DETAILS

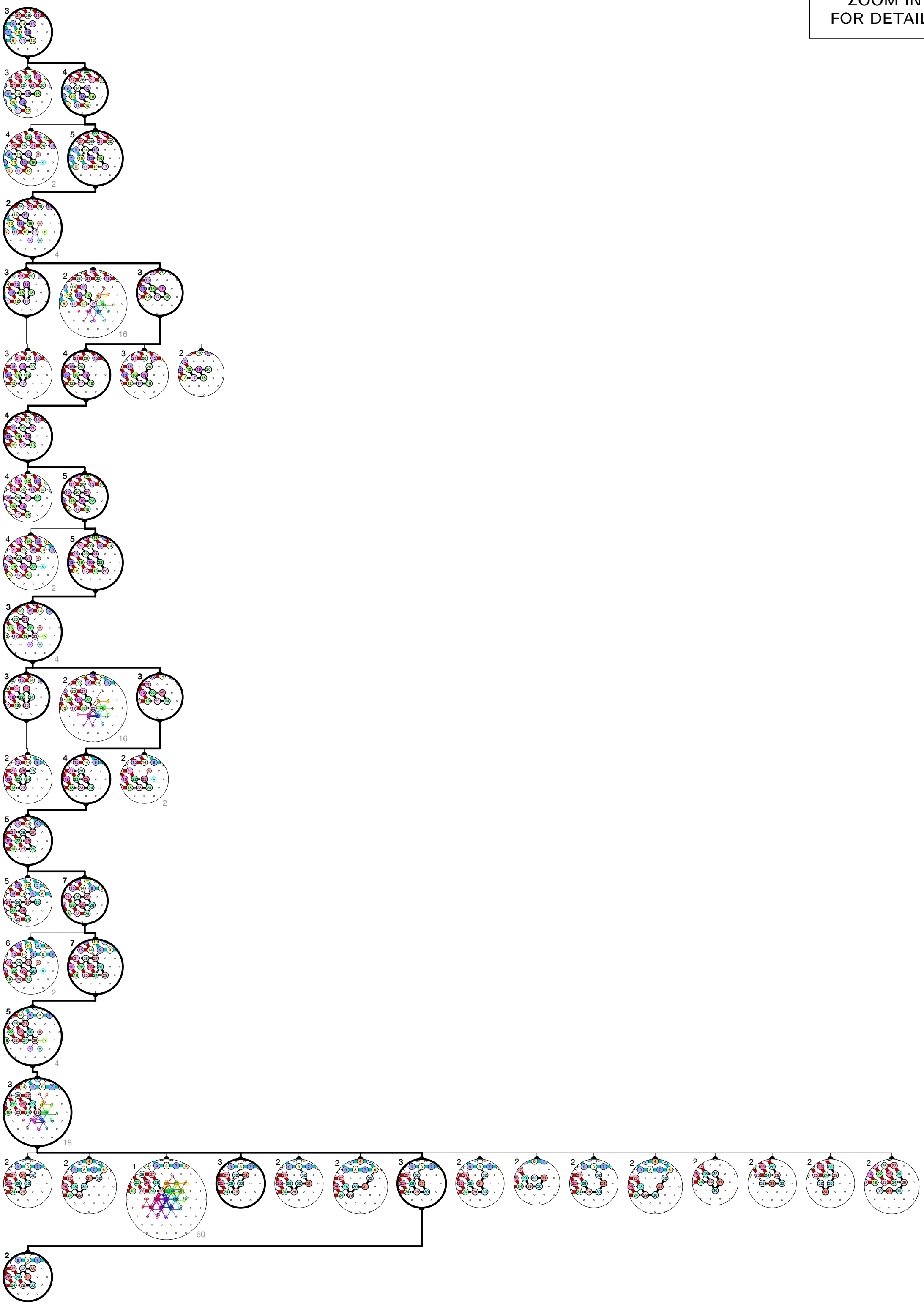

Output nascent configurations: λ'_2

Figure S.35. Folding of Brick B2 in 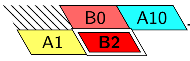

–First occurrence in 5-bits counter: Region #254.

Input nascent configurations: λ_4

ZOOM IN
FOR DETAILS

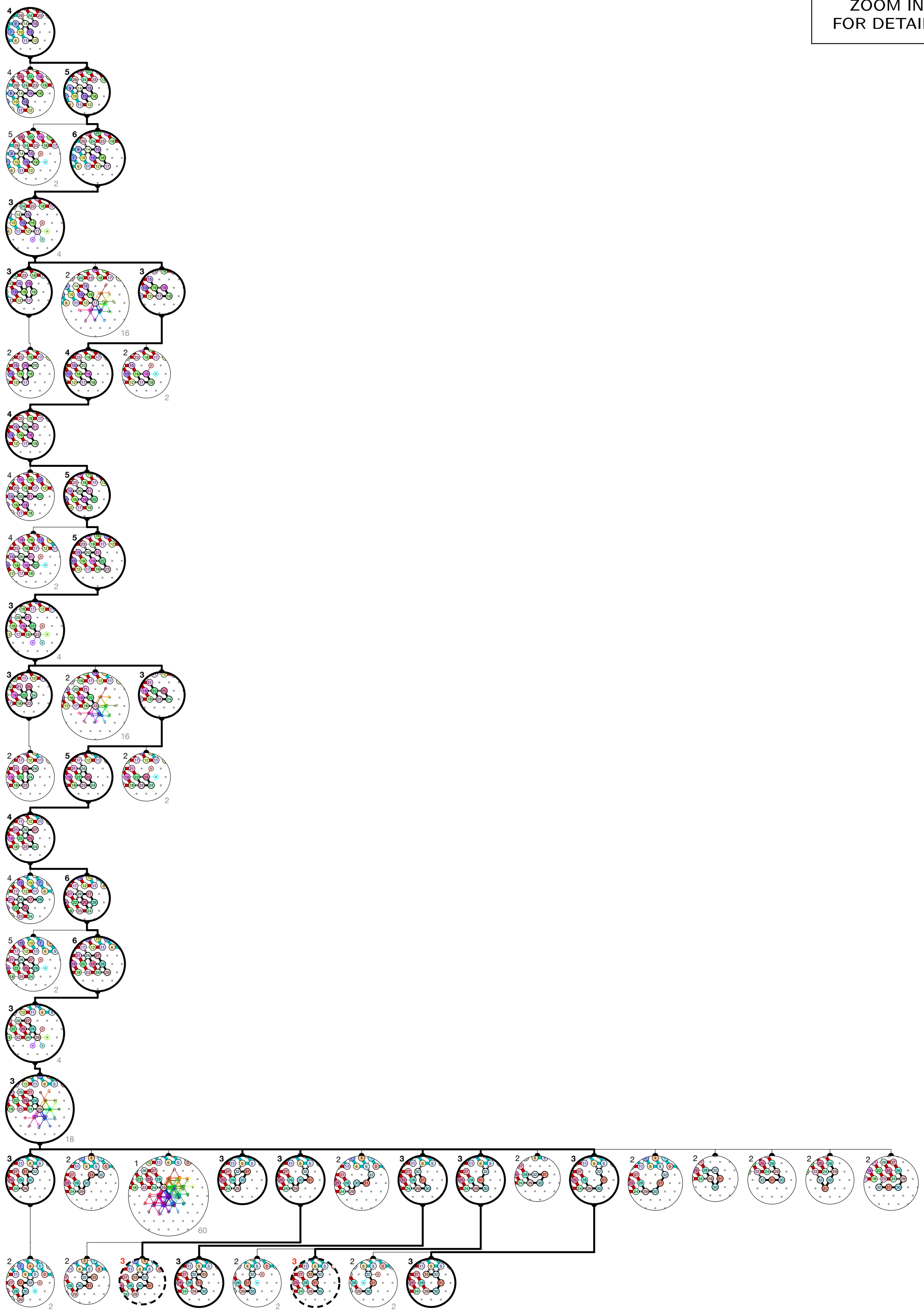

Output nascent configurations: $\mu \cup \mu'$

Figure S.36. Folding of Brick B2 in [B1 A11 A1 B2]–First occurrence in 5-bits counter: Region #38.

S.2.4. Folding certificates for Module C: Second Half-Adder

Input nascent configurations: $\theta \cup \theta'$

ZOOM IN
FOR DETAILS

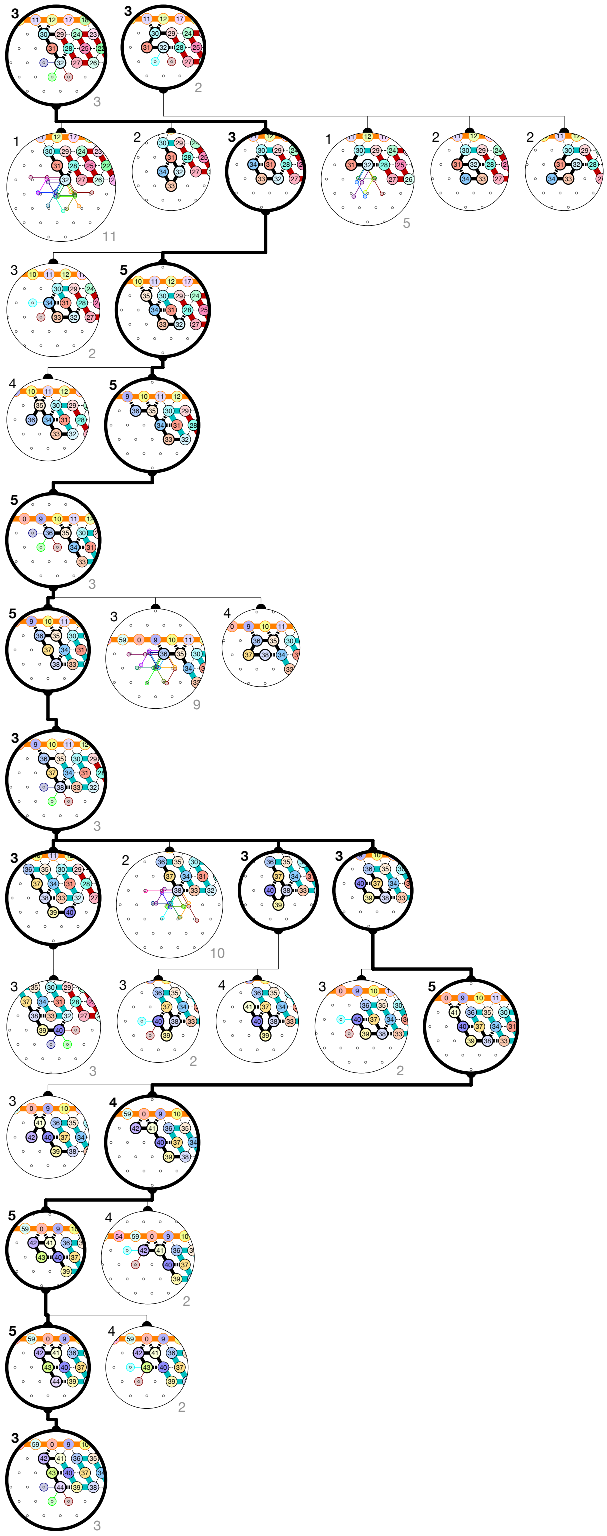

Output nascent configurations: β'

Figure S.37. Folding of Brick C00 in 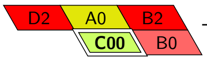

–First occurrence in 5-bits counter: Region #3.

Input nascent configurations: γ'

ZOOM IN
FOR DETAILS

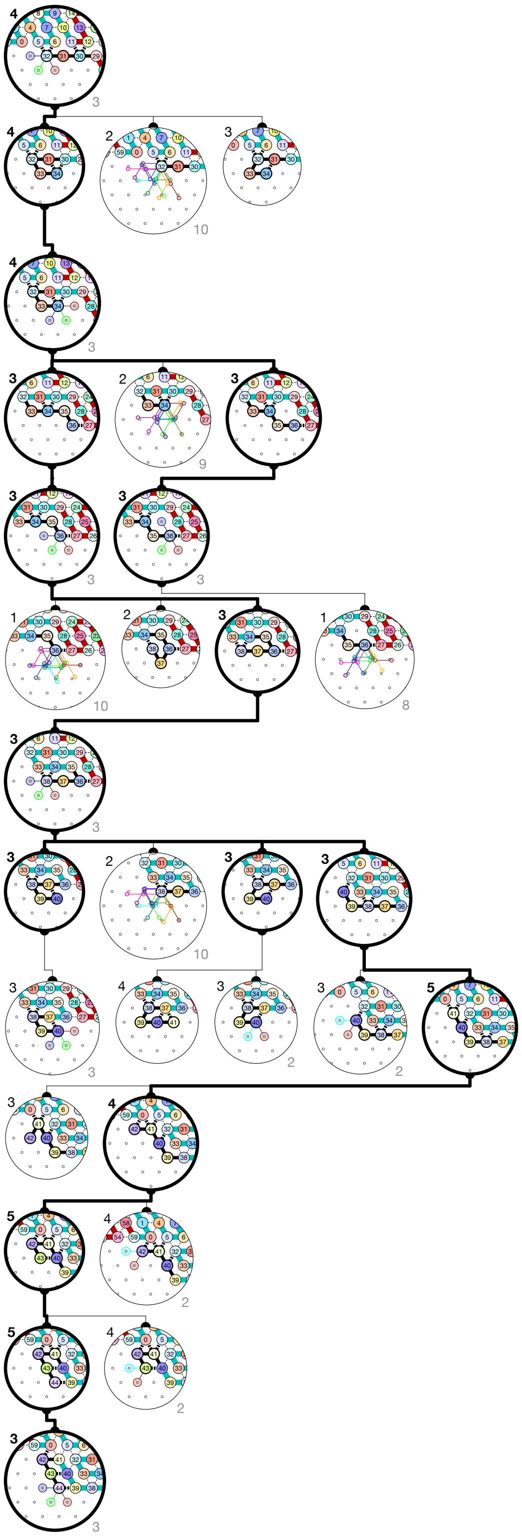

Output nascent configurations: β'

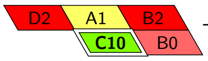

Figure S.38. Folding of Brick C10 in D2 A1 B2 C10 B0 –First occurrence in 5-bits counter: Region #43.

Input nascent configurations: α''

ZOOM IN
FOR DETAILS

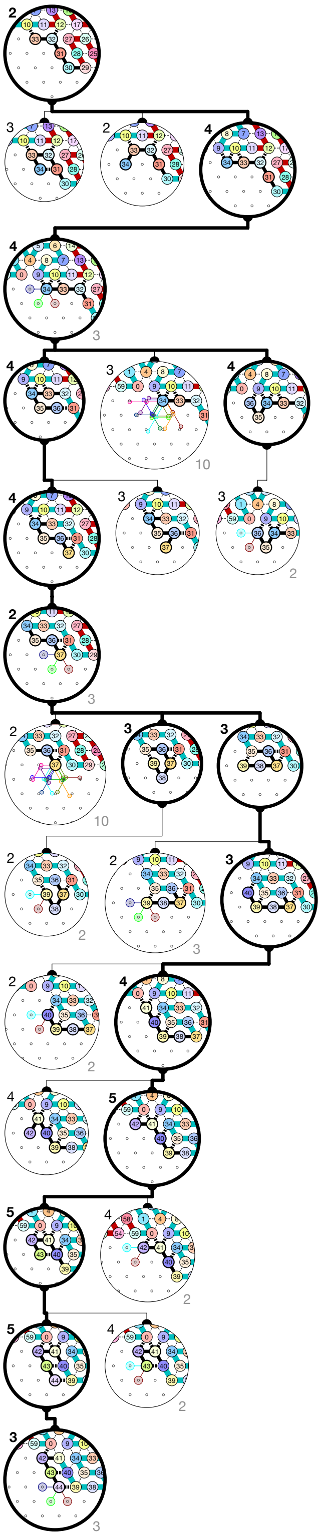

Output nascent configurations: β'

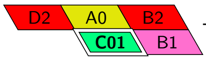

Figure S.39. Folding of Brick C01 in D2 A0 B2 C01 B1 –First occurrence in 5-bits counter: Region #23.

Input nascent configurations: α''

ZOOM IN
FOR DETAILS

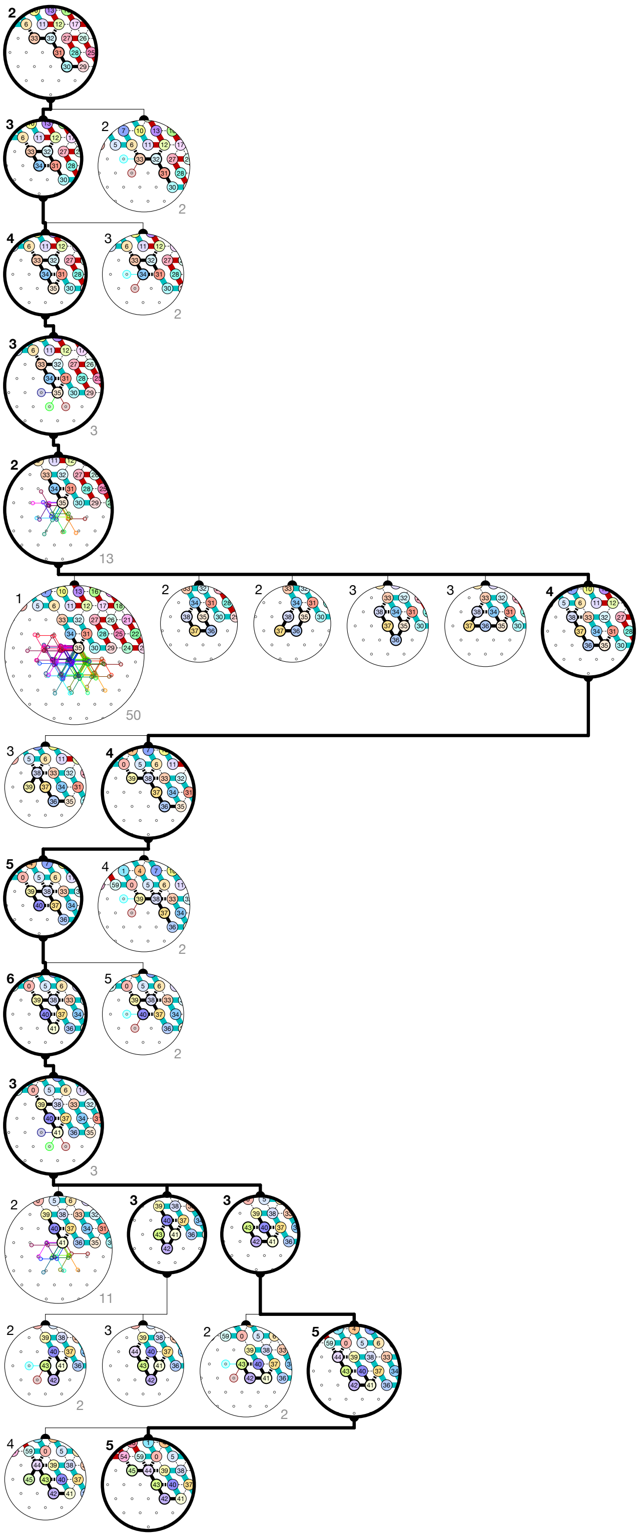

Output nascent configurations: α'''

Figure S.40. Folding of Brick C11 in 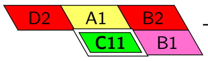 –First occurrence in 5-bits counter: Region #63.

Input nascent configurations: λ_2

ZOOM IN
FOR DETAILS

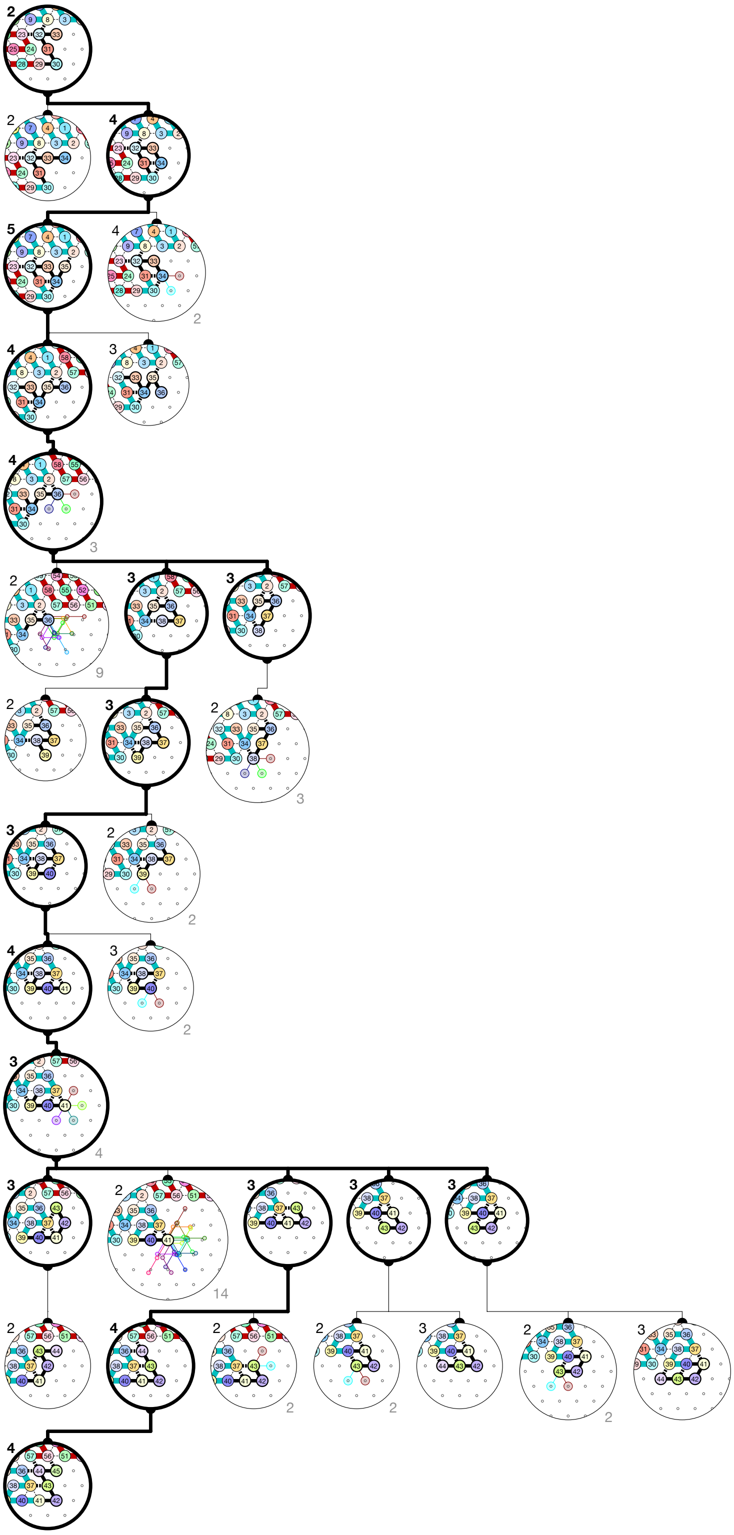

Output nascent configurations: λ'_4

Figure S.41. Folding of Brick C0 in 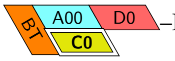

–First occurrence in 5-bits counter: Region #11.

Input nascent configurations: λ_2

ZOOM IN
FOR DETAILS

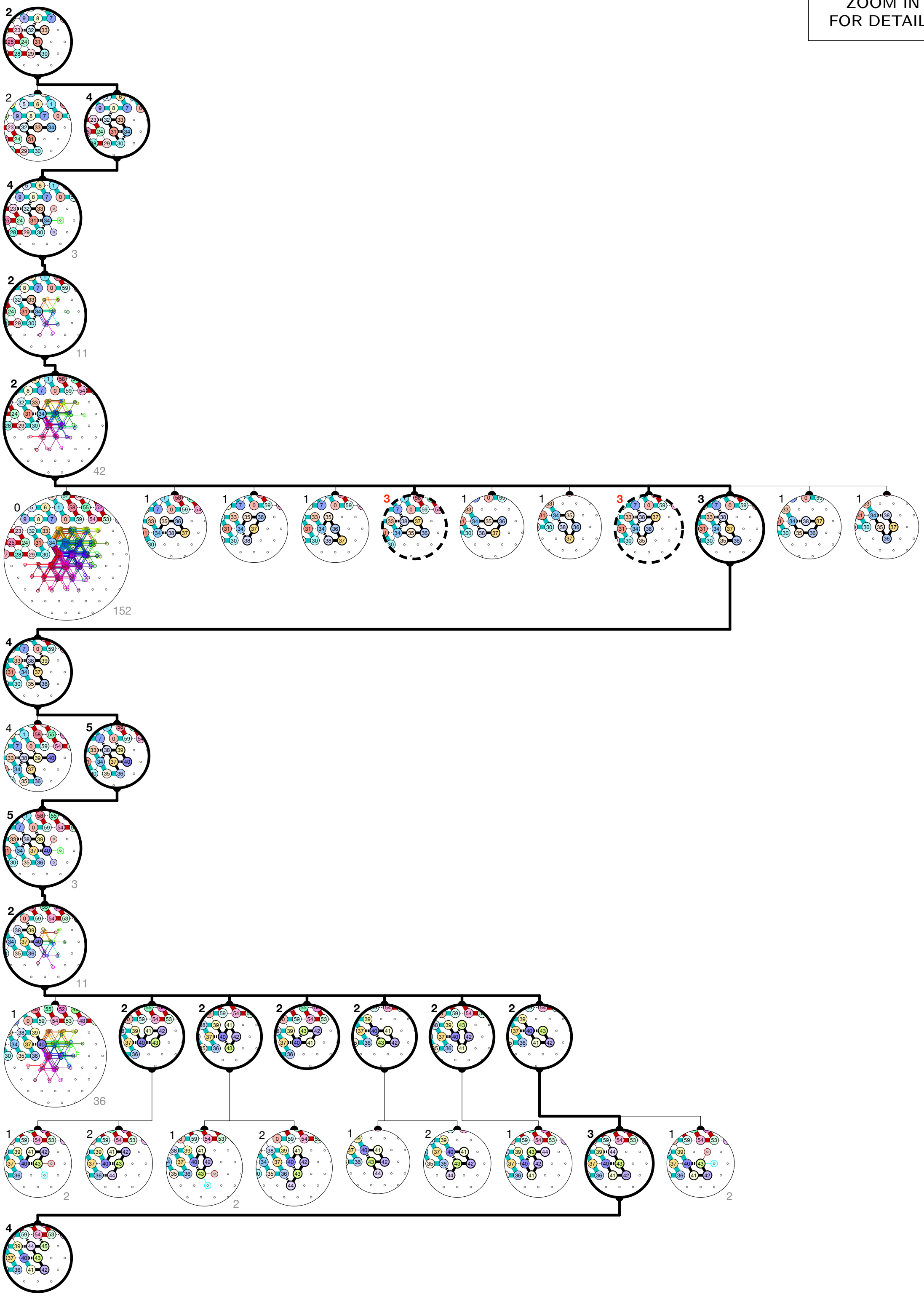

Output nascent configurations: λ'_4

Figure S.42. Folding of Brick C1 in 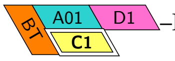–First occurrence in 5-bits counter: Region #311.

Input nascent configurations: λ_2

ZOOM IN
FOR DETAILS

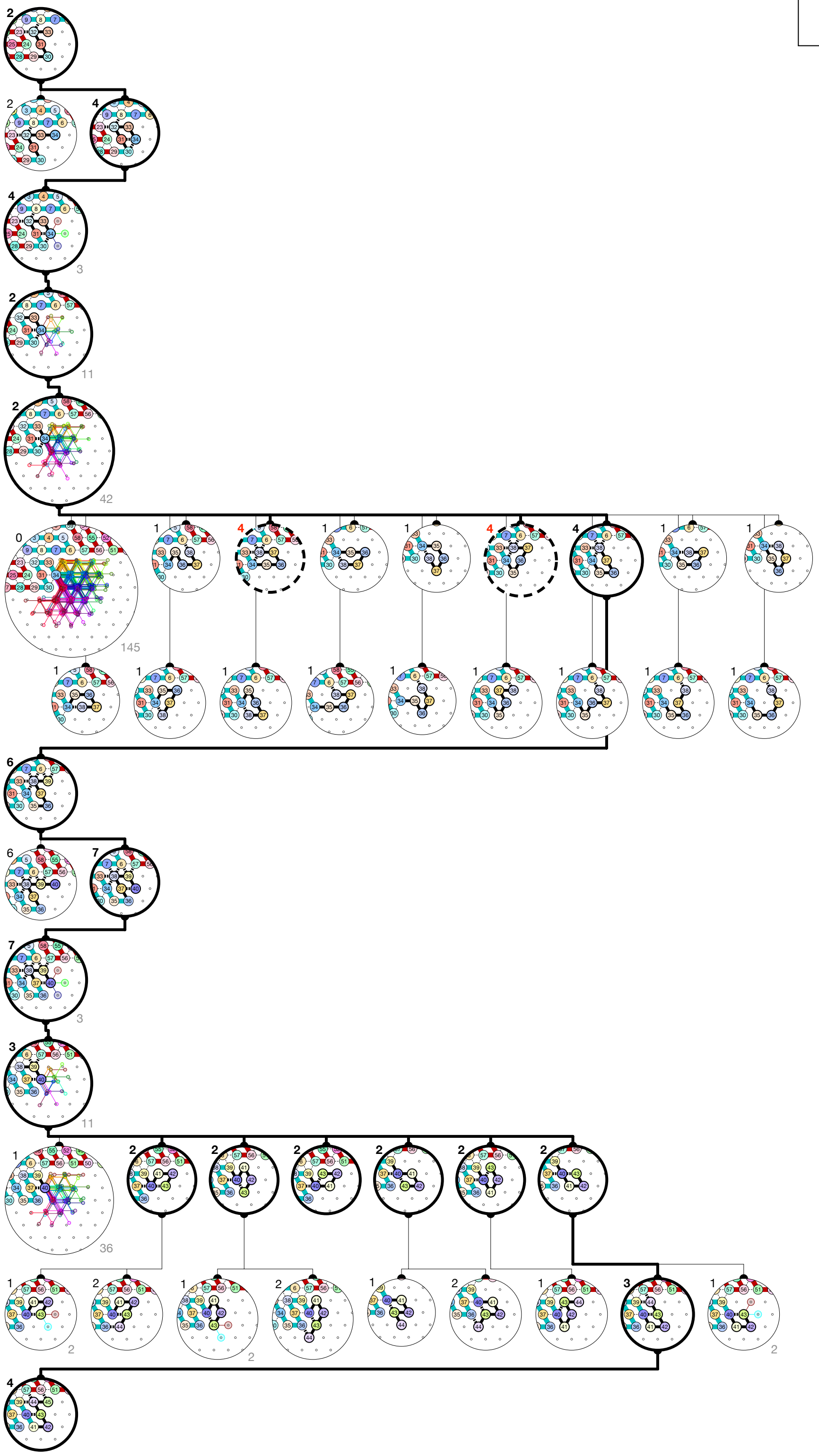

Output nascent configurations: λ'_4

Figure S.43. Folding of Brick C1 in 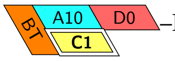 –First occurrence in 5-bits counter: Region #331.

Input nascent configurations: λ'_2

ZOOM IN
FOR DETAILS

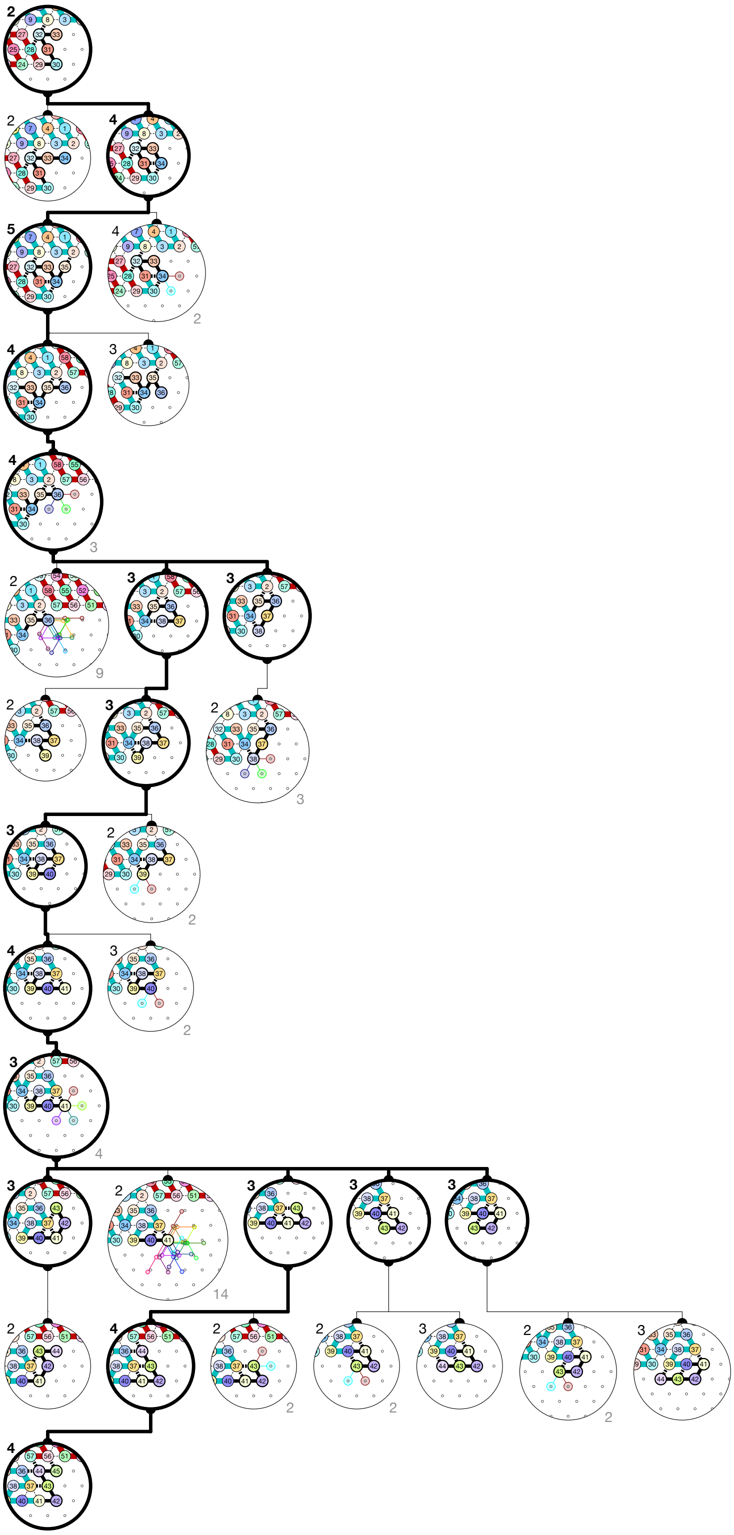

Output nascent configurations: λ'_4

Figure S.44. Folding of Brick C0 in 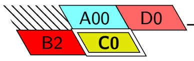

–First occurrence in 5-bits counter: Region #15.

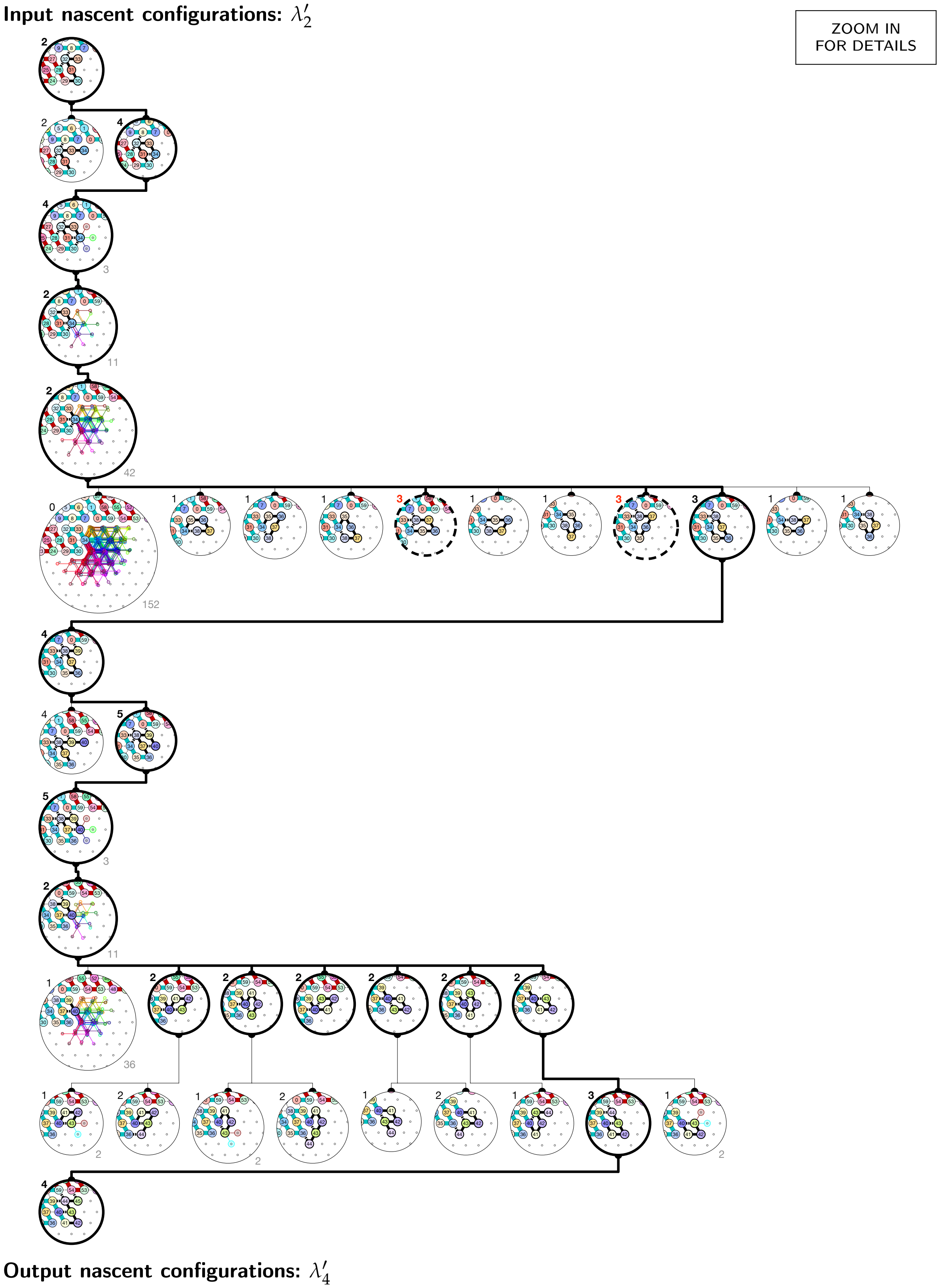

Figure S.45. Folding of Brick C1 in A01 D1 B2 C1 –First occurrence in 5-bits counter: Region #75.

Input nascent configurations: λ'_2

ZOOM IN
FOR DETAILS

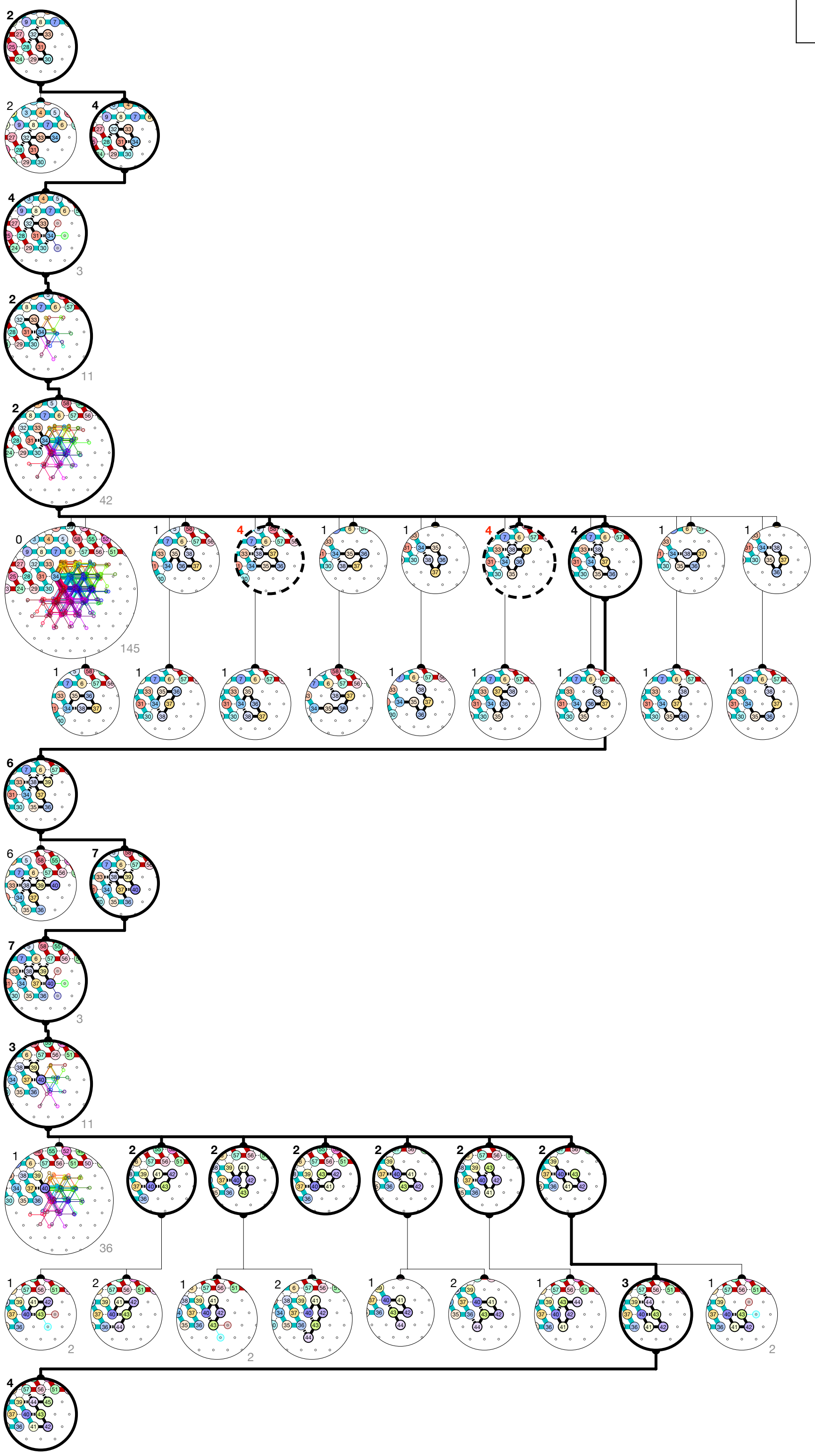

Output nascent configurations: λ'_4

Figure S.46. Folding of Brick C1 in 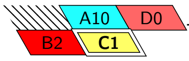

–First occurrence in 5-bits counter: Region #95.

Input nascent configurations: $\mu \cup \mu'$

ZOOM IN
FOR DETAILS

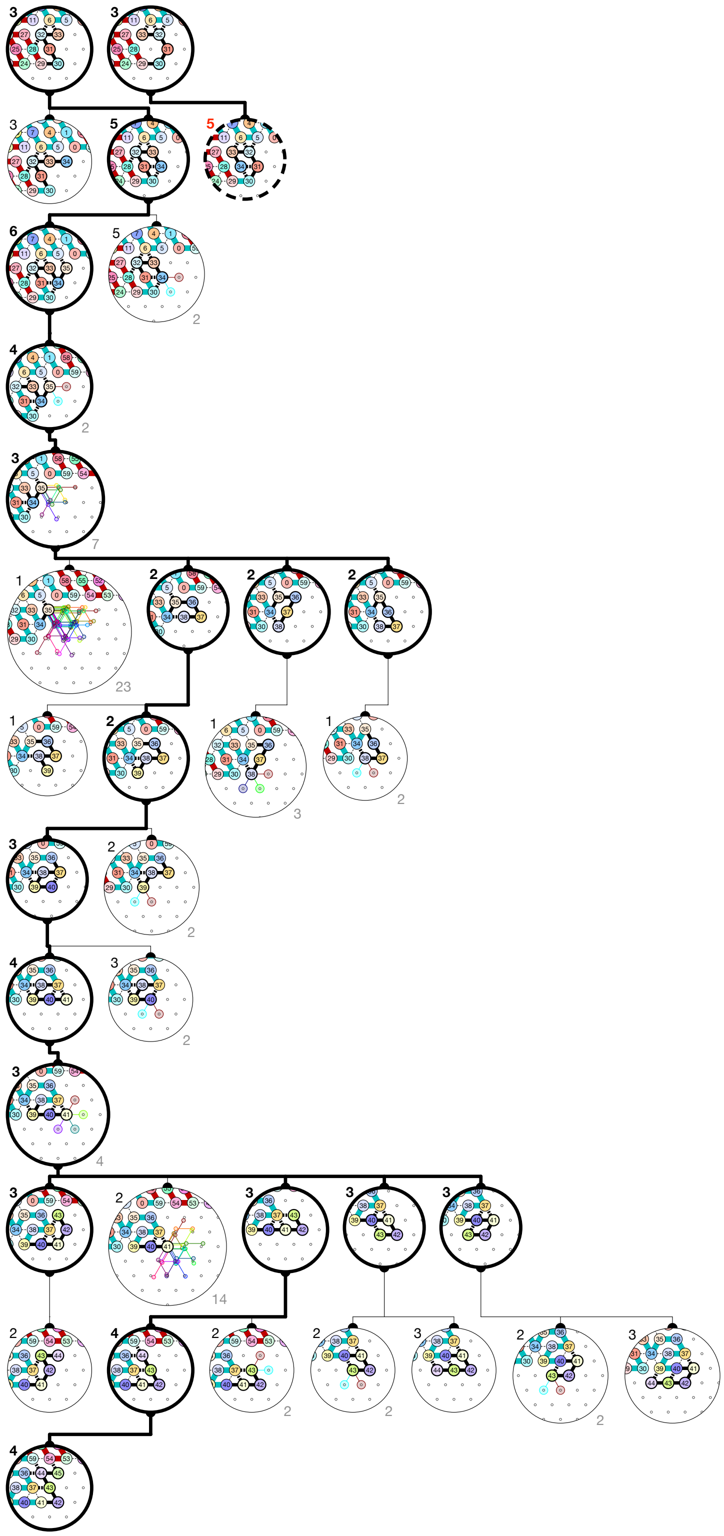

Output nascent configurations: λ'_4

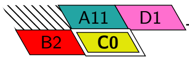

Figure S.47. Folding of Brick C0 in –First occurrence in 5-bits counter: Region #155.

Input nascent configurations: λ_2'

ZOOM IN
FOR DETAILS

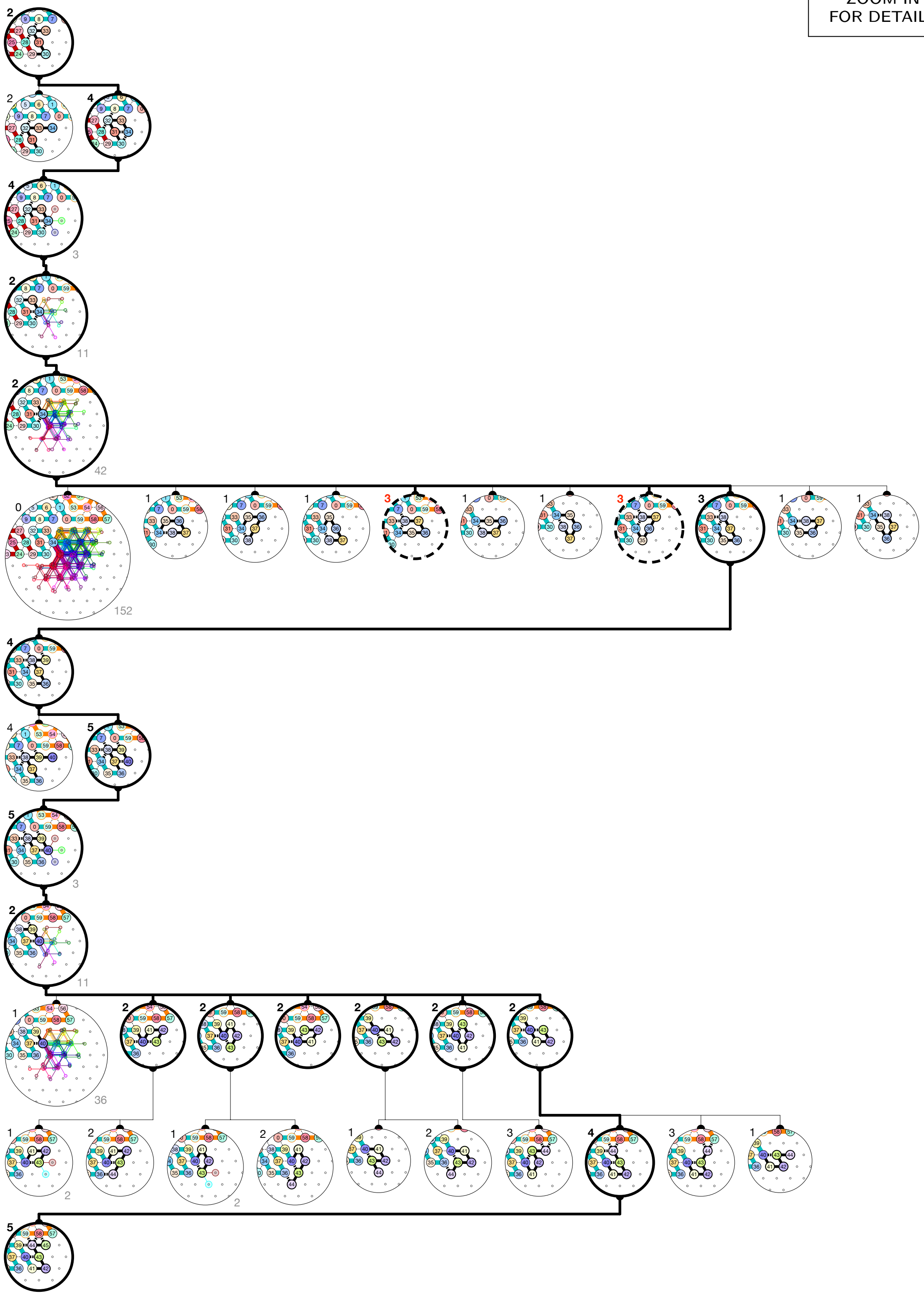

Output nascent configurations: λ_5

Figure S.48. Folding of Brick C1 in [DT, A01, B2, C1] –First occurrence in 5-bits counter: Region #19.

Input nascent configurations: $\mu \cup \mu'$

ZOOM IN
FOR DETAILS

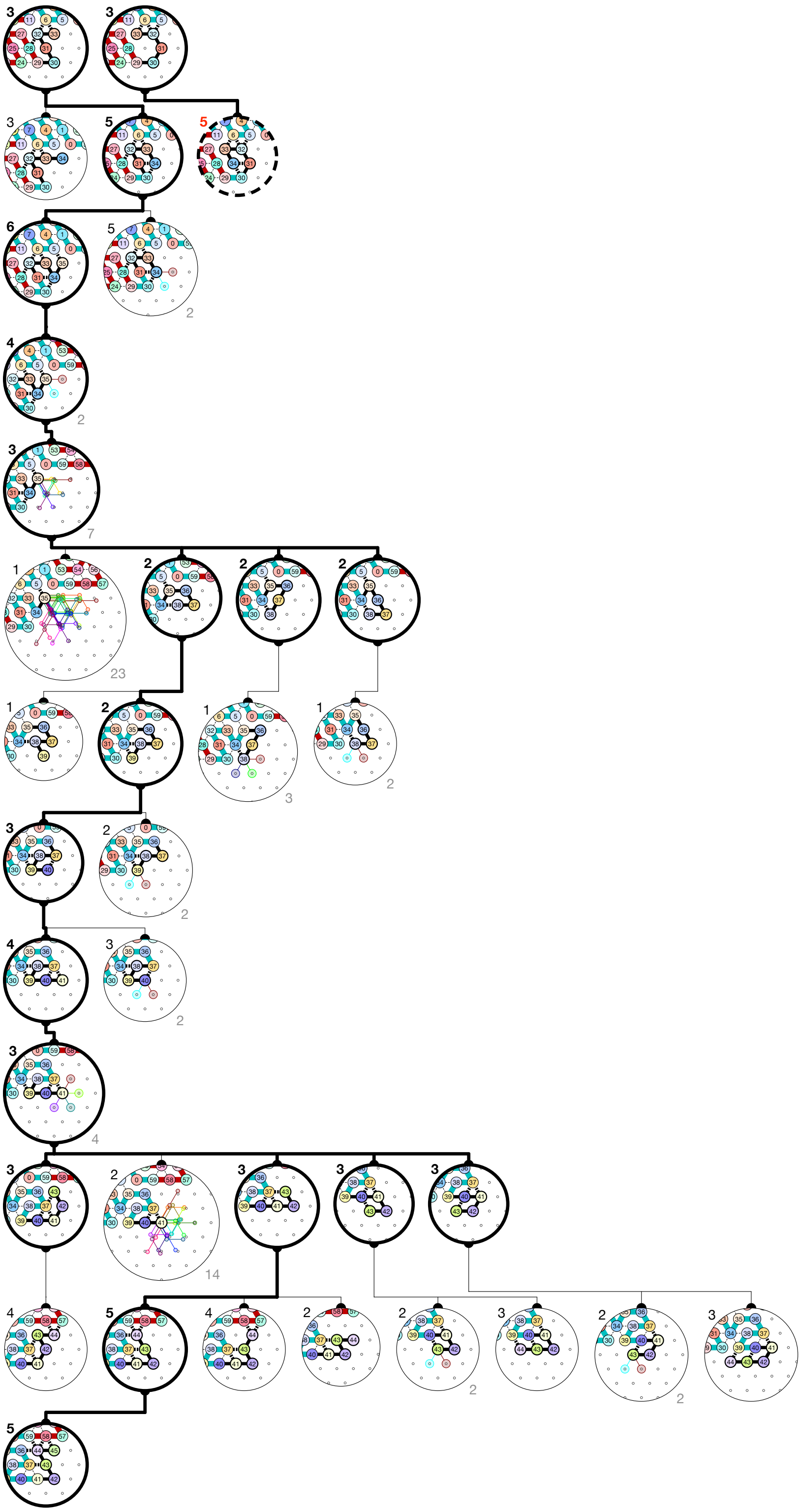

Output nascent configurations: λ_5

Figure S.49. Folding of Brick C0 in 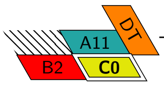

–First occurrence in 5-bits counter: Region #39.

S.2.5. Folding Certificates for Module D: Right Turn

Input nascent configurations: β'

ZOOM IN
FOR DETAILS

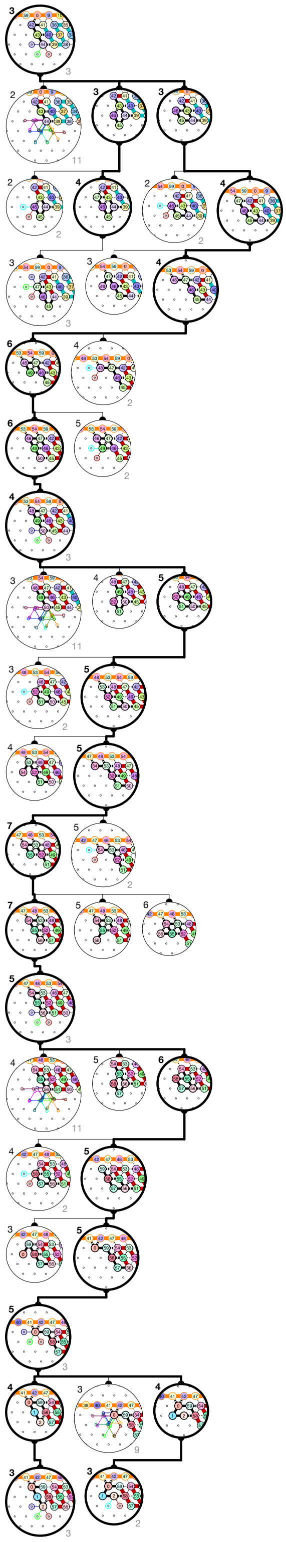

Output nascent configurations: $\theta \cup \theta'$

Figure S.50. Folding of Brick D0 in C0 D2 A0 D0 C00 –First occurrence in 5-bits counter: Region #4.

Input nascent configurations: β'

ZOOM IN
FOR DETAILS

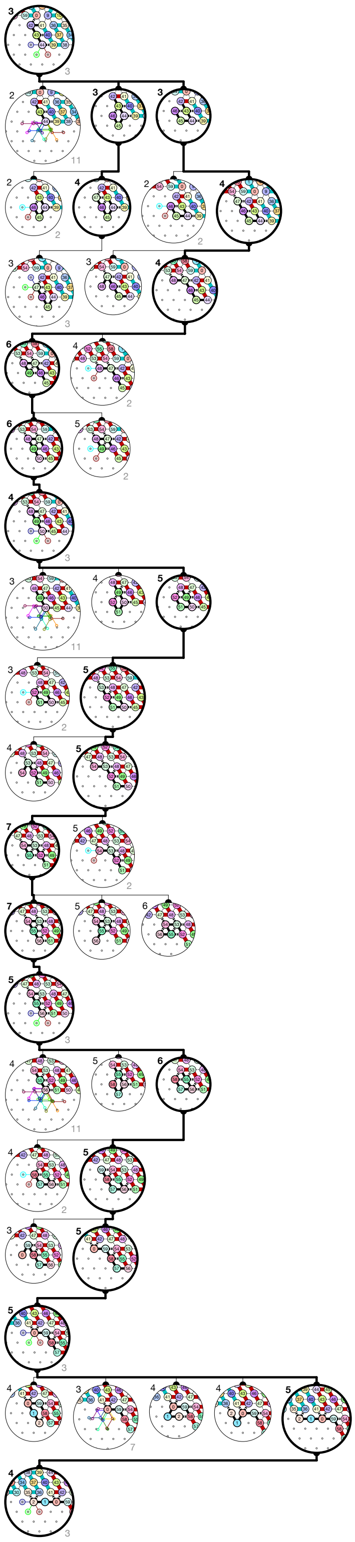

Output nascent configurations: γ'

Figure S.51. Folding of Brick D0 in 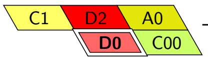

–First occurrence in 5-bits counter: Region #84.

Input nascent configurations: β'

ZOOM IN
FOR DETAILS

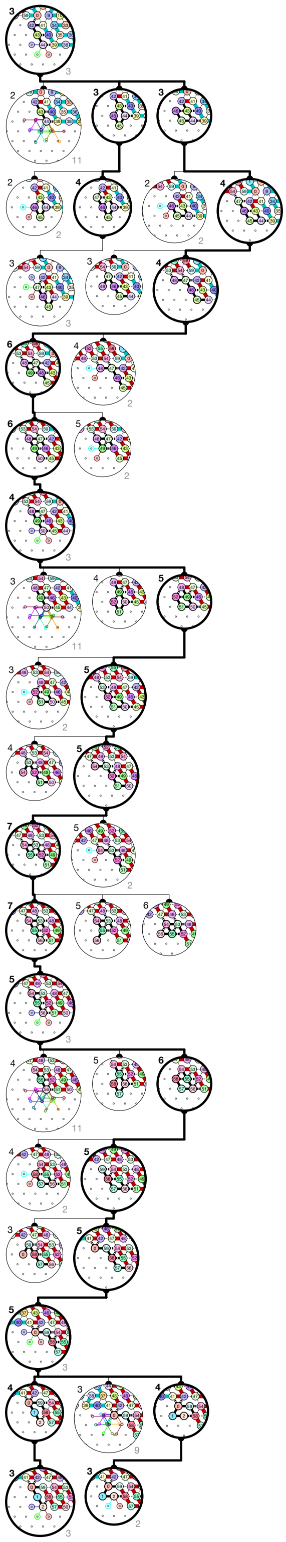

Output nascent configurations: $\theta \cup \theta'$

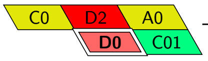

Figure S.52. Folding of Brick D0 in –First occurrence in 5-bits counter: Region #24.

Input nascent configurations: β'

ZOOM IN
FOR DETAILS

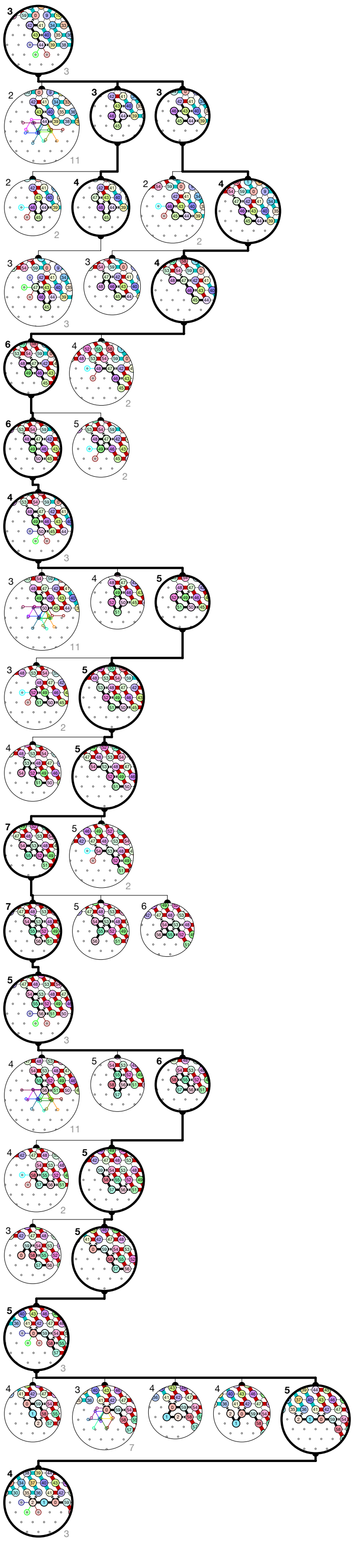

Output nascent configurations: γ'

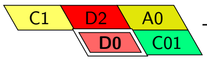

Figure S.53. Folding of Brick D0 in –First occurrence in 5-bits counter: Region #104.

Input nascent configurations: β'

ZOOM IN
FOR DETAILS

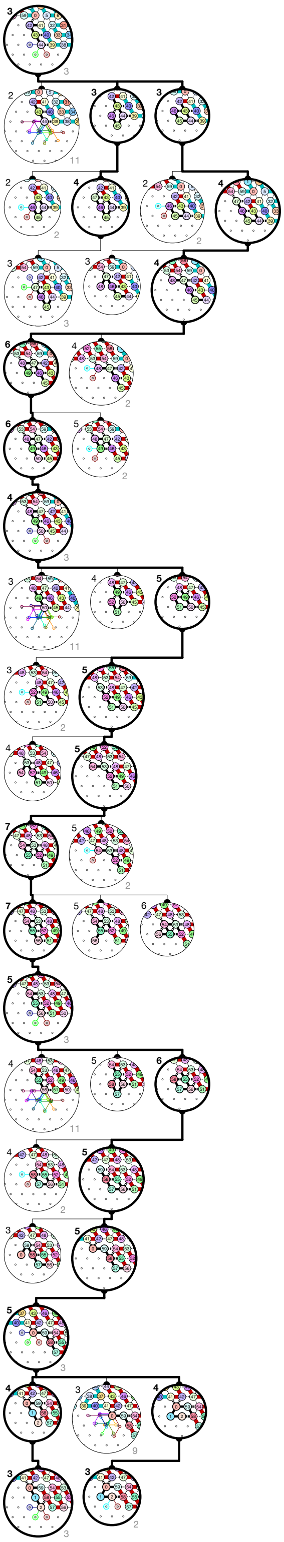

Output nascent configurations: $\theta \cup \theta'$

Figure S.54. Folding of Brick D0 in 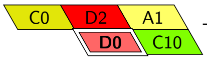 –First occurrence in 5-bits counter: Region #44.

Input nascent configurations: β'

ZOOM IN
FOR DETAILS

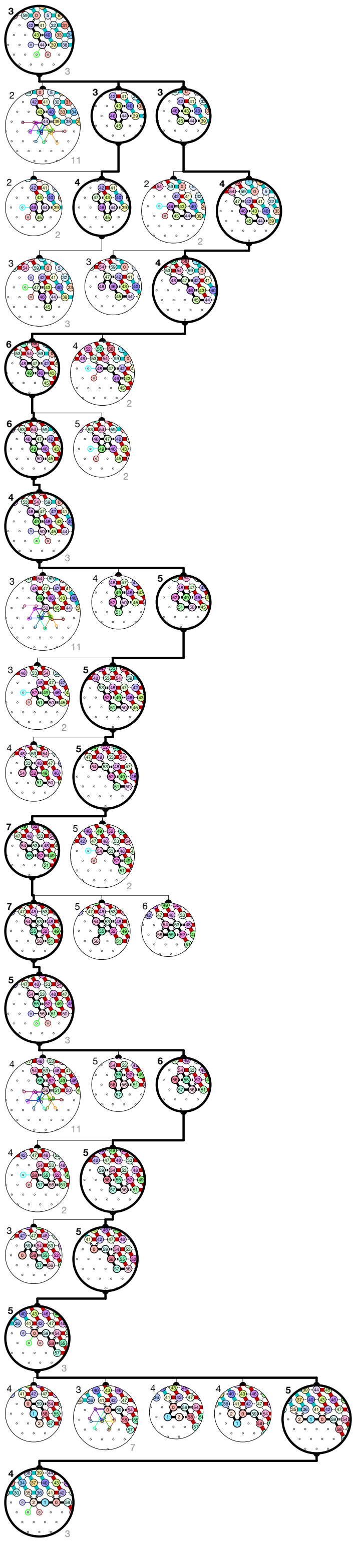

Output nascent configurations: γ'

Figure S.55. Folding of Brick D0 in

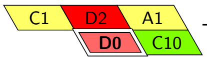

–First occurrence in 5-bits counter: Region #124.

Input nascent configurations: α'''

ZOOM IN FOR DETAILS

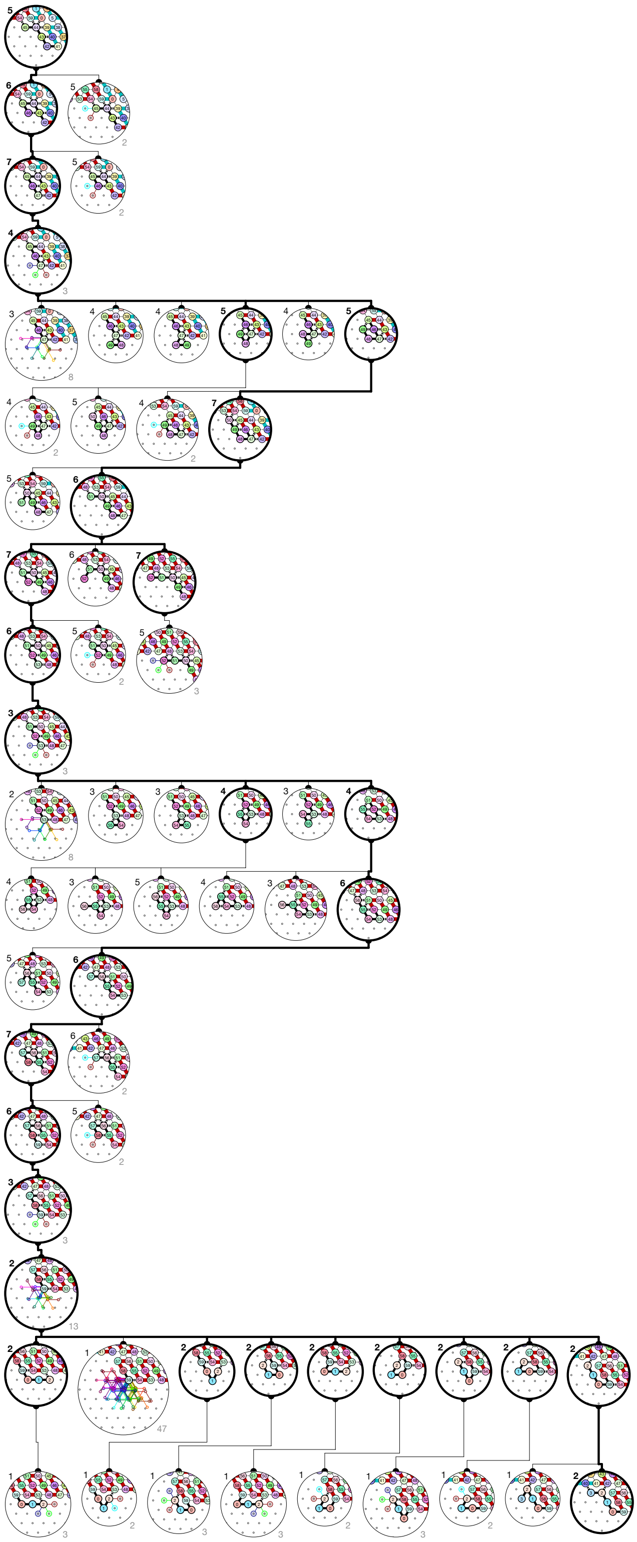

Output nascent configurations: α''

Figure S.56. Folding of Brick D1 in 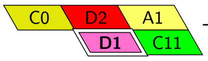

–First occurrence in 5-bits counter: Region #64.

Input nascent configurations: α'''

ZOOM IN
FOR DETAILS

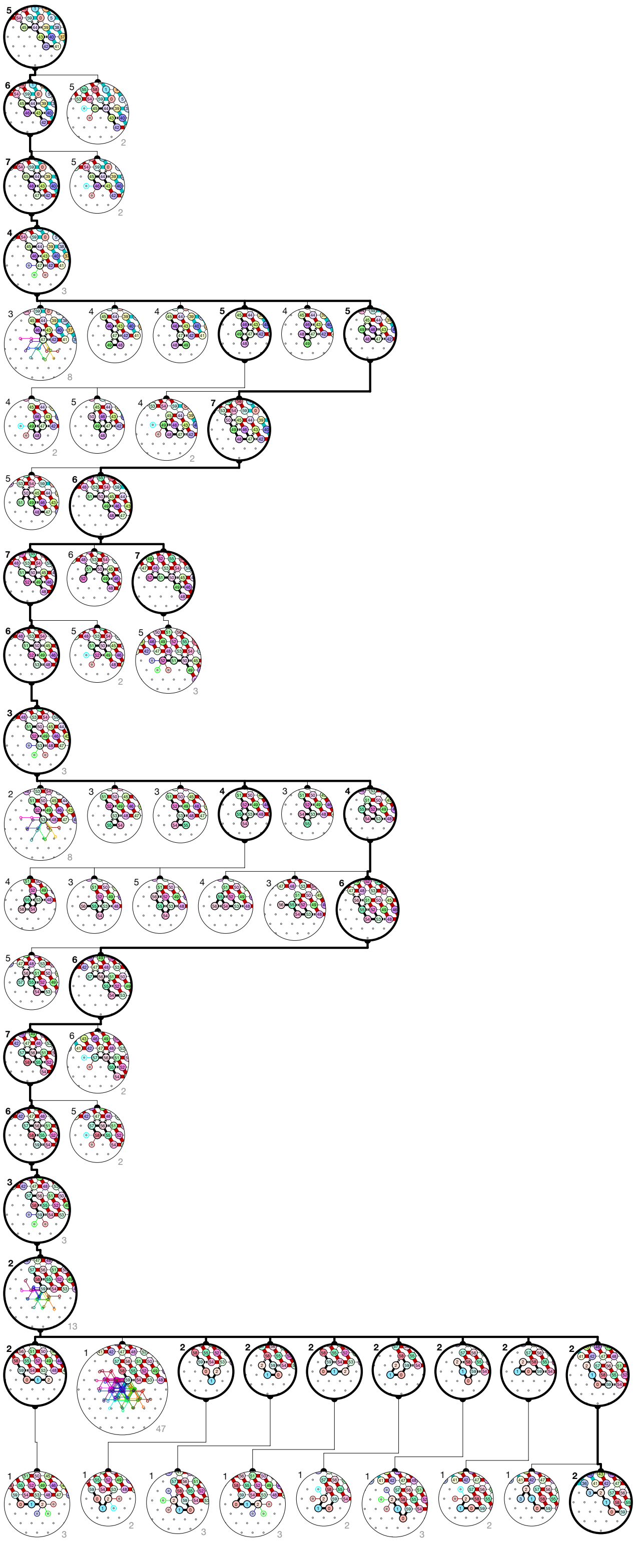

Output nascent configurations: α''

Figure S.57. Folding of Brick D1 in 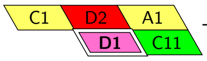 –First occurrence in 5-bits counter: Region #144.

Input nascent configurations: λ'_4

ZOOM IN
FOR DETAILS

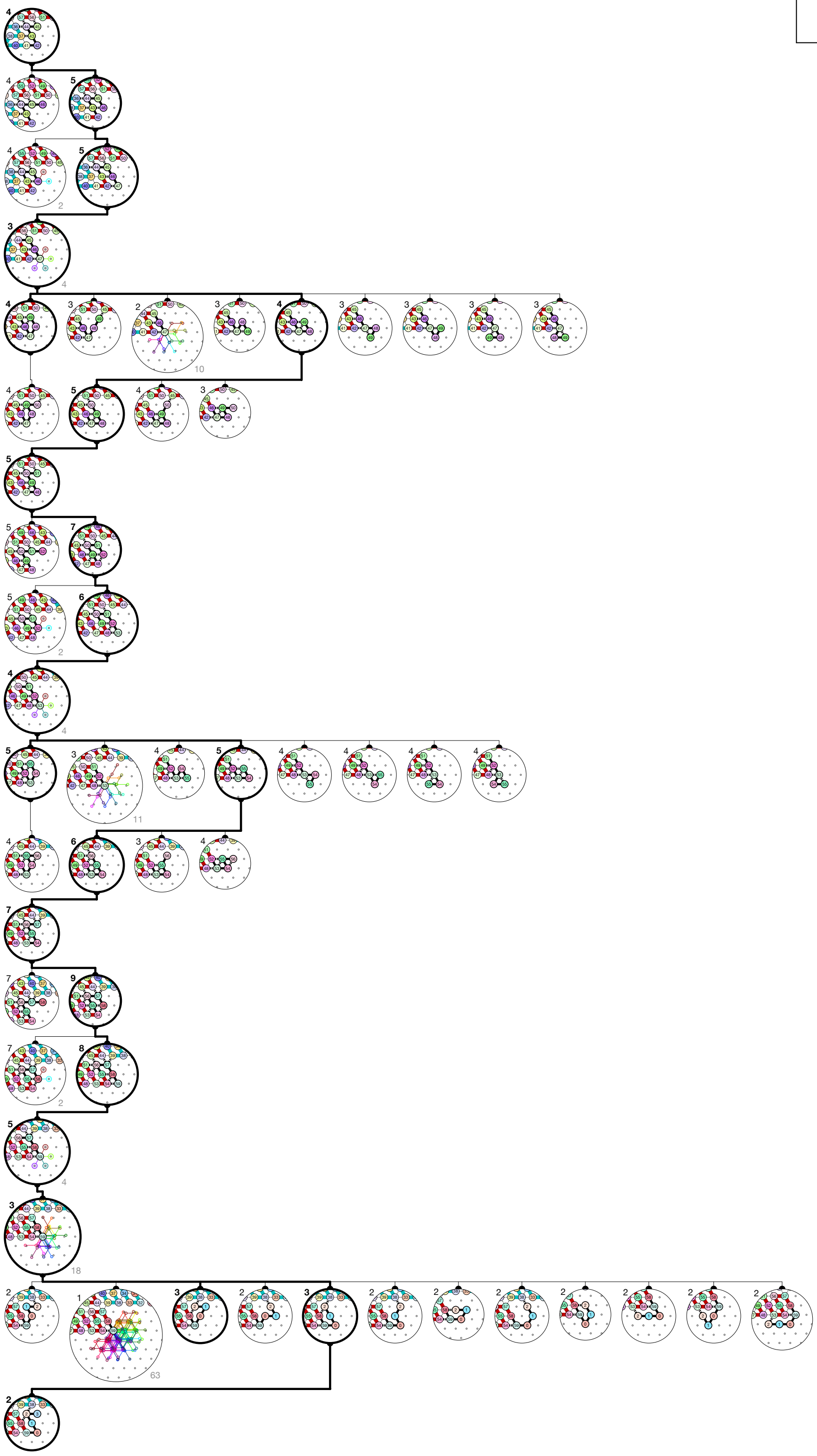

Output nascent configurations: λ'_2

Figure S.58. Folding of Brick D2 in 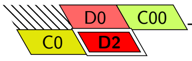

–First occurrence in 5-bits counter: Region #12.

Input nascent configurations: λ'_4

ZOOM IN
FOR DETAILS

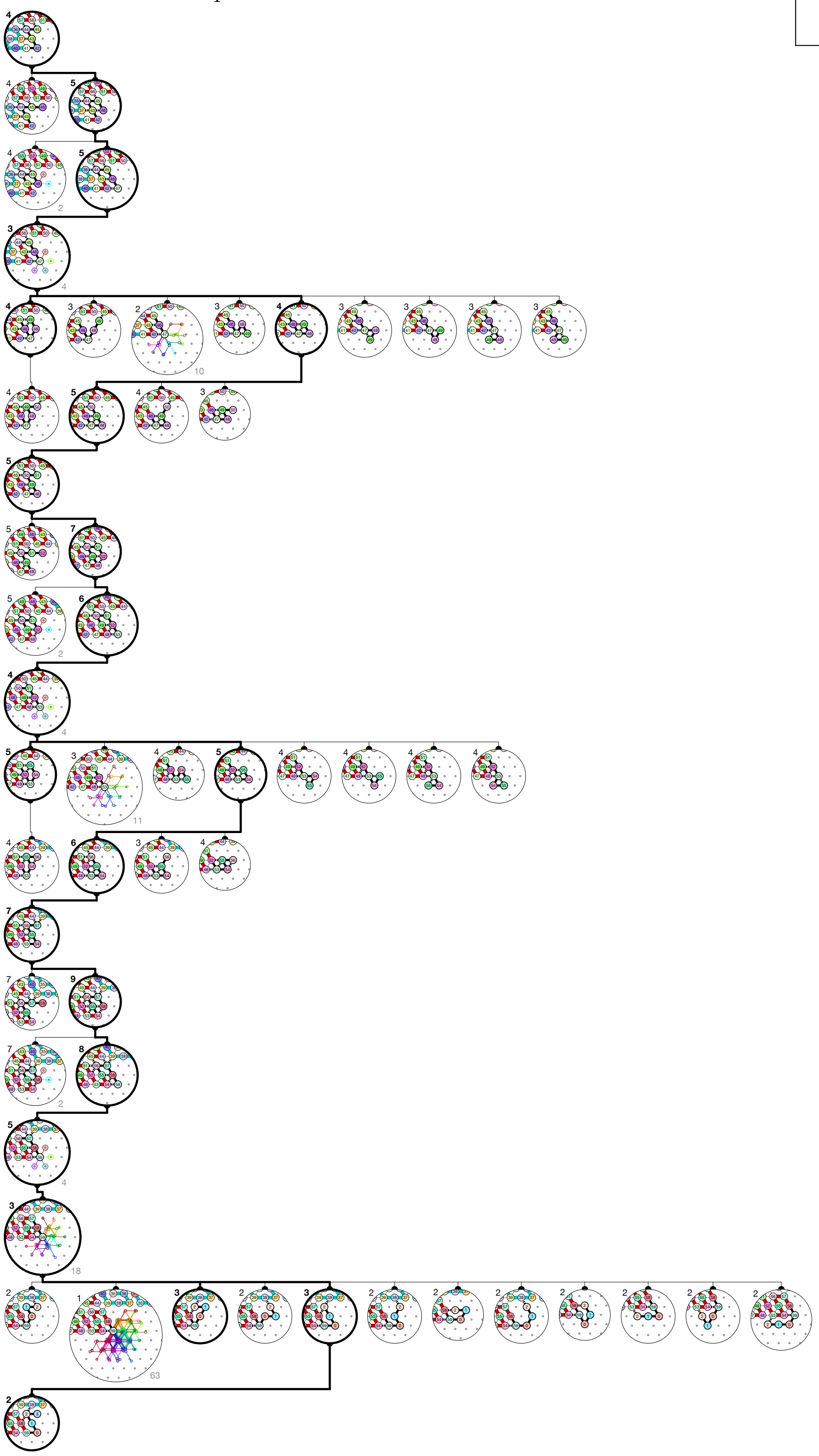

Output nascent configurations: λ'_2

Figure S.59. Folding of Brick D2 in 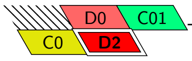

–First occurrence in 5-bits counter: Region #36.

Input nascent configurations: λ'_4

ZOOM IN
FOR DETAILS

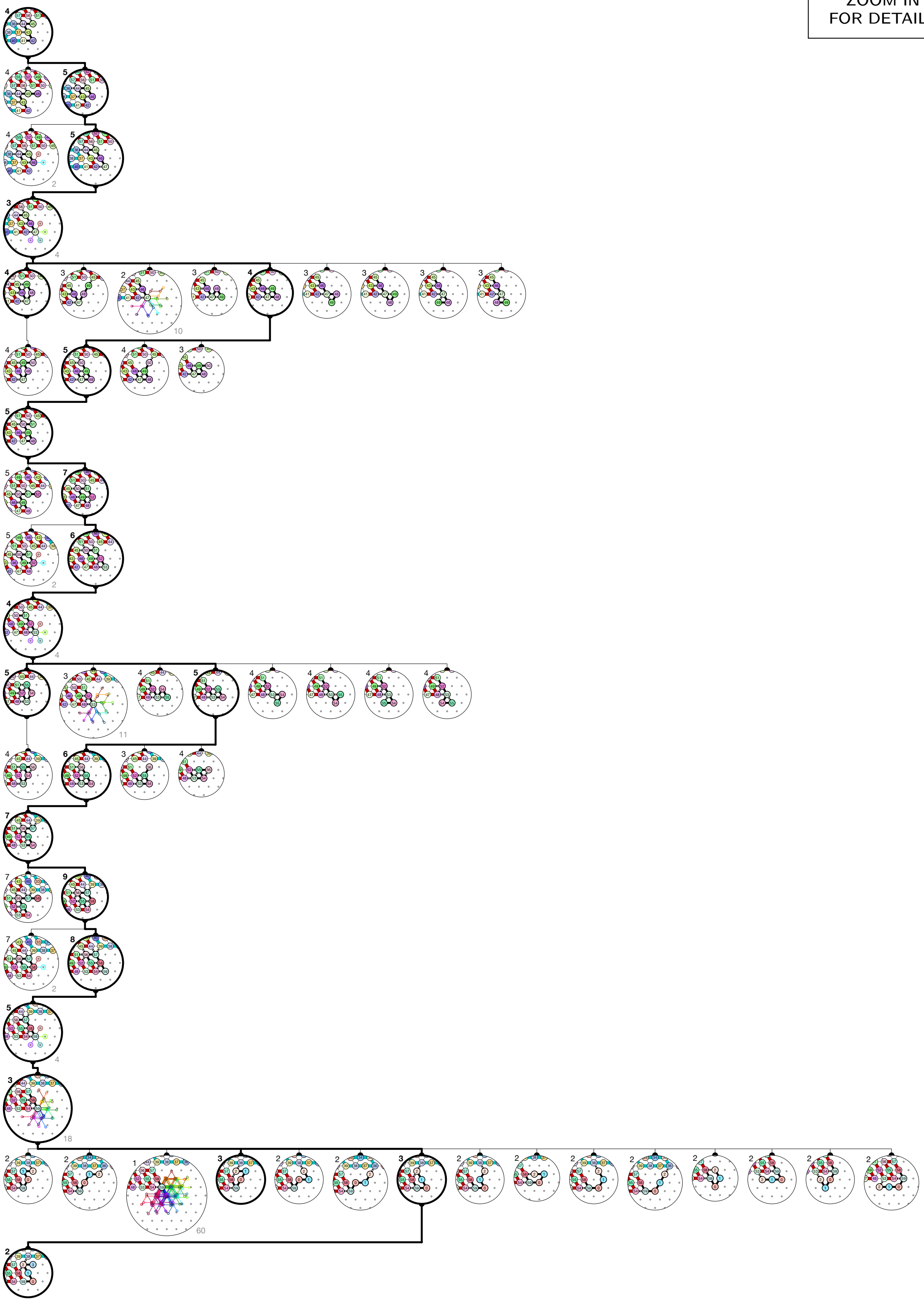

Output nascent configurations: λ'_2

Figure S.60. Folding of Brick D2 in 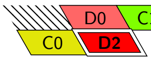

–First occurrence in 5-bits counter: Region #56.

Input nascent configurations: λ'_4

ZOOM IN FOR DETAILS

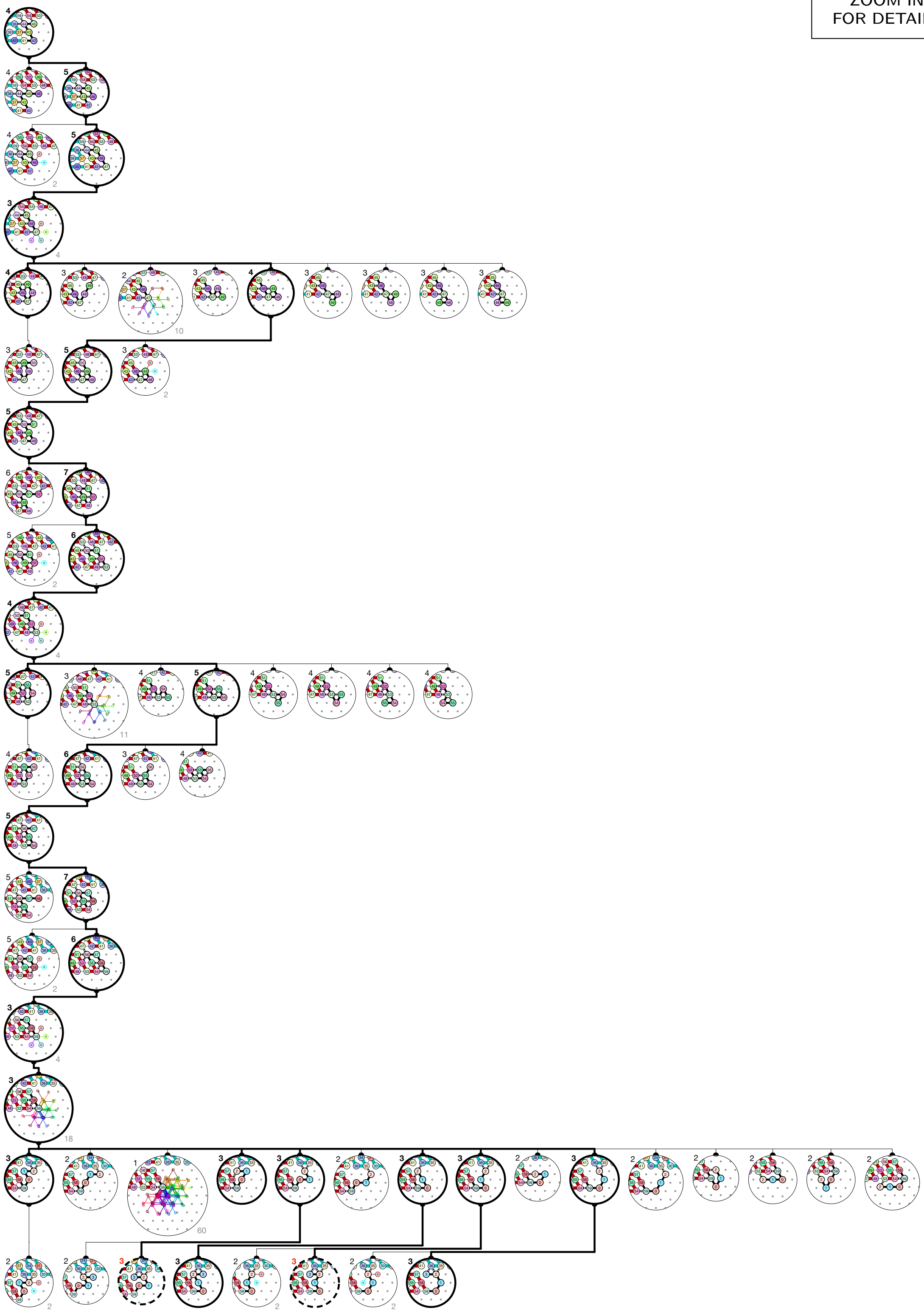

Output nascent configurations: $\mu \cup \mu'$

Figure S.61. Folding of Brick D2 in

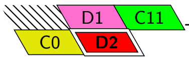

–First occurrence in 5-bits counter: Region #156.

ZOOM IN FOR DETAILS

Input nascent configurations: λ_4'

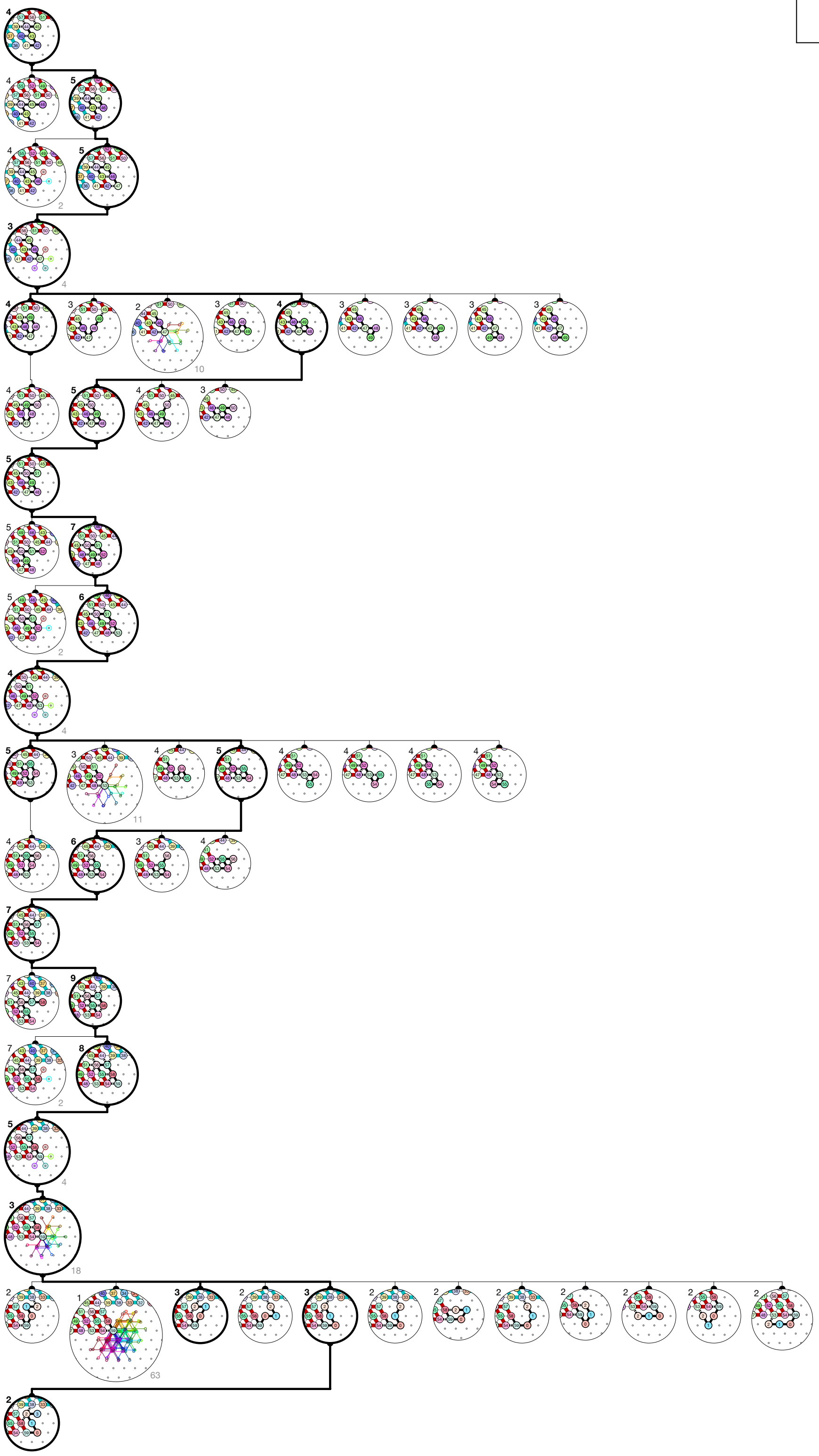

Output nascent configurations: λ_2'

Figure S.62. Folding of Brick D2 in 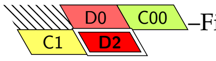

–First occurrence in 5-bits counter: Region #96.

Input nascent configurations: λ'_4

ZOOM IN
FOR DETAILS

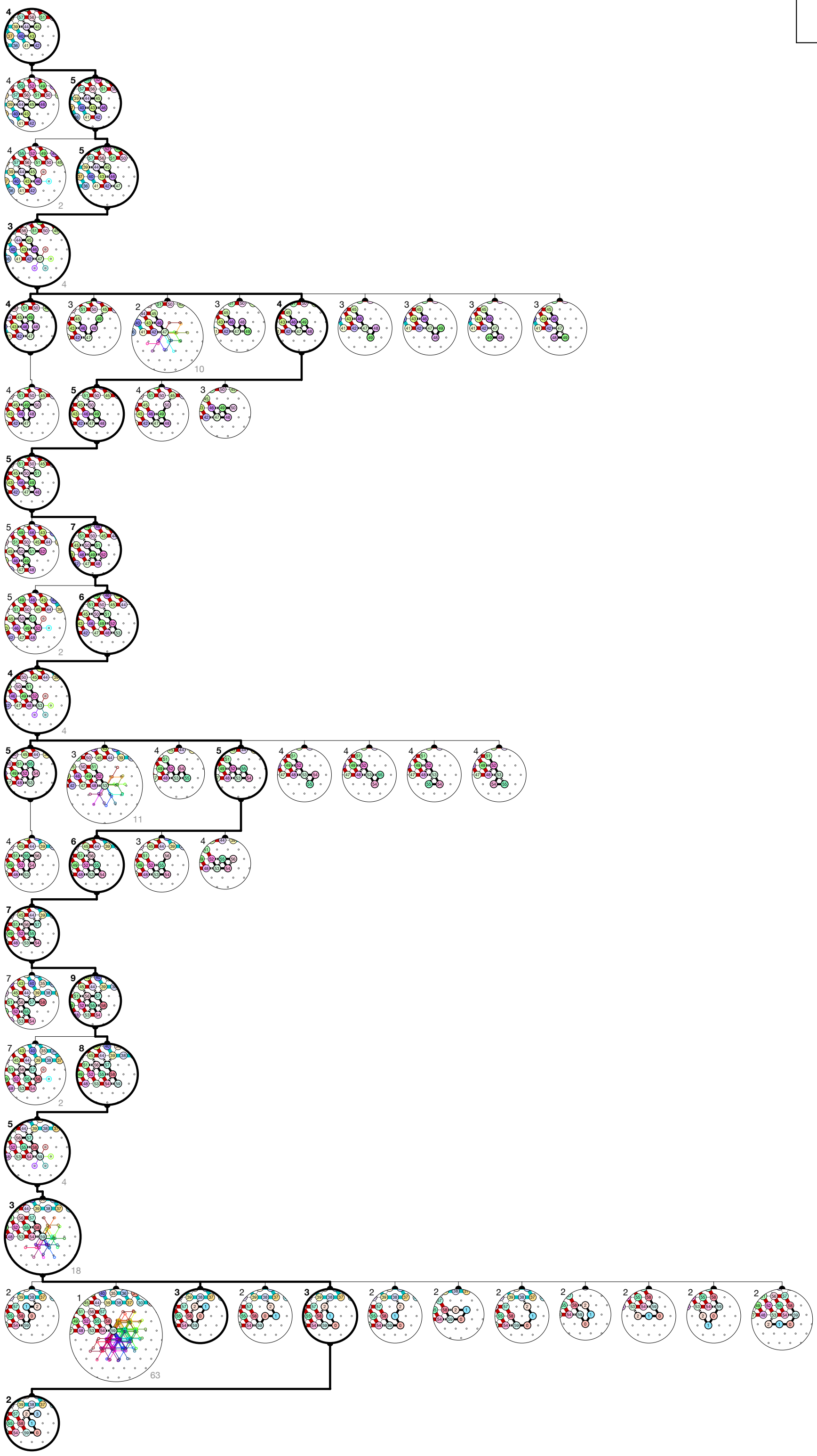

Output nascent configurations: λ'_2

Figure S.63. Folding of Brick D2 in 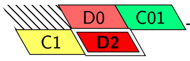 –First occurrence in 5-bits counter: Region #116.

Input nascent configurations: λ'_4

ZOOM IN
FOR DETAILS

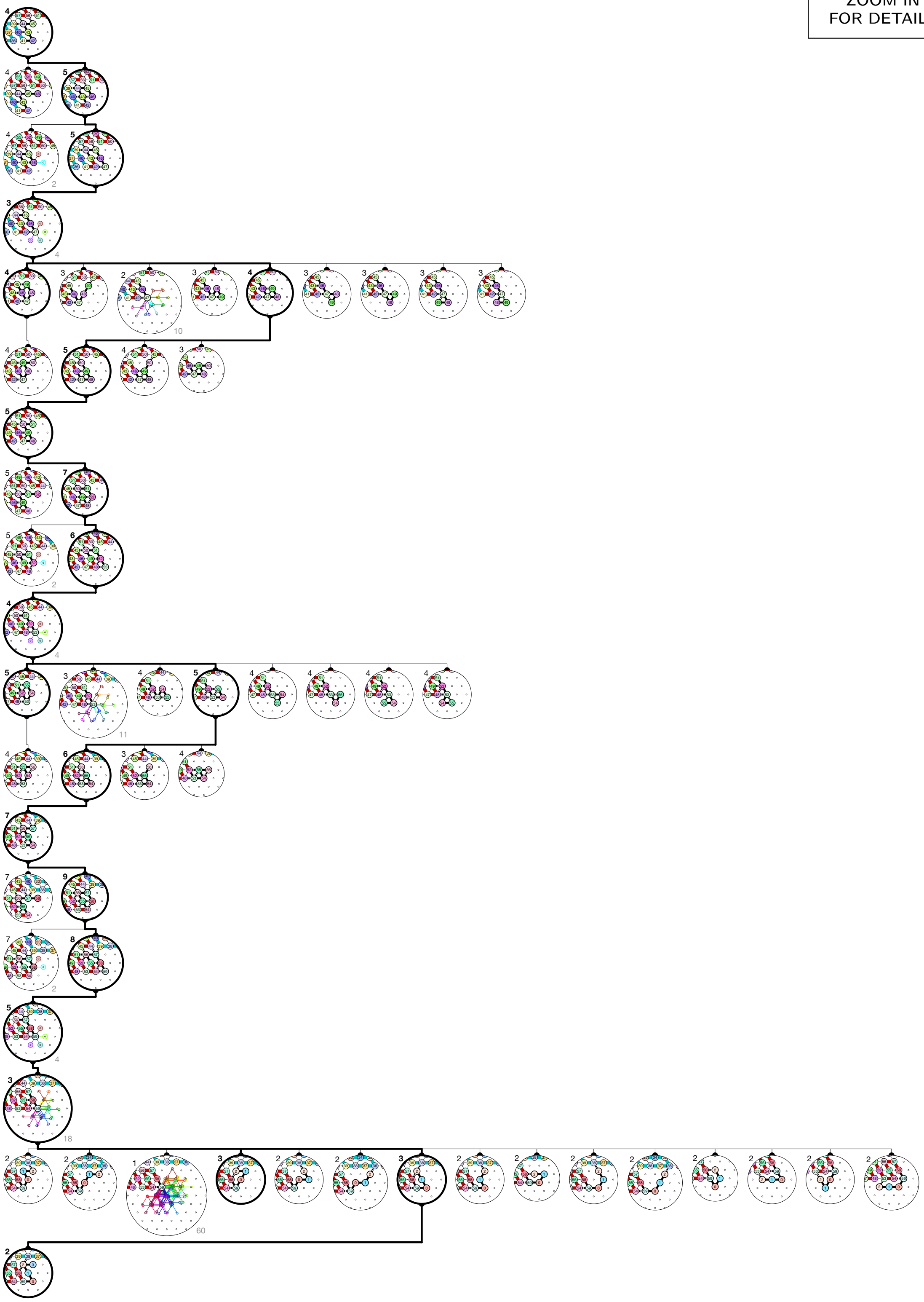

Output nascent configurations: λ'_2

Figure S.64. Folding of Brick D2 in 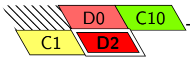 –First occurrence in 5-bits counter: Region #136.

Input nascent configurations: λ'_4

ZOOM IN
FOR DETAILS

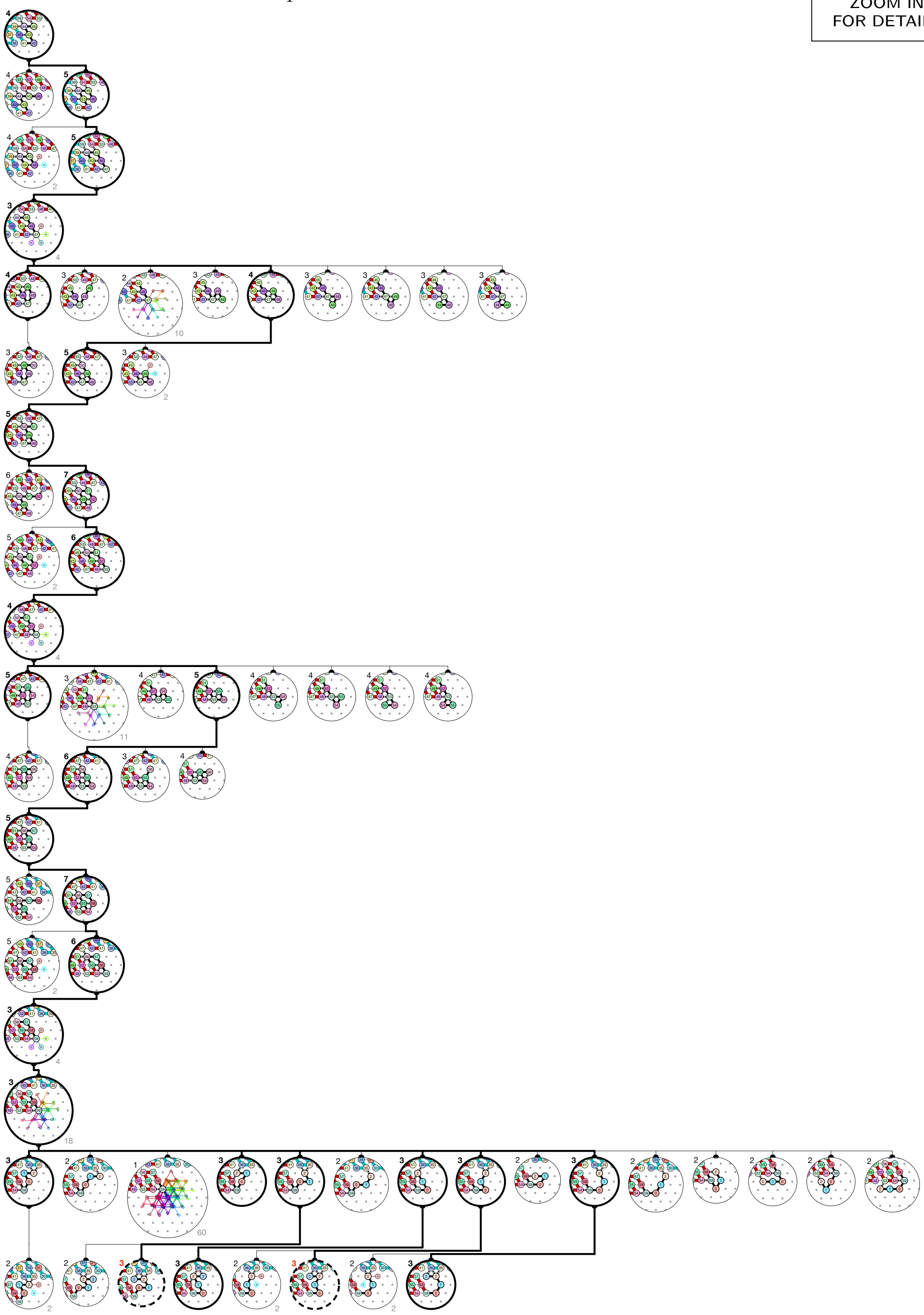

Output nascent configurations: $\mu \cup \mu'$

Figure S.65. Folding of Brick D2 in 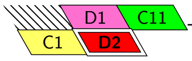

–First occurrence in 5-bits counter: Region #76.

Input nascent configurations: λ_5

ZOOM IN
FOR DETAILS

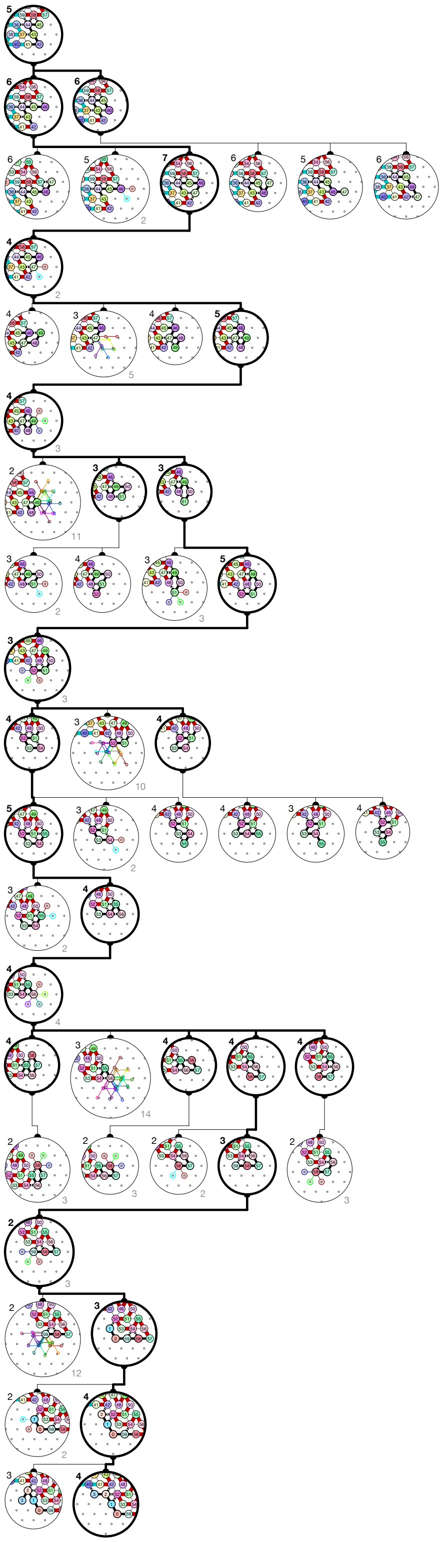

Output nascent configurations: α

Figure S.66. Folding of Brick DT in 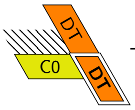

–First occurrence in 5-bits counter: Region #40.

Input nascent configurations: λ_5

ZOOM IN
FOR DETAILS

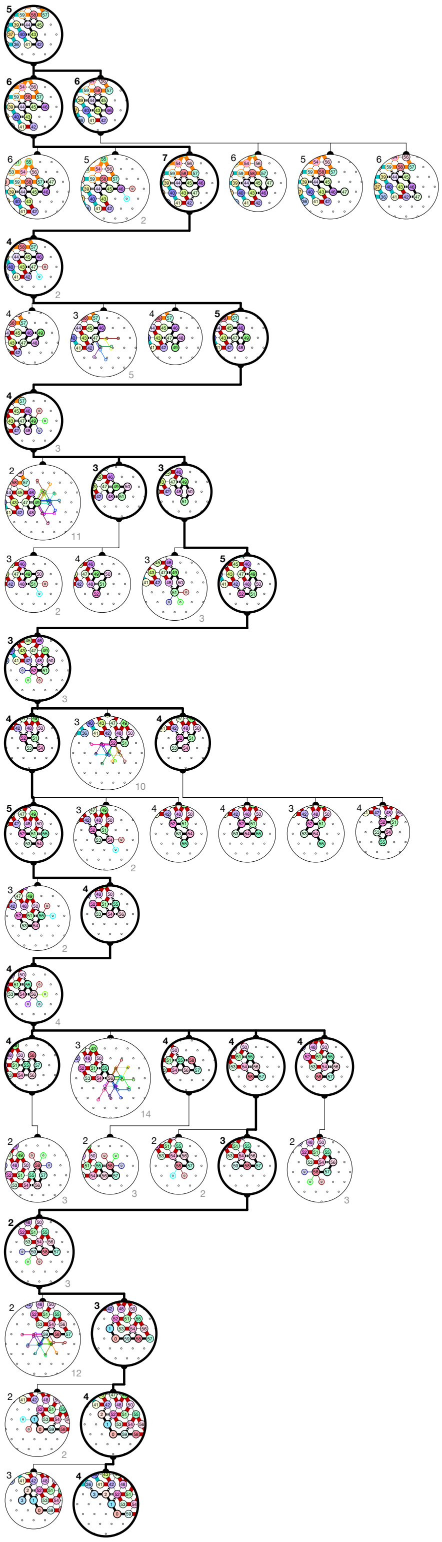

Output nascent configurations: α

Figure S.67. Folding of Brick DT in 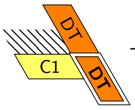

–First occurrence in 5-bits counter: Region #20.

S.3. Analysis of Algorithms 1 and 2

Proof of Theorem 3. The key is that every step of the folding is computed locally in a fixed and known environment: at each step i, the δ beads to be folded look for their best positions by interacting with beads with fixed and known positions within a radius $\delta + 1$. It follows that one can compute the set of all suitable subrules, considering these $O(\delta^2)$ bead types only (i.e., that place the $i - \delta + 1$-th bead of the molecule at the correct position). Oblivious $\mathscr{O}$ and inertial $\mathscr{I}$ dynamics differ as $\mathscr{I}$ only consider input nascent configurations which are output by the previous step, whereas $\mathscr{O}$ does not use any information from the previous step. This implies that one need to remember some information to connect one step to the next in $\mathscr{I}$, whereas $\mathscr{O}$ needs no memory at all.

Formally, a *subrule* $R : B^2 \to \{\texttt{true}, \texttt{false}, \perp\}$ is a symmetric function that states for each pair of beads if they attract each other (`true`) or not (`false`), or if this is undefined ($\perp$). We denote by $\operatorname{dom} R = \{(a, b) \in B^2 : R(a, b) \neq \perp\}$ the *domain* of R. Two subrules R_1 and R_2 are *compatible*, denoted by $R_1 \sim R_2$ if they agree for every pair where they are both defined, i.e., if for all $a, b \in \operatorname{dom} R_1 \cap \operatorname{dom} R_2$, $R_1(a, b) = R_2(a, b)$. We say that R_1 *matches with* R_2 if for all $(a, b) \in \operatorname{dom} R_2$, $R_1(a, b) = R_2(a, b)$. If $R_1 \sim R_2$, we denote by $R_1 \cup R_2$ the subrule obtained by *merging* R_1 and R_2, i.e. defined by $R_1 \cup R_2\,(a, b) = R_1(a, b)$ if $(a, b) \in \operatorname{dom} R_1$, and $= R_2(a, b)$ otherwise.

For all $0 \leqslant 1 \leqslant |c|$, we denote by $c_{1..i}$ the prefix of length i of a configuration c. Algorithm 1 solves RDP for the oblivious dynamics in time linear in the length of the sequence as follows. It incrementally constructs a set of rules that place each bead at its desired position in each of the k environments. It proceeds by maintaining a set $\mathcal{R}$ of candidate subrules that place correctly the first i beads in every of the k environments. At the i-th step, the main procedure FINDRULEOBLIVIOUS() first extends each candidate subrule R in $\mathcal{R}$ by calling procedure EXTENDOBLIVIOUS() which scans all the possible attraction rule extensions of R for the new nascent bead (the $(i + \delta - 1)$-th) with the bead types it can reach in each of the k configurations, and retains only the ones that place the i-th bead at its correct position for all k configurations. Note that this extension of the rule does not change the positioning of the $(i - 1)$th first beads since the $(i + \delta - 1)$-th bead is not yet produced when they are placed. Now, in order to keep the processing time constant for each bead, main procedure FINDRULEOBLIVIOUS() calls procedure PROJECTOBLIVIOUS() which retains only one representative of each subset of rules that define the same attractions for the $\delta - 1$ nascent beads (indexed from $i + 1$ to $i + \delta - 1$), i.e., for the only bead types for which the rule matters in order to determine the positions of the upcoming beads. Once the $n - \delta$-th bead is placed, the main procedure concludes by checking that the surviving rules place the last $\delta - 1$ beads at their desired positions.

Time and space complexity analysis. Let us first analyse the procedure EXTENDOBLIVIOUS. All the beads reachable by the $(i + \delta - 1)$th bead are located at distance at most $\delta + 1$ from c^j_{i-1} in the j-th target environment. The size of $\mathcal{N}$ is thus at most $3k(\delta + 1)^2$. It follows that there are at most $2^{3k(\delta+1)^2}$ subrules ρ to consider. Testing each subrule takes $O(5^\delta)$ time and $O(\delta^2)$ space. It follows that each execution of procedure EXTENDOBLIVIOUS takes $O(5^\delta 2^{3k(\delta+1)^2})$ time and $O(\delta^2 2^{3k(\delta+1)^2})$ space.

Let us now analysis the procedure PROJECTOBLIVIOUS. Again, all the beads reachable by the $(i + \delta - 1)$th bead are located at distance at most δ from c^j_i in the j-th target environment. The size of $\mathcal{N}$ is thus at most $3k\delta^2$. It follows that there are at most $2^{3k(\delta-1)\delta^2}$ subrules ρ to consider. It follows that the size of the output set Π is bounded by $2^{3k(\delta-1)\delta^2}$. Procedure PROJECTOBLIVIOUS runs then in $O(|\mathcal{R}| + 2^{3k(\delta-1)\delta^2})$ and uses at most $O(\delta^2 2^{3k(\delta-1)\delta^2})$ space.

Note that the size of $\mathcal{R}$ in the main procedure FINDRULEOBLIVIOUS is always bounded by the size of the output Π of PROJECTOBLIVIOUS times the size of the set Σ output of of EXTENDOBLIVIOUS. The size of $\mathcal{R}$ is thus at most $2^{3k(\delta^3+2\delta+1)}$ at all time. As the main procedure calls $n - \delta + 1$ times the procedures EXTENDOBLIVIOUS and the procedures PROJECTOBLIVIOUS, we conclude that Algorithm 1 runs in $O(n \cdot 5^\delta 2^{3k(\delta^3+2\delta+1+(\delta+1)^2)})$ time and uses $O(n \cdot \delta^2 2^{3k(\delta^3+2\delta+1+(\delta+1)^2)})$ space, which are both linear in n for constant k and δ.

Algorithm 2 works similarly for the inertial dynamics $\mathscr{I}$. In fact, we just extend the subrule technics by testing subrules for each possible input and corresponding output nascent configurations set, for each seed-target configurations pair and each time step. This multiplies the memory and time complexities by $2^{k5^{\delta-1}}$. □

© 2019 by the authors. Licensee MDPI, Basel, Switzerland. This article is an open access article distributed under the terms and conditions of the Creative Commons Attribution (CC BY) license (http://creativecommons.org/licenses/by/4.0/).
